# Effects of Computerized Cognitive Training on Vesicular Acetylcholine Transporter Levels using [18F]Fluoroethoxybenzovesamicol Positron Emission Tomography in Healthy Older Adults: Results from the Improving Neurological Health in Aging via Neuroplasticity-based Computerized Exercise (INHANCE) Randomized Clinical Trial

**DOI:** 10.2196/75161

**Published:** 2025-10-13

**Authors:** Mouna Attarha, Ana de Figueiredo Pelegrino, Lydia Ouellet, Paule-Joanne Toussaint, Sarah-Jane Grant, Thomas Van Vleet, Etienne de Villers-Sidani

**Affiliations:** 1 Posit Science Corporation San Francisco, CA United States; 2 Department of Neurology and Neurosurgery McGill University Montreal, QC Canada

**Keywords:** fluoroethoxybenzovesamicol, positron emission tomography, PET, FEOBV-PET, cognitive training, brain training, neuroplasticity, neuromodulatory signaling, aging, anterior cingulate

## Abstract

**Background:**

The cholinergic system mediates essential aspects of cognitive function, yet its structure and function decline progressively with age, by an estimated 2.5% per decade across the lifespan. Cognitive training may help counteract age-related declines in cholinergic functioning and slow associated deficits in cognitive performance.

**Objective:**

This study aims to evaluate whether cognitive training modifies cholinergic binding in older adults.

**Methods:**

The Improving Neurological Health in Aging via Neuroplasticity-based Computerized Exercise (INHANCE) trial is a double-blind randomized controlled trial assessing whether 2 computerized cognitive training programs modify cholinergic expression. The intent-to-treat (ITT) population included 92 community-dwelling healthy older adults aged 65 and above (enrolled July 2021-December 2023; final follow-up June 2024). Participants were randomized at McGill University to either an intervention of speed-based cognitive training exercises designed to improve the speed and accuracy of information processing or an active control of nonspeeded games designed for entertainment (eg, similar in design to Solitaire). Participants completed 35 hours of training on their assigned program at home over a 10-week period using a loaned or personal internet-connected device. Cholinergic binding was measured with the vesicular acetylcholine transporter ligand [^18^F]fluoroethoxybenzovesamicol (FEOBV) and positron emission tomography (PET). The primary outcome was mean FEOBV binding (standard uptake value ratios [SUVRs]) within the anterior cingulate cortex from baseline to posttest in the ITT population. All other end points were exploratory.

**Results:**

Among the 92 participants in the ITT population (mean age 71.9 years; mean education 16.5 years; 61/92, 66%, women; 88/92, 96%, White), 82 (89%) completed all study activities. The speed-based intervention showed a significant within-group increase in FEOBV binding in the primary region of interest, the anterior cingulate cortex (SUVR change mean +0.044, 95% CI 0.006-0.082, P=.03, medium effect size, ω²=0.09). The p24c subregion demonstrated a significant between-groups effect favoring speed training (speeded vs nonspeeded SUVR change difference +0.058, 95% CI 0.007-0.110, P=.03, small effect size, ω²=0.05). Prespecified exploratory analyses revealed significant within-group effects for speed training in the hippocampus (P=.02) and parahippocampal gyrus (P=.04). No effects on FEOBV binding were observed in the active control group.

**Conclusions:**

INHANCE is the largest FEOBV-PET trial to date and demonstrates, for the first time in humans, that speed training can reverse losses in cholinergic terminal densities in brain regions vulnerable to age-related cognitive decline. The 2.3% gain in FEOBV binding in the anterior cingulate achieved over a 10-week intervention may offset the estimated 2.5% decline typically observed over a decade of natural aging. These findings clarify the neurochemical basis of cognitive training benefits, showing that speed training upregulates binding in networks that support attention, memory, and executive function.

**Trial Registration:**

ClinicalTrials.gov NCT04149457; https://clinicaltrials.gov/study/NCT04149457

**International Registered Report Identifier (IRRID):**

RR2-10.2196/59705

## Introduction

### Background

Aging is the strongest known risk factor for dementia, with the risk doubling approximately every 5 years beyond the age of 65 [1]. At the neurological level, aging induces significant system-wide alterations, including decreased or impaired signaling from neuromodulatory centers that gate synaptic plasticity [2-6], expansion of spatiotemporal receptive-field size [7], increased temporal integration windows [8], and elevated neuronal noise [9]. These changes hinder the speed and accuracy of information processing across sensory modalities and compromise the precision and reliability of representational input, which in turn affects the operation of higher-order cognitive functions [10]. Deficits in learning, memory, and executive function are the end products of neurochemical downregulation and the slowed, imprecise functioning of sensory networks that have lost the ability or plasticity to adapt to environmental demands [11].

Research in both animal and human models has consistently highlighted acetylcholine as a pivotal mediator of synaptic plasticity essential for cognition [5,12-14]. Experimental evidence shows that stimulating the release of acetylcholine, such as through the nucleus basalis, improves cognitive processes such as attention, making it an important regulator of learning rate, memory formation, and memory retrieval across sensory modalities [2,3]. Conversely, lesioning or inhibiting acetylcholine impairs plasticity and cognitive performance [4]. Disruptions in cholinergic signaling are linked to age-related memory deficits that precede the early stages of pathological cognitive decline. These disruptions initiate a signaling cascade that interacts with neuropathologies such as amyloidogenesis and tau phosphorylation, ultimately contributing to cholinergic network atrophy, a hallmark of neurodegenerative diseases [15-21].

Clinically, therapeutic approaches aimed at augmenting cholinergic function with cholinesterase inhibitors (donepezil, rivastigmine, and galantamine) have been used to treat mild cognitive impairment (MCI), Alzheimer disease, and related dementias [22], underscoring the significance of neuromodulators such as acetylcholine in preserving the chemical integrity of the brain [6,23-25]. Recent advancements now enable noninvasive, in vivo measurement of cholinergic network integrity in the human brain using positron emission tomography (PET). The radioligand [^18^F]fluoroethoxybenzovesamicol (FEOBV) selectively binds to the vesicular acetylcholine transporter (VAChT), a protein uniquely expressed by cholinergic neurons [26,27]. The binding pattern across cortical and subcortical areas is consistent with the organization of the cholinergic system and aligns with immunohistology from prior postmortem human studies [28]. Declines in binding are detectable in brain regions known to have reduced cholinergic terminal densities due to aging [29], MCI [30], and dementia [31].

Aging is associated with significant declines in FEOBV binding, with reductions estimated at 2.5% per decade between the ages of 20 and 80 in the anterior cingulate cortex [29], a region that plays a critical role in selective attention [32], learning and memory [33], and executive function [34]. Metabolic dysfunction in this area frequently precedes the earliest cognitive changes observed across the lifespan, and the degree of hypometabolism has been correlated with greater cognitive decline in otherwise healthy adults [35]. Moreover, increased anterior cingulate thickness has been linked to successful cognitive aging [36,37], whereas atrophy in this region serves as a predictor of future dementia development [38].

Decades of research in animal models have shown that cholinergic function can be upregulated by specific behavioral strategies such as cognitive training. Identifying effective behavioral interventions that support cognition in older adults has become an important area of study in recent years [39]. The National Academies of Sciences, Engineering, and Medicine (NASEM) published a review on primary prevention factors and interventions that may independently delay, slow, or prevent cognitive decline. Their report arrived at 3 recommendations (cognitive training, maintaining normal blood pressure, and physical exercise) as having sufficient evidence that “the public should at least have access to these results to help inform their decisions about how they can invest their time and resources to maintain brain health with aging” [40]. In the same year, the American Academy of Neurology (AAN) convened an expert panel and updated its guidelines, stating that clinicians may recommend cognitive training for those with MCI [41]. Most recently, the World Health Organization (WHO) published its 2024 practice guidelines recommending cognitive training as an evidence-based intervention for individuals with dementia [42].

The collective results of the largest trial in cognitive training to date, the Advanced Cognitive Training for Independent and Vital Elderly (ACTIVE), provide strong evidence that a specific type of cognitive training yields sustained cognitive and functional benefits that generalize beyond the trained task. A selective attention visual speed-of-processing training exercise, referred to as “speed training” in the original publications (now Double Decision, part of the BrainHQ program), reduced dementia incidence by 29%-48% over a decade-long follow-up, depending on the number of training hours completed [43]. Additional studies of speed training have reported improved driving safety [44] and a 48% reduction in at-fault motor vehicle collisions [45], improved balance [46] and a 31% reduction in falls among those at high risk [47], slowed decline in instrumental activities of daily living maintained for 10 years postintervention [48-52], a 68% greater likelihood of improved locus of control maintained over 5 years [53], decreased predicted health care payer–related costs [54], a 38% reduction in the onset of age-related depression [55], a 30% reduction in depressive symptoms [56], and a reduced risk of global decline in health-related quality of life maintained at the 5-year follow-up [57,58].

Another form of training, called Freeze Frame (BrainHQ), was designed to engage tonic and phasic alertness to naturally upregulate neuromodulatory control and improve the accuracy of information processing under speeded conditions. Initial studies demonstrated benefits in executive function, skill acquisition, and spatial and nonspatial attention [59-63], as well as enhancement of the training gains achieved with Double Decision [59]. In a pilot substudy of the phase II ALERT trial, 5 healthy older adults training on Freeze Frame showed a 16%-24% increase in forebrain cholinergic neurotransmission, as measured by FEOBV-PET. These increases in binding paralleled behavioral gains on a sustained vigilance assessment [64].

The design principles of these speeded cognitive training exercises are grounded in a theoretical framework discussed previously [11,65,66]. The neurobiological mechanisms underlying the generalized benefits of these forms of cognitive training, however, remain largely unknown. Several pilot studies have provided initial insights, suggesting that potential mechanisms may include improved functional connectivity within the default mode and central executive networks [67-69], enhanced brain synchronization [70], improved functional magnetic resonance imaging (MRI) and electroencephalography measures of brain activation [69-72], increased hippocampal activation [73], and improved diffusion tensor imaging measures of white matter insulation between brain regions involved in visual and attentional processing [74].

At the core of these network-based changes is synaptic plasticity, a fundamental process regulated by multiple neuromodulatory centers in the brain, including the basal forebrain, which sends cholinergic projections throughout the brain [4,5]. These neuromodulatory systems in general, and cholinergic systems in particular, have long been considered potential drivers of the benefits of computerized training programs and a novel mechanism through which such programs may support brain health and cognition. With the recent development of the FEOBV-PET method for quantitatively assessing cholinergic system integrity in humans, and evidence that analogous forms of cognitive training in rodents alter cholinergic function [75], we hypothesized that cognitive training exercises in humans (Double Decision and Freeze Frame) would affect cholinergic systems. Such findings would inform the neural mechanisms underlying speed-based cognitive training programs and open new avenues of research for developing effective cognitive training interventions.

### The Proposed Study

The Improving Neurological Health in Aging via Neuroplasticity-based Computerized Exercise (INHANCE) trial is a randomized controlled study of healthy, community-dwelling older adults aged 65 and above. Its objective was to advance understanding of the mechanisms underlying cognitive training–related benefits in aging by examining neurochemical dynamics associated with cognition. Specifically, INHANCE aims to (1) evaluate, on a primary basis, the effects of 2 computerized training programs on cholinergic binding using FEOBV-PET; (2) assess cognitive and behavioral performance on an exploratory basis; and (3) determine the maintenance of training effects.

Participants were assigned either to speeded cognitive training (Double Decision and Freeze Frame exercises) or to an active control condition involving nonspeeded games designed for entertainment (eg, spin-offs of Solitaire and Candy Crush, titled Double Klondike Solitaire and Bricks Breaking Hex, respectively). The primary end point was FEOBV binding (standard uptake value ratios [SUVRs]) in the anterior cingulate cortex from baseline to posttest for the intent-to-treat (ITT) population. We hypothesized that speed training would result in greater FEOBV binding at posttest compared with baseline.

All other end points were exploratory. Given the extensive projections of cholinergic neurons from the basal forebrain to the limbic system and neocortex, FEOBV SUVRs were evaluated across additional regions of interest: global cortex, frontal lobe, parietal lobe, occipital lobe, temporal lobe, hippocampus, parahippocampal gyrus, striatum, putamen, caudate, posterior cingulate cortex, primary auditory cortex, primary sensorimotor cortex, and the nucleus basalis of Meynert. Participants also completed 3 subtests from the National Institutes of Health The Executive Abilities: Measures and Instruments for Neurobehavioral Evaluation and Research (NIH EXAMINER) cognitive battery to evaluate cognitive control, 2 train-to-task assessments to evaluate target engagement [76,77], and 2 behavioral assessments sensitive to cholinergic function (heart rate variability and pupillometry). All cognitive and behavioral assessments were administered at baseline, posttest, and at a no-contact 3-month follow-up to measure the maintenance of observed effects.

## Methods

### Ethical Considerations

The study was conducted in accordance with the Declaration of Helsinki and approved by the Western Institutional Review Board (IRB00000533) and the Research Ethics Board of McGill University Health Centre (2020-6474). The FEOBV radioligand was approved by Health Canada (Control #252085). All participants provided written informed consent (see Multimedia Appendix 1).

The consent form described the study as evaluating the efficacy of 2 cognitive training interventions: one focused on “speed” and the other on “executive function.” To maintain blinding, both programs were accessed by participants through the same commercial website (web) and commercial app (mobile).

Study data were recorded into a secure, web-based electronic case report form at the study site through the Longitudinal Online Research and Imaging System (LORIS). This system complies with relevant privacy and security standards for electronic trial data entry and storage, as well as the Health Insurance Portability and Accountability Act and the Personal Information Protection and Electronic Documents Act standards for confidentiality and privacy [78]. Following consent, each participant was assigned a standardized Participant Identification Number, composed of digits identifying the study and digits identifying the participant. All electronic case report form entries were deidentified, using the Participant Identification Number rather than the participant’s name.

Participants received US $30 (CAD $40) for completing the baseline visit, which included PET and MRI (visit 1); US $10 (CAD $13) for every 10 training sessions completed during the intervention period (up to US $70 [CAD $91] for all 70 sessions); US $30 (CAD $40) for completing the posttest visit, which included PET and MRI (visit 2); and US $30 (CAD $40) for the 3-month end-of-study follow-up visit (visit 3). Participants who completed all study activities were reimbursed a total of US $160 (CAD $211). Payments were provided following completion of each visit. Additional details are available in the study protocol [79].

### Study Design

INHANCE is a prospective, double-blind, parallel-arm, active-controlled randomized clinical trial in healthy adults aged 65 and above. Participants were randomized to receive 35 hours of either a computerized visual speed-based cognitive training program (BrainHQ) or an active control consisting of visual nonspeeded computerized games over a 10-week period. A detailed description of the outcome measures, training programs, and statistical analysis plan is provided in the study protocol [79] (see also Multimedia Appendix 2).

### Screening

Following informed consent, potential participants completed an in-person structured interview and neuropsychological assessments to determine study eligibility. The structured interview (approximately 20 minutes, in person) was conducted by a research coordinator and included demographic information (eg, year of birth, age, education), medical diagnoses, and current medications, guided by administrator-facing source documents. Neuropsychological assessments were participant-facing. The Montreal Cognitive Assessment (MoCA; about 10 minutes) evaluated global cognitive function across visuospatial processing, executive functioning, naming, memory, attention, language, abstraction, delayed recall, and orientation, yielding a total score of 30, with higher scores indicating better performance [80]. The Geriatric Depression Scale—Short Form (GDS-SF; about 7 minutes) assessed self-reported depressive symptoms, with participants responding yes or no to 15 items about how they felt during the past week [81,82]. The Columbia Suicide Severity Rating Scale (about 10 minutes) assessed the severity and intensity of suicidal ideation, as well as suicidal behavior and lethality [83] (see Multimedia Appendix 3). For additional details on the validation, administration, and adjudication of screening measures, see the study protocol [79].

### Participants and Inclusion and Exclusion Criteria

Participants were community-dwelling healthy older adults. Inclusion criteria required individuals to be aged 65 or older, proficient in English or French, capable of fulfilling study requirements, and cognitively intact, defined as an MoCA total score of ≥23, a cut-off that optimizes diagnostic sensitivity and specificity [84]. Exclusion criteria included neurocognitive disorders, suicidal ideation, major depression indicated by a GDS-SF total score >10, prior experience using BrainHQ within the past 5 years, pregnancy, substance abuse, or contraindications to neuroimaging. Concurrent participation in clinical trials involving investigational devices, use of medications with established cholinergic effects [85], or medical conditions that could hinder study engagement were also exclusionary at the discretion of the site investigator (EDVS). Full details are provided in the study protocol [79].

### Protocol Changes

Enrollment spanned from July 2021 to December 2023. In October 2022, the protocol and recruitment flyer were revised to state, “Potential participant must be able to communicate in either English or French” (originally, “Potential participant must be a fluent English or French speaker from the age of 12”), as the original wording unintentionally excluded minority groups. This modification to the inclusion criteria was approved by the study’s Data Safety Monitoring Board, the Western Institutional Review Board, and the Research Ethics Board of McGill University Health Centre in January 2023.

### Recruitment

All participants were recruited near McGill University, Canada, where the FEOBV radiotracer was synthesized and administered. Recruitment methods included public presentations (television, radio, conferences), word of mouth, and flyers. Flyers were posted in churches, community centers, Facebook groups, local libraries, and the neurology clinic at the Montreal Neurological Hospital. They described the study and provided information on inclusion and exclusion criteria, along with the study team’s phone number and email address. All study flyers received ethics approval before distribution (see Multimedia Appendix 4).

### Randomization

We employed a minimization method of adaptive stratified randomization with a 1:1 allocation to either the intervention or active control group. Groups were stratified based on baseline FEOBV and the baseline NIH EXAMINER composite score. After completion of baseline assessments, a blinded team member at the study site initiated a randomization request by entering both scores and setting the randomization status to “Awaiting Review” in LORIS, a data collection and management system with strict data access controls [86]. The request was then directed to an unblinded team member at the coordinating center (SJG), who reviewed and confirmed the accuracy of the participant’s scores and set the randomization status to “Reviewed,” triggering an automated script. The script cumulatively assessed overall study imbalance, and the final group assignment was made at random with a strong probability weighting (0.8) favoring the group that would minimize imbalance. Once the assignment was determined, an automated email containing the participant’s group allocation was sent from LORIS to an unblinded site team member, who then conducted the program orientation visit. All randomizations were completed before the participant’s first day of program use.

### Intervention and Active Control Training Programs

**Figure 1:**
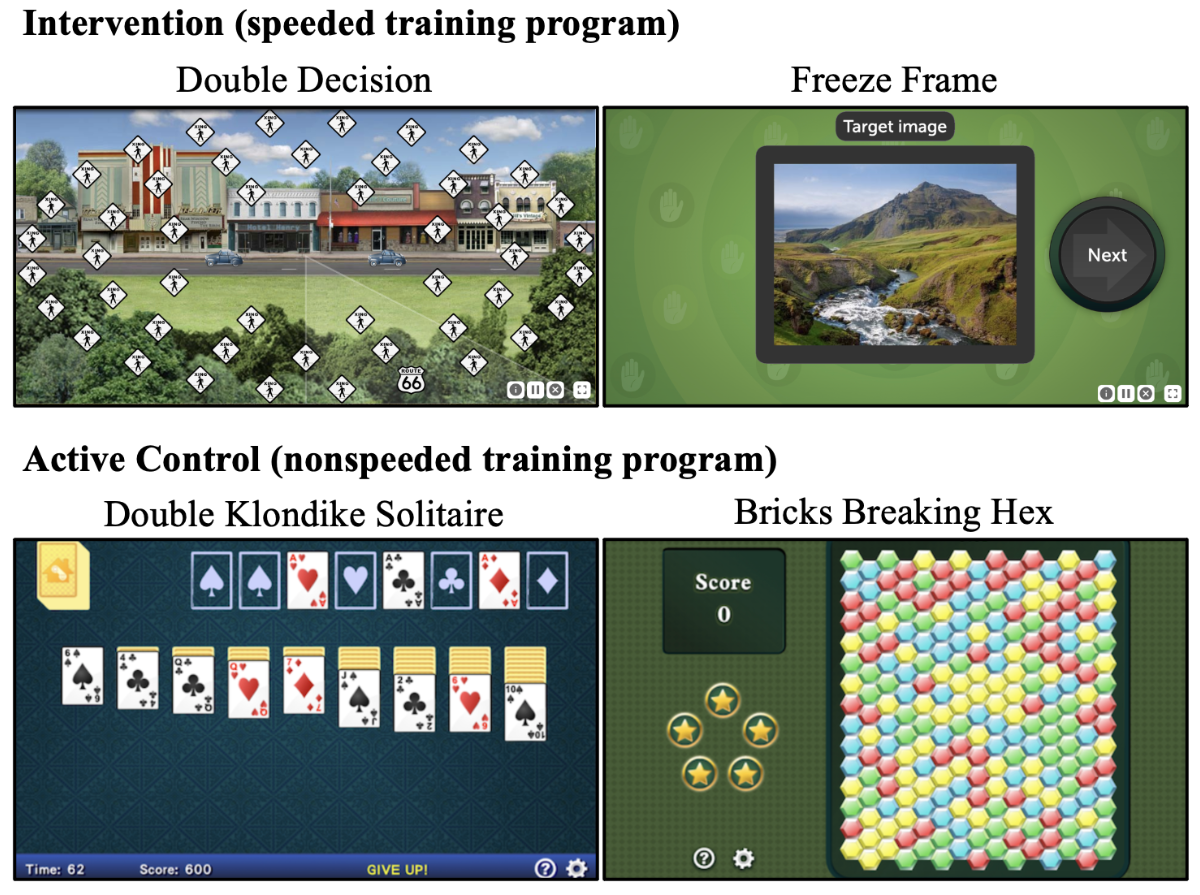
Intervention and active control programs. The intervention included 2 tasks: Double Decision, a dual-task paradigm in which participants discriminate a centrally presented visual stimulus while simultaneously locating a target in the peripheral visual field across 40 unique levels, with display exposure duration as the adaptive dimension; and Freeze Frame, a speeded reverse go/no-go paradigm in which participants remember a target image presented at trial onset, followed by a continuous stream of targets and foils with unequal probability, withholding responses to targets and responding quickly to foils, with difficulty as the adaptive dimension. The active control included spin-offs of casual games: Double Klondike Solitaire, in which participants move cards to 8 foundations by suit from Ace to King, and Bricks Breaking Hex, in which participants eliminate groups of same-colored bricks by clicking them.

Participants received either visual speed training (Double Decision and Freeze Frame exercises) or an active control consisting of visual nonspeeded computer games designed for entertainment (Double Klondike Solitaire and Bricks Breaking Hex). Both training programs involved 35 hours of computerized training delivered over a 10-week period (approximately 30 minutes per session, 7 sessions per week, for a total of 70 sessions). Training was completed remotely at participants’ homes using either a loaned Android tablet or a personal internet-connected device. Participants accessed their assigned program via an identical website or app and were blinded to group assignment (see Figure 1).

The active control was designed to account for nonspecific treatment effects, including placebo and practice effects, face validity, interactions with research personnel, computer experience, and was matched to the intervention in overall program use intensity, visual modality, platform delivery, reward delivery, and remuneration of time [87-89]. The control program was strategy-based and did not include speeded elements hypothesized to drive positive neuroplastic changes in the intervention. Control games were developed by an independent gaming company and rated E (for Everyone) by the Entertainment Software Rating Board.

### Outcomes

All outcomes were measured in person by blinded assessors. The primary outcome was FEOBV binding, quantified using mean SUVRs in the primary region of interest (ROI), the anterior cingulate cortex, between baseline and posttest for the ITT population.

FEOBV binding was also assessed in prespecified exploratory regions of interest, including the global cortex (frontal, parietal, occipital, and temporal lobes), hippocampus, parahippocampal gyrus, striatum (putamen and caudate), posterior cingulate cortex, primary auditory cortex, primary sensorimotor cortex, and the nucleus basalis of Meynert, which provides the major source of cholinergic innervation to the cerebral cortex. In the nucleus basalis of Meynert, FEOBV SUVR likely reflects a combination of tracer binding to cholinergic cell bodies and cholinergic brain stem afferents terminating in this region.

A structural MRI (3T Siemens MAGNETOM Prisma; 20 minutes) was acquired during the first scanning session to coregister the PET images (Siemens High-Resolution Research Tomograph; see Multimedia Appendix 2). Cognitive control was assessed using 3 computerized subtests (Flanker, Set-Shifting, and Anti-Saccades) from a validated neuropsychological battery, with performance summarized as the *z* score of the executive composite from NIH EXAMINER [90] (20 minutes). Participants also completed 2 train-to-task cognitive assessments (the Double Decision assessment and the Freeze Frame assessment [15 minutes]) modeled directly on the 2 intervention exercises, with performance summarized as *z* scores to evaluate target engagement. Behavioral measures sensitive to acetylcholine function were also collected, including heart rate variability (using a Shimmer 4-lead Consensys ECG Development Kit) and pupillometry (using Tobii Pro Glasses 2). All cognitive and behavioral assessments were conducted at baseline, posttest, and at a 3-month no-contact follow-up. Additional details are provided in the study protocol [79].

### Power and Sample Size Calculations

Based on the 0.27 SD observed in anterior cingulate FEOBV binding in a single-arm, open-label pilot study [64] (N=5), detecting a small-to-moderate effect size (17% increase in anterior cingulate FEOBV binding postintervention) with 80% power and a significance level of .05 required 40 participants per group (80 total). Assuming 15% attrition, at least 92 participants were enrolled to ensure a minimum of 80 completers to adequately power the primary outcome. No interim analyses or stopping guidelines were planned.

### Statistical Analyses

A detailed scan protocol, including data acquisition and processing, is provided in the published protocol [79] (see also Multimedia Appendix 2). Briefly, the statistical analysis plan defined a primary ITT population, a single primary outcome measure, a set of exploratory outcome measures, a primary evaluation time point, an exploratory evaluation time point, a primary statistical analysis methodology, criteria for statistical significance, and guidance for result interpretation.

The ITT population was defined a priori as participants who were randomized and completed at least one training session postrandomization (30 minutes).

The primary statistical analysis employed a linear mixed-effects model. Intervention and active control groups in the ITT population were first compared at baseline to identify potential covariates [91]. Each outcome measure was then analyzed using a linear mixed-effects model with treatment group and time as fixed factors, incorporating covariates as needed based on the baseline analysis. Within-group effects at each time point (posttraining and follow-up) were calculated using data from each group, while between-group effects were assessed by including an interaction term (training group × time) to estimate the effect of cognitive training on outcome changes.

Analyses in this study were conducted in accordance with the published protocol, with the exception of additional analyses suggested by anonymous reviewers of related INHANCE trial publications, including a baseline cognition subgroup analysis using the MoCA.

## Results

### Enrollment

The study was conducted from 2019 to 2024 at McGill University, Canada. Recruitment occurred from July 2021 to December 2023, and the final follow-up visit took place on June 7, 2024.

**Figure 2:**
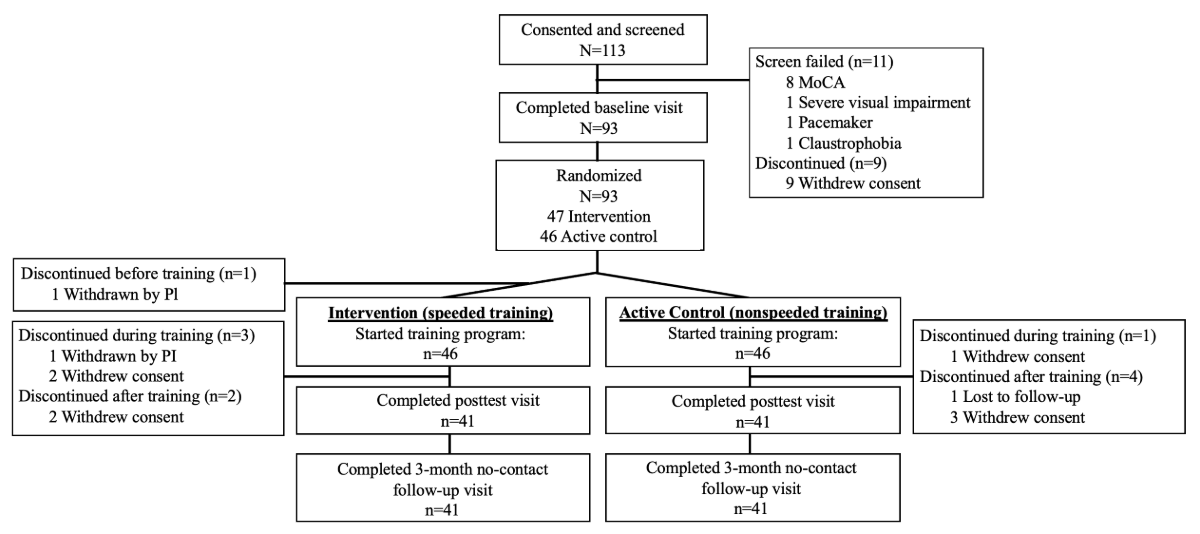
CONSORT (Consolidated Standards of Reporting Trials) flowchart. Participant flow through the trial. The intention-to-treat (ITT; N=92) population was defined a priori as all randomized participants who completed at least one training session. MoCA: Montreal Cognitive Assessment; PI: principal investigator.

A total of 113 individuals were screened and provided written informed consent; of these, 20 did not qualify or chose not to participate further. Ninety-three participants completed baseline assessments and were randomized to either the intervention group (speeded training, n=47) or the active control group (nonspeeded training, n=46), with 1 participant withdrawn by the site principal investigator (EDVS) before engaging with the intervention. The ITT population (N=92) included 46 participants in each arm and was analyzed (mean age 71.9 years; mean education 16.5 years; 61/92, 66%, women; and 88/92, 96% White). Overall, 41 out of 46 participants in each arm (or 82/92 combined, 89%) completed both the posttest assessments and the 3-month no-contact follow-up visit. See Figure 2 for the CONSORT (Consolidated Standards of Reporting Trials) flowchart and Multimedia Appendix 5 for the CONSORT checklist.

### Baseline Characteristics of the ITT Population

Participant characteristics for the ITT population are presented in Table 1. Baseline characteristics were well-balanced between the intervention and active control groups, differing statistically only for the baseline Double Decision assessment, which was included as a covariate in the model (t_90_=–2.073, *P*=.04). Overall, participants were cognitively normal, without depression, college-educated, and predominantly female and White. Additional baseline characteristics of the sample have been reported previously [92].

Participants randomized to the intervention group (n=46) had a mean NIH EXAMINER executive composite score of 0.38 (SD 0.64). On the Double Decision train-to-task assessment, the mean raw score was 1632.46 (SD 883.24) ms, with lower scores indicating better performance. On the Freeze Frame train-to-task assessment, the mean raw score was 4.22 (SD 1.99), with higher scores indicating better performance.

Participants randomized to the active control group (n=46) had a mean NIH EXAMINER executive composite score of 0.49 (SD 0.50). On the Double Decision train-to-task assessment, the mean raw score was 1320.93 (SD 712.12) ms, and on the Freeze Frame train-to-task assessment, the mean raw score was 5.11 (SD 2.09).

**Table 1:** Baseline demographic, inclusion, and outcome measures of the ITT^a^ population (N=92) by treatment group.

Baseline characteristics		Intervention (n=46)	Active control (n=46)	*P* value
**Demographics**				
	Age, mean (SD), years	72.76 (5.25)	71.04 (4.32)	.09
	Education, mean (SD), years	16.28 (3.53)	16.63 (3.30)	.63
	Gender (female), n (%)	29 (63)	32 (70)	.66
	Race (White), n (%)	44 (96)	44 (96)	.26
	Ethnicity (Hispanic or Latino), n (%)	0 (0)	0 (0)	.99
	Native language (English), n (%)	17 (37)	16 (35)	.62
**Inclusion**				
	MoCA^b^ (total score), mean (SD)	26.37 (1.83)	25.98 (1.84)	.31
	GDS-SF^c^ (total score), mean (SD)	1.24 (1.85)	1.52 (1.91)	.47
**Primary measure**				
	FEOBV^d^ anterior cingulate cortex, SUVR^e^ (SD)	1.896 (0.202)	1.911 (0.186)	.72
**Exploratory measures**				
	FEOBV global cortex (SUVR), mean (SD)	1.416 (0.164)	1.459 (0.122)	N/A^f^
	FEOBV frontal lobe (SUVR), mean (SD)	1.648 (0.203)	1.693 (0.139)	N/A
	FEOBV parietal lobe (SUVR), mean (SD)	1.450 (0.177)	1.465 (0.149)	N/A
	FEOBV occipital lobe (SUVR), mean (SD)	1.172 (0.187)	1.238 (0.149)	N/A
	FEOBV temporal lobe (SUVR), mean (SD)	1.395 (0.194)	1.440 (0.152)	N/A
	FEOBV hippocampus (SUVR), mean (SD)	2.094 (0.315)	2.165 (0.255)	N/A
	FEOBV parahippocampal gyrus (SUVR), mean (SD)	1.361 (0.235)	1.413 (0.179)	N/A
	FEOBV striatum (SUVR), mean (SD)	5.972 (0.850)	6.220 (0.770)	N/A
	FEOBV putamen (SUVR), mean (SD)	6.938 (1.043)	7.207 (0.905)	N/A
	FEOBV caudate (SUVR), mean (SD)	5.006 (0.801)	5.232 (0.696)	N/A
	FEOBV posterior cingulate (SUVR), mean (SD)	1.942 (0.192)	1.939 (0.205)	N/A
	FEOBV primary auditory cortex (SUVR), mean (SD)	1.958 (0.317)	2.071 (0.284)	N/A
	FEOBV primary sensorimotor cortex (SUVR), mean (SD)	1.737 (0.227)	1.717 (0.201)	N/A
	FEOBV nucleus basalis of Meynert (SUVR), mean (SD)	3.281 (0.568)	3.391 (0.453)	N/A
	NIH EXAMINER^g,h^ (composite *z* score), mean (SD)	–0.10 (1.12)	0.10 (0.87)	.34
	Double decision train-to-task assessment (*z* score), mean (SD)	–0.19 (1.09)	0.19 (0.88)	.04
	Freeze frame train-to-task assessment (*z* score), mean (SD)	–0.22 (0.96)	0.21 (1.01)	.07

^a^ITT: intent to treat.

^b^MoCA: Montreal Cognitive Assessment.

^c^GDS-SF: Geriatric Depression Scale—Short Form.

^d^FEOBV: [^18^F]fluoroethoxybenzovesamicol.

^e^SUVR: standard uptake value ratio.

^f^N/A: not applicable.

^g^NIH EXAMINER: National Institutes of Health The Executive Abilities: Measures and Instruments for Neurobehavioral Evaluation and Research.

^h^Higher scores are better.

### Adherence to the Training Programs

Adherence was high throughout the trial, with 89 (97%) participants of the ITT population (N=92) completing the minimum of 10 hours of training, as informed by the ACTIVE study [43,49,93]. Among those randomized to the intervention group (n=46), 38 (83%) completed the prescribed 35 or more hours of training, with a mean of 40.81 hours. In the active control group (n=46), 35 (76%) completed the prescribed 35 or more hours, with a mean of 65.80 hours.

### Missing Data

A total of 10 (11%) participants from the ITT population (N=92) had missing MRI/PET, NIH EXAMINER, and train-to-task assessment data at posttest and follow-up due to study withdrawal before the posttest visit (5 from the intervention group and 5 from the active control group).

In addition to the participants who withdrew, data from a small number of participants were missing or invalid for technical reasons. Of note, 3 participants (3%) who completed the study had exceptionally poor atlas alignment, preventing valid calculation of FEOBV SUVRs (1 intervention participant at baseline and posttest; 2 controls at posttest only), 2 participants (2%) had corrupted MRI files at posttest (1 intervention and 1 control), 3 participants (3%) had invalid NIH EXAMINER scores at the 3-month follow-up due to administration of the incorrect form (2 intervention and 1 control), and 2 participants (2%) had a missing Freeze Frame train-to-task assessment due to technical issues (1 intervention at baseline and 1 control at post-test). The statistical model used iterative full-information maximum likelihood estimation to account for missing data.

### Training Effects on the Primary Outcome Measure

Table 2 presents FEOBV-PET SUVRs in the primary ROI, the anterior cingulate cortex, for both the intervention and active control groups at baseline and posttest. A significant within-group increase in SUVR was observed for the speeded intervention (mean change +0.044, 95% CI 0.006-0.082, *P*=.03, medium effect size ω²=0.09). No significant within-group change was observed for the nonspeeded active control (mean change +0.014, 95% CI –0.026 to 0.054, *P*=.50, effect size ω²<0.01). Tracer uptake in the white matter reference region did not differ between conditions (mean change –2.953, 95% CI –9.636 to 3.718, *P*=.39, effect size ω²<0.01).

**Figure 3:**
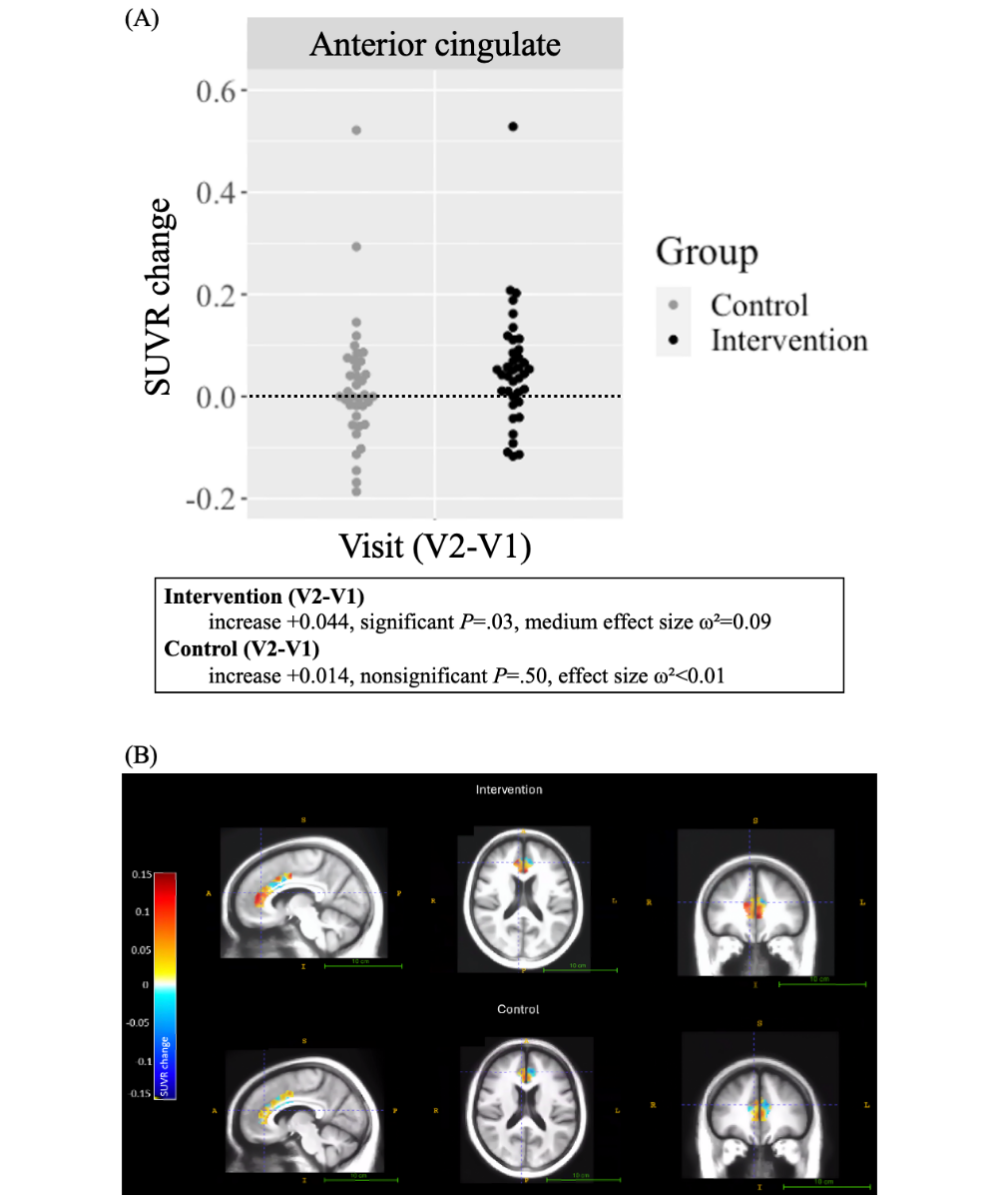
Standard uptake value ratio (SUVR) change within the primary region of interest (ROI). (A) SUVR binding change in the anterior cingulate at posttest (visit 2) relative to baseline (visit 1), with each icon representing a single participant’s change score. Higher scores indicate greater increases in binding. (B) SUVR binding change in the anterior cingulate cortex at posttest relative to baseline, averaged across participants in the intervention (top) and active control (bottom). Warmer colors indicate greater increases in binding. A: anterior; I: inferior; L: left; P: posterior; R: right; S: superior.

Figure 3 illustrates the change in SUVR from baseline to posttest for the intervention and active control groups. Separate SUVR images for each time point are provided in Figure S1 in Multimedia Appendix 2.

**Table 2:** Outcome measure analysis. Within- and between-group analyses for the ITT^a^ population (N=92) at posttest (visit 2) and follow-up (visit 3) for the intervention and active control groups.

Measures			Intervention within-group differences							Active Control within-group differences							Between groups differences					
			Visit 2 baseline, mean (SD)		Visit 2-visit 1 change, mean (95% CI), *P* value, effect size (ω^2^)^b^		Visit 3-visit 1 change, mean (95% CI), *P* value, effect size (ω^2^)		Visit 1 baseline, mean (SD)			Visit 2-visit 1 change, mean (95% CI), *P* value, effect size (ω^2^)		Visit 3-visit 1 change, mean (95% CI), *P* value, effect size		Visit 2-visit 1 change difference (95% CI), *P* value, effect size			Visit 3-visit 1 change difference (95% CI), *P* value, effect size			
**Primary measure**																						
	FEOBV^c^ anterior cingulate cortex (SUVR^d^)	1.896 (0.202)		+0.044 (0.006 to 0.082), .03^e^, 0.09		N/A^f^		1.911 (0.186)			+0.014 (–0.026 to 0.054), .49, <0.01		N/A		+0.030 (–0.025 to 0.085), .28, <0.01			N/A				
**Exploratory measures**																						
	FEOBV global cortex (SUVR)	1.416 (0.164)		+0.026 (–0.007 to 0.059), .13, 0.03		N/A		1.459 (0.122)			+0.011 (–0.020 to 0.042), .48, <0.01		N/A		+0.015 (–0.0230 to 0.060), .52, <0.01			N/A				
	FEOBV frontal lobe (SUVR)	1.648 (0.203)		+0.033 (–0.013 to 0.080), .17, 0.02		N/A		1.693 (0.139)			+0.009 (–0.023 to 0.040), .58, <0.01		N/A		+0.024 (–0.031 to 0.081), .40, <0.01			N/A				
	FEOBV parietal lobe (SUVR)	1.450 (0.177)		–0.003 (–0.035 to 0.028), .83, <0.01		N/A		1.465 (0.149)			+0.004 (–0.017 to 0.025), .71, <0.01		N/A		–0.008 (–0.045 to 0.030), .70, <0.01			N/A				
	FEOBV occipital lobe (SUVR)	1.172 (0.187)		+0.033 (–0.005 to 0.072), .10, 0.04		N/A		1.238 (0.149)			+0.012 (–0.033 to 0.057), .59, <0.01		N/A		+0.021 (–0.037 to 0.080), .49, <0.01			N/A				
	FEOBV temporal lobe (SUVR)	1.395 (0.194)		+0.043 (–0.002 to 0.089), .07, 0.06		N/A		1.440 (0.152)			+0.016 (–0.030 to 0.062), .49, <0.01		N/A		+0.027 (–0.037 to 0.092), .42, <0.01			N/A				
	FEOBV hippocampus (SUVR)	2.094 (0.315)		+0.089 (0.015 to 0.163), .02^g^, 0.10		N/A		2.165 (0.255)			+0.026 (–0.051 to 0.101), .50, <0.01		N/A		+0.061 (–0.044 to 0.167), .26, <0.01			N/A				
	FEOBV parahippocampal gyrus (SUVR)	1.361 (0.235)		+0.059 (0.005 to 0.114), .04^g^, 0.08		N/A		1.413 (0.179)			+0.021 (–0.032 to 0.072), .43, <0.01		N/A		+0.037 (–0.037 to 0.112), .33, <0.01			N/A				
	FEOBV striatum (SUVR)	5.972 (0.850)		+0.082 (–0.088 to 0.250), .34, <0.01		N/A		6.220 (0.770)			+0.050 (–0.088 to 0.188), .47, <0.01		N/A		+0.035 (–0.181 to 0.250), .75, <0.01			N/A				
	FEOBV putamen (SUVR)	6.938 (1.043)		+0.108 (–0.105 to 0.319), .32, <0.01		N/A		7.207 (0.905)			+0.067 (–0.097 to 0.231), .42, <0.01		N/A		+0.044 (–0.221 to 0.308), .75, <0.01			N/A				
	FEOBV caudate (SUVR)	5.006 (0.801)		+0.061 (–0.082 to 0.201), .40, <0.01		N/A		5.232 (0.696)			+0.032 (–0.089 to 0.153), .60, <0.01		N/A		+0.029 (–0.156 to 0.213), .76, <0.01			N/A				
	FEOBV posterior cingulate (SUVR)	1.942 (0.192)		–0.002 (–0.031 to 0.027), .89, <0.01		N/A		1.939 (0.205)			+0.010 (–0.022 to 0.041), .55, <0.01		N/A		–0.012 (–0.054 to 0.031), .59, <0.01			N/A				
	FEOBV primary auditory cortex (SUVR)	1.958 (0.317)		+0.005 (–0.049 to 0.059), .86, <0.01		N/A		2.071 (0.284)			+0.026 (–0.021 to 0.073), .28, <0.01		N/A		–0.021 (–0.092 to 0.050), .56, <0.01			N/A				
	FEOBV primary sensorimotor cortex (SUVR)	1.737 (0.227)		–0.002 (–0.032 to 0.027), .88, <0.01		N/A		1.717 (0.201)			+0.012 (–0.015 to 0.040), .38, <0.01		N/A		–0.014 (–0.054 to 0.026), .48, <.01			N/A				
	FEOBV nucleus basalis of Meynert (SUVR)	3.281 (0.568)		+0.086 (–0.026 to 0.197), .14, 0.03		N/A		3.391 (0.453)			+0.044 (–0.064 to 0.153), .42, <0.01		N/A		+0.041 (–0.112 to 0.194), .60, <0.01			N/A				
	NIH EXAMINER^h,i^ (composite *z* score)	–0.10 (1.12)		+0.132 (–0.091 to 0.358), .25, <0.01		+ 0.166 (–0.075 to 0.411), .18, 0.02		0.10 (0.87)			+0.091 (–0.131 to 0.314), .42, <0.01		+0.163 (–0.058 to 0.385), .15, 0.03		+0.049 (–0.264 to 0.364), .76, <0.01			+0.006 (–0.319 to 0.333), .97, <0.01				
	Double Decision train-to-task assessment (*z* score)	–0.19 (1.09)		+1.703 (1.390 to 2.021), <.001^g^, 0.73		+1.666 (1.352 to 1.986), <.001^g^, 0.72		0.19 (0.88)			+0.174 (–0.166 to 0.512), .32, <0.01		+0.486 (0.245 to 0.726), <.01^g^, 0.25		+1.53 (1.073 to 1.989), <.001^g^, 0.32			+1.177 (0.787 to 1.570), <.001^g^, 0.28				
	Freeze Frame train-to-task assessment (*z* score)	–0.22 (0.96)		+1.066 (0.763 to 1.363), <.001^g^, 0.54		+1.179 (0.918 to 1.435), <.001^g^, 0.67		0.21 (1.01)			+0.171 (–0.140 to 0.481), .28, <0.01		+0.294 (–0.002 to 0.585), .05, 0.06		+0.903 (0.477 to 1.327), <.001^g^, 0.16			+0.893 (0.508 to 1.277), <.001^g^, 0.19				

^a^ITT: intent to treat.

^b^Effect sizes are ω^2^ (partial) with effect size<0.01 considered “very small,” 0.01≤effect size<0.06 considered “small,” 0.06≤effect size<0.14 considered “medium,” and effect size≥0.14 considered “large.”

^c^FEOBV: [^18^F]fluoroethoxybenzovesamicol.

^d^SUVR: standard uptake value ratio.

^e^*P*<.05.

^f^N/A: not applicable.

^g^*P*<.05.

^h^NIH EXAMINER: National Institutes of Health The Executive Abilities: Measures and Instruments for Neurobehavioral Evaluation and Research.

^i^Higher scores are better for all measures.

The anterior cingulate cortex is a large, functionally heterogeneous region involved in a range of cognitive functions, including attention, action, and social cognition. We evaluated 3 established subregions: p24c, p24ab, and p32 [94]. The p24c and p24ab subregions share a similar functional profile, particularly in reward-related tasks, with p24c specifically linked to action inhibition. By contrast, p32 is primarily engaged during tasks involving emotion regulation and theory of mind. The p24c subregion showed a significant between-group effect favoring the intervention, indicating that speed training increased cholinergic binding more than the active control (speeded vs nonspeeded SUVR change difference +0.058, 95% CI 0.007-0.110, *P*=.03, small effect size ω²=0.05). A significant within-group effect was observed for the speeded intervention (mean change +0.061, 95% CI 0.019-0.103, *P*=.006, large effect size ω²=0.15) but not for the active control (mean change +0.002, 95% CI –0.028 to 0.033, *P*=.90, effect size ω²<0.01). For the p24ab subregion, a significant within-group effect was observed for the intervention (mean change +0.070, 95% CI 0.020-0.119, *P*=.008, large effect size ω²=0.14) but not for the control group (mean change +0.022, 95% CI –0.030 to 0.073, *P*=.41, effect size ω²<0.01). The p32 subregion showed no significant within- or between-group effects.

### Training Effects on Exploratory Outcomes Measures

Table 2 presents FEOBV-PET SUVRs in exploratory ROIs for the intervention and active control groups at baseline, baseline versus posttest, and baseline versus follow-up. Significant within-group effects were observed for the speeded intervention in brain regions associated with memory, including the hippocampus (mean change +0.089, 95% CI 0.015-0.163, *P*=.02, medium effect size ω²=0.10) and the parahippocampal gyrus (mean change +0.059, 95% CI 0.005-0.114, *P*=.04, medium effect size ω²=0.08). A nonsignificant medium effect was observed for the temporal lobe (mean change +0.043, 95% CI –0.002 to 0.089, *P*=.07, effect size ω²=0.06). Nonsignificant small effect sizes were observed for the global cortex (mean change +0.026, 95% CI –0.007 to 0.059, *P*=.13, effect size ω²=0.03), frontal lobe (mean change +0.033, 95% CI –0.013 to 0.080, *P*=.17, effect size ω²=0.02), occipital lobe (mean change +0.033, 95% CI –0.005 to 0.072, *P*=.10, effect size ω²=0.04), and another region (mean change +0.086, 95% CI –0.026 to 0.197, *P*=.14, effect size ω²=0.03).

There were no significant within-group changes in FEOBV SUVR for the active control across any of the exploratory ROIs. For the speeded intervention, no within-group effects were observed in the parietal lobe, striatum, putamen, caudate, posterior cingulate cortex, primary auditory cortex, or primary sensorimotor cortex. Additionally, no significant between-group effects were detected across the exploratory ROIs.

**Figure 4:**
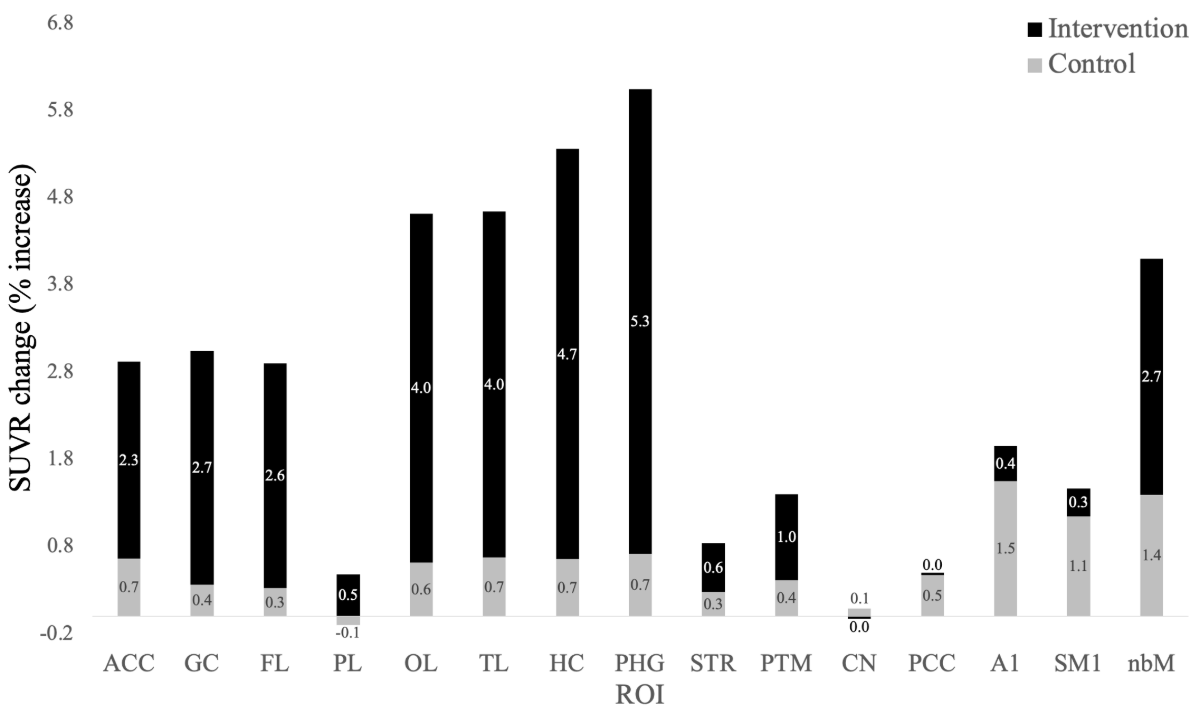
Standard uptake value ratio (SUVR) binding percent increases. Percent change in SUVR binding from baseline to posttest across regions of interest (ROIs) for the intervention and active control groups. Higher scores indicate greater increases in binding. A1: primary auditory cortex; ACC: anterior cingulate cortex; CN: caudate; FL: frontal lobe; GC: global cortex; HC: hippocampus; nbM: nucleus basalis of Meynert; OL: occipital lobe; PCC: posterior cingulate cortex; PHG: parahippocampal gyrus; PL: parietal lobe; PTM: putamen; SMI: primary sensorimotor cortex; STR: striatum; TL: temporal lobe.

Change scores, expressed as percentages, were calculated across the primary and exploratory ROIs for both the intervention and active control groups (Figure 4). The speeded intervention produced a 2.3% increase in FEOBV SUVR in the anterior cingulate cortex (vs 0.7% in the active control), a 4.7% increase in the hippocampus (vs 0.7% in the control), and a 5.3% increase in the parahippocampal gyrus (vs 0.7% in the control).

**Figure 5:**
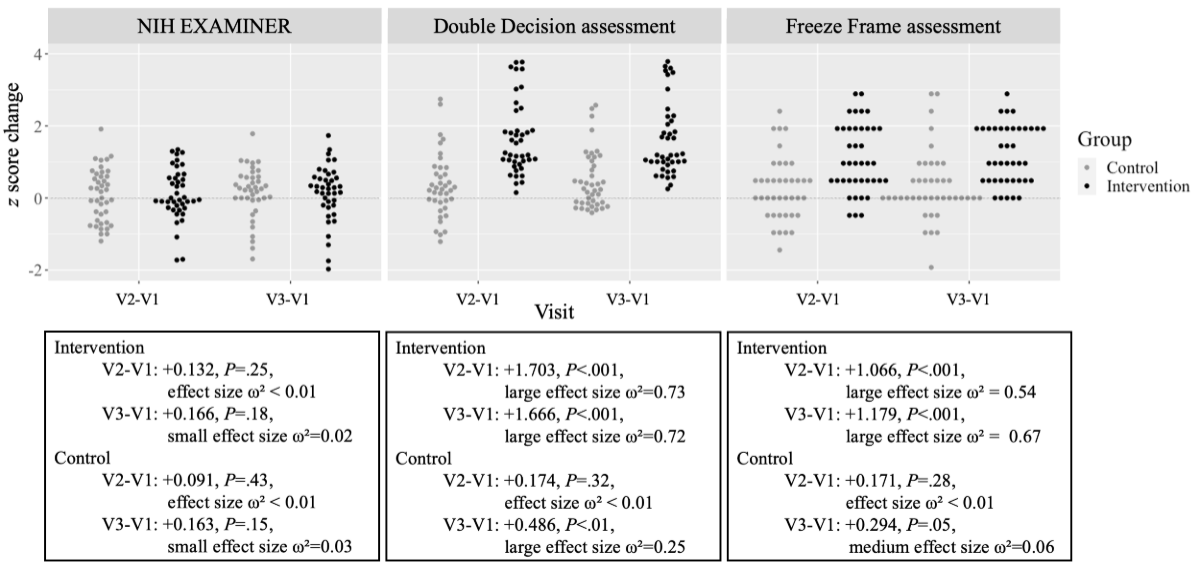
Effects on cognitive measures. Cognitive outcomes (z score change) at posttest (V2) and follow-up (V3) relative to baseline (V1) for National Institutes of Health The Executive Abilities: Measures and Instruments for Neurobehavioral Evaluation and Research (NIH EXAMINER), the cognitive assessment for Double Decision, and the cognitive assessment for Freeze Frame. Each icon represents a single participant’s change score. Higher scores indicate better cognitive performance across all measures. v: visit.

Three exploratory cognitive measures were administered at baseline, posttest, and follow-up (Figure 5). Performance on the cognitive control subtests of the NIH EXAMINER battery (Flanker, Set-Shifting, and Anti-Saccades) showed no significant within- or between-group effects at either posttest or follow-up.

The Double Decision and Freeze Frame assessments, designed to evaluate target engagement, demonstrated significant effects (*P*<.001). Speeded training produced a significant between-group effect on the Double Decision train-to-task assessment (speeded vs nonspeeded *z* score change difference +1.53, 95% CI 1.073-1.989, *P*<.001, large effect size ω²=0.32) and on the Freeze Frame train-to-task assessment (speeded vs nonspeeded *z* score change difference +0.903, 95% CI 0.477-1.327, *P*<.001, large effect size ω²=0.16) at posttest. These effects were maintained at the 3-month no-contact follow-up for both the Double Decision assessment (speeded vs nonspeeded *z* score change difference +1.177, 95% CI 0.787-1.570, *P*<.001, large effect size ω²=0.28) and the Freeze Frame assessment (speeded vs nonspeeded *z* score change difference +0.893, 95% CI 0.508-1.277, *P*<.001, large effect size ω²=0.19). These results confirm that the training effectively engaged the target and that improvements from a brief, intensive training period were sustained after the intervention concluded.

**Figure 6:**
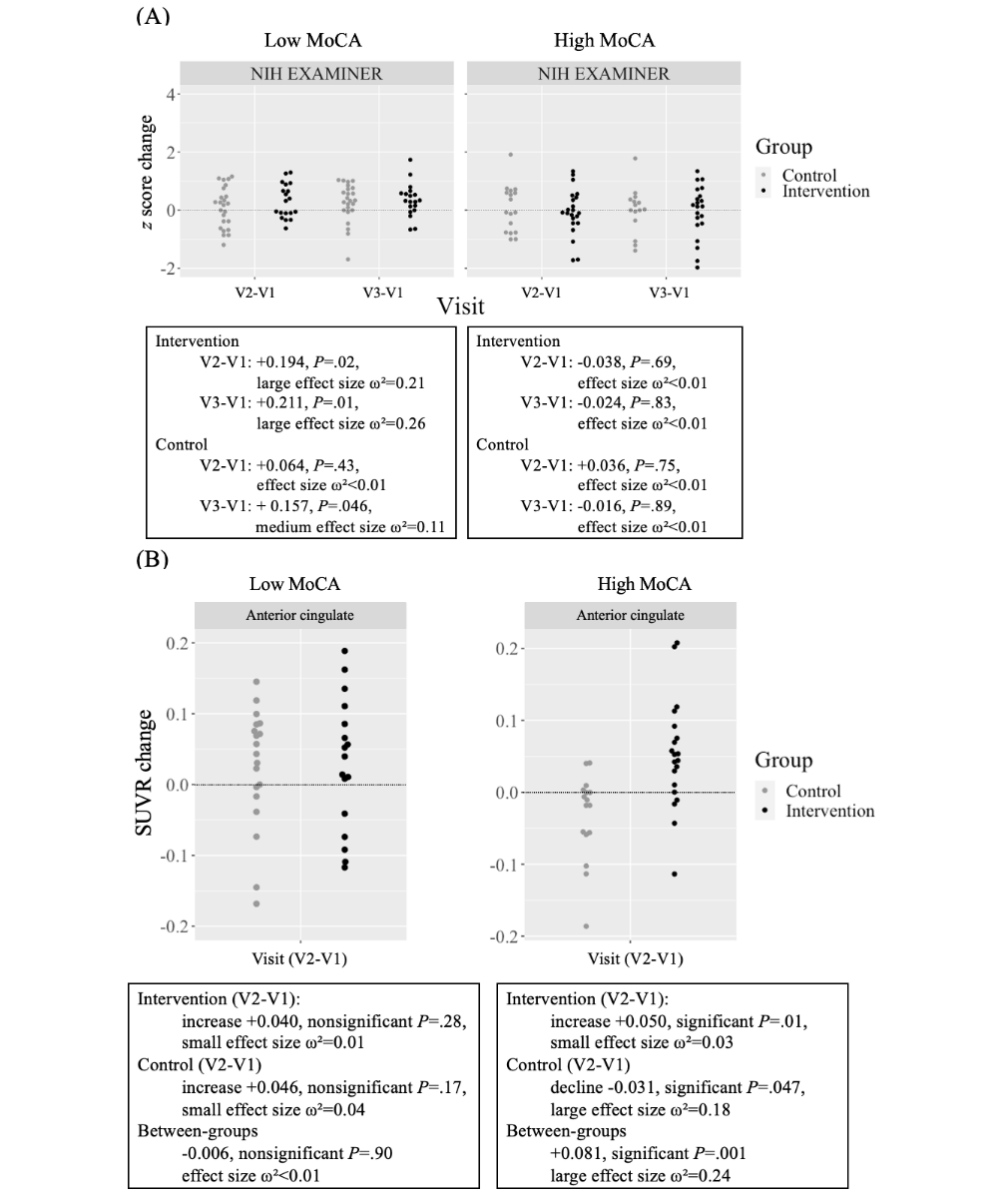
Cognitive outcomes and SUVR binding. (A) Cognitive outcomes (z score change) at posttest (visit 2) and follow-up (visit 3) relative to baseline (visit 1) in participants with low (MoCA 23-26, N=51) and high (MoCA 27-30, N=41) baseline cognition, as measured by the NIH EXAMINER. Each icon represents the change score of a single participant. Higher scores indicate better cognitive performance across all measures. (B) Change in SUVR binding at posttest (visit 2) relative to baseline (visit 1) in the anterior cingulate for the low- and high-baseline cognition subgroups. MoCA: Montreal Cognitive Assessment; NIH EXAMINER: National Institutes of Health The Executive Abilities: Measures and Instruments for Neurobehavioral Evaluation and Research; SUVR: standard uptake value ratio; v: visit.

We explored the effect of baseline cognition using the MoCA, defining “low cognition” as a total score of 23-26 (n=51, 55%, in the ITT) and “high cognition” as a total score of 27-30 (n=41, 45%, in the ITT). At baseline, the low cognition subgroup had a mean NIH EXAMINER executive composite score of 0.09 (SD 0.62), which was notably lower than the mean composite score of 0.65 (SD 0.53) observed in the high cognition subgroup. There was a within-group effect of speed training on the NIH EXAMINER executive composite in the low cognition subgroup (composite *z* score change mean +0.194, 95% CI 0.042-0.351, *P*=.02, large effect size ω²=0.21), but not in the high cognition subgroup (composite *z* score change mean –0.038, 95% CI –0.230 to 0.151, *P*=.69, effect size ω² < 0.01). No within-group effects were observed for the active control in either the low cognition subgroup (composite *z* score change mean +0.064, 95% CI –0.092 to 0.223, *P*=.42, effect size ω²<0.01) or the high cognition subgroup (composite *z* score change mean +0.036, 95% CI –0.187 to 0.259, *P*=.75, effect size ω²<0.01; see Figure 6A).

The effect of speed training was maintained at the 3-month no-contact follow-up, demonstrating sustained cognitive benefits after training (composite *z* score change mean +0.211, 95% CI 0.064-0.365, *P*=.01, large effect size ω²=0.26). The active control also showed a modest improvement relative to baseline at the 3-month follow-up (composite *z* score change mean +0.157, 95% CI 0.009-0.308, *P*=.046, medium effect size ω²=0.11). Given that no significant cognitive improvement was observed immediately posttraining (*P*=.43), this change does not reflect a maintenance effect. It may instead represent either a practice effect on the NIH EXAMINER due to repeated administrations or a delayed cognitive benefit arising from engagement with the control game training.

In the subgroup with high baseline cognition, there was a significant between-group effect favoring the speeded intervention in the primary ROI (speeded vs nonspeeded SUVR change difference +0.081, 95% CI 0.036-0.125, *P*=.001, large effect size ω²=0.24; see Figure 6B). Within-group analyses showed that the intervention group had a significant increase in binding from baseline to posttest (change mean +0.050; 95% CI 0.016-0.082, *P*=.007, small effect size ω²=0.03), whereas the active control group demonstrated a significant decline in binding over the same period (change mean –0.031, 95% CI –0.060 to –0.002, *P*=.047, large effect size ω²=0.18). In the subgroup with low baseline cognition, there were no significant between-group effects (speeded vs nonspeeded SUVR change difference –0.006, 95% CI –0.100 to 0.088, *P*=.90, effect size ω²<0.01). Likewise, there were no significant within-group effects for either the intervention (change mean +0.040, 95% CI –0.032 to 0.112, *P*=.28, small effect size ω²=0.01) or the control group (change mean +0.046, 95% CI –0.019 to 0.109, *P*=.17, small effect size ω²=0.04).

### Adverse Events

Participants reported a total of 54 adverse events, of which 4 (7%) were related to the administration of the radioligand. One participant in the active control group reported 2 mild events (dry mouth and unusual sensations in the mouth), and a second participant who was not randomized reported 2 moderate events (nausea and vomiting). Both participants recovered without treatment. No adverse events were attributed to the training programs, and no serious adverse events or deaths occurred.

## Discussion

### Principal Findings

Age-related declines in neuromodulatory function are closely linked to cognitive decline. In this double-blind, randomized controlled trial, community-dwelling healthy older adults aged 65 and above were assigned to 1 of 2 computerized cognitive training programs: speeded training or nonspeeded training. FEOBV-PET was used to determine whether these training programs could modulate cholinergic function. In the largest FEOBV-PET trial to date, we demonstrate for the first time in humans that speed training significantly increases cholinergic function in the anterior cingulate cortex, our primary ROI. The 2.3% increase in FEOBV SUVR binding observed over the 10-week intervention is comparable in magnitude, but opposite in direction, to the estimated 2.5% decline in anterior cingulate FEOBV SUVR binding [29] per decade of aging.

The speeded intervention showed clear task-based functional associations with the p24c and p24ab subregions of the anterior cingulate. Both exercises involved reward-based tasks performed under conditions of selective attention. One task, in particular, relied heavily on action inhibition, requiring participants to make rapid motor responses to visual foils while withholding responses to specific targets. The p24c and p24ab subregions have been shown to coactivate with elements of the salience and central executive networks, potentially providing a mechanistic explanation for prior findings that speed training enhances functional connectivity within these networks [68]. As expected, no effects were observed in the p32 subregion, consistent with the fact that the training did not involve social theory-of-mind tasks.

The cholinergic system has widespread projections throughout the brain. Exploratory analyses revealed SUVR increases in the hippocampus and parahippocampal gyrus, regions particularly susceptible to age-related declines in cholinergic terminal integrity [95]. These findings suggest potential improvements in memory-related processes and may help explain the memory benefits of speed training reported in previous studies [96]. For example, the well-known ACTIVE trial [97] demonstrated that healthy older adults who completed 10-18 hours of Double Decision training experienced a 29%-48% reduction in dementia risk a decade later [43]. This reduction in dementia risk may reflect the long-term benefits of increased cholinergic function through speed training.

Previous studies using FEOBV-PET have shown that healthy older adults experiencing natural age-related cognitive decline [29], as well as individuals with MCI [30] or dementia [31], share a downregulation of the cholinergic system, albeit to varying degrees. From the perspective of the brain as an information-processing system, this shared neural phenotype suggests that interventions aimed at improving cholinergic neurotransmission could support cognitive health in these populations, regardless of differences in severity or diagnostic classification. Such interventions may help preserve function in healthy aging, slow cognitive decline in MCI, or potentially attenuate progression in dementia. By targeting fundamental neural systems, this approach provides a framework for understanding and addressing cognitive decline across different stages of aging and neurodegeneration.

Our understanding of how computerized cognitive training produces cognitive and functional benefits remains limited. This trial provides direct evidence linking cognitive training to specific changes in brain physiology. Speed training may drive the cognitive and functional improvements observed in the ACTIVE trial [43,97] and other studies in healthy aging [96], MCI [67], and dementia [98,99] by supporting cholinergic network health. This perspective is largely absent from the cognitive training literature, which predominantly emphasizes a neuropsychological framework, focusing on whether training improves specific cognitive functions, such as memory. Incorporating a biological brain health perspective by assessing whether training improves neurological signatures of brain health is essential to align the field of cognitive training with the standards of pharmaceutical drug development.

Improving endogenous cholinergic signaling represents a novel treatment strategy that may offer greater clinical benefits than approaches that rely on exogenously enhancing cholinergic signaling via pharmaceutical therapies. Current treatments for MCI and Alzheimer disease and related dementias include 3 Food and Drug Administration (FDA)–approved cholinesterase inhibitors [100], which increase cholinergic signaling by slowing the breakdown of acetylcholine in and around the synaptic cleft. These drugs provide benefits that are generally modest and temporary at best, and their efficacy may be limited by their mechanism of action: they prolong the presence of acetylcholine in the synapse but do not restore the amount of acetylcholine released during normal signaling.

The present findings demonstrate increased endogenous VAChT binding in cholinergic terminals of the anterior cingulate, hippocampus, and parahippocampal gyrus following speed training. While VAChT binding does not directly measure acetylcholine concentrations in the synaptic cleft, it indicates an enhanced presynaptic capacity for acetylcholine packaging and release. Several mechanisms may underlie this effect. One potential mechanism is activity-dependent upregulation of VAChT driven by increased neuronal firing and cholinergic demand, consistent with preclinical evidence that heightened cholinergic activity enhances VAChT expression [101]. Another is the induction of trophic signaling: cognitive stimulation in both animal models and humans increases neurotrophic factors such as nerve growth factor [102-108] and brain-derived neurotrophic factor [109-111], which support cholinergic neuron health and can upregulate choline acetyltransferase and VAChT expression. These adaptive changes likely reflect remodeling of existing synapses, including improved vesicle mobilization and transporter availability, rather than synaptogenesis [112-114]. Regardless of the precise biological mechanism underlying the observed increase in binding, these results align with animal studies showing region-specific changes detected by FEOBV-PET, which were subsequently validated through immunohistochemistry following training [75]. Collectively, these findings suggest that cognitive training increases cholinergic signaling capacity, counteracting age-related decline by restoring presynaptic efficiency in circuits supporting attention, memory, and executive function.

The between-group effect size observed in the p24c subregion of the anterior cingulate is consistent with effect sizes reported for cognitive and functional outcomes in meta-analyses. For instance, Basak et al [115] (215 studies) reported benefits in healthy aging (near transfer *g*=0.38, far transfer *g*=0.22) and in MCI (near *g*=0.27, far *g*=0.18), aligning with prior meta-analyses by Mewborn et al [116] (97 studies; near transfer *g*=0.44, far transfer *g*=0.15) and Lampit et al [117] (52 studies; near transfer *g*=0.22). In patients with dementia, Bahar-Fuchs et al [118] (33 studies) reported significant near transfer benefits (*g*=0.84), as did Hill et al [119] (17 studies; near transfer *g*=0.26). Organizations such as NASEM [40], WHO [42], AAN [41], and the Alzheimer’s Association [120] have begun to incorporate cognitive training into discussions of brain health, based on meta-analytic evidence in healthy aging, MCI, and dementia [121,122]. Furthermore, given the unmet need for effective interventions across clinical populations, the FDA has indicated that effect sizes of 0.20-0.30 (Cohen *d*) for a low-risk cognitive training program may provide sufficient efficacy signals to support approval [123]. The speed training intervention used in the ACTIVE trial demonstrated effect sizes within this range, with subsequent analyses showing clinically relevant reductions in dementia risk. In this context, the between-group effect size observed in our study can be considered clinically meaningful.

Exploratory subgroup analyses based on baseline MoCA scores showed that the lack of a within-group effect on NIH EXAMINER (contrary to previous findings [91]) was attributable to differences in baseline cognition. Participants with low baseline cognition demonstrated significant improvements on the cognitive control subtests of NIH EXAMINER (Flanker, Set-Shifting, and Anti-Saccades) following speed training, and these gains were maintained at follow-up. However, the smaller size of this subgroup likely limited statistical power to detect a between-group effect. By contrast, participants with high baseline cognition exhibited a ceiling effect, limiting the potential for measurable improvement on these subtests. These specific tasks may be less sensitive to change in high-functioning older adults [90]. While the low cognition group demonstrated a cognitive benefit, the high cognition group showed between-group differences in FEOBV SUVR within the primary ROI, favoring the speed training intervention. Previous research has documented distinct patterns of FEOBV-PET SUVR decline across healthy and clinical populations, with the most pronounced reductions in healthy adults observed in the anterior cingulate, striatum, and primary sensorimotor cortex. By contrast, individuals with MCI exhibit the greatest declines in the global cortex, occipital lobe, and parietal lobe, whereas those with dementia show the most pronounced reductions in the temporal cortex [29-31,34,35]. The distinct pattern of cholinergic changes observed across the primary and exploratory ROIs between the low and high cognition subgroups aligns with these prior findings. Although further research is needed to fully elucidate this dissociation, we interpret the cognitive gains in the low cognition group and the SUVR changes in the high cognition group as reflecting regional differences in cerebral SUVR topography between these subpopulations, as well as the limited sensitivity of the 3 selected NIH EXAMINER subtests. To evaluate the relationship between changes in FEOBV-PET SUVRs and cognitive performance, future studies should employ a validated, comprehensive cognitive assessment battery (with measures of processing speed, attention, memory, and executive function) and recruit healthy older adults within a narrow high-MoCA range (27-30) when designating the anterior cingulate as the primary ROI.

### Strengths

This is the largest FEOBV-PET study to date, conducted within a rigorously designed randomized clinical trial, featuring a well-matched active control and an a priori statistical analysis plan with prespecified ROIs.

In vivo FEOBV-PET provides a novel biomarker for assessing cholinergic function. Our trial not only replicates findings from smaller pilot studies demonstrating that FEOBV-PET can safely localize cholinergic terminals in humans, but also shows that terminal densities can change following a 10-week cognitive training intervention. As an imaging tool specific to neuromodulatory function, FEOBV-PET offers a way to quantify cholinergic status across disease states and may ultimately serve as a valuable method to monitor treatment response.

### Limitations

The limited racial and ethnic diversity of trial participants constrains the generalizability of these findings to non-White populations. To address this, we implemented extensive outreach initiatives over the course of the trial, including contacting 20 community centers in Montreal with greater representation, more than 200 churches, and 10 local libraries, as well as participating in multiple media interviews about INHANCE. Despite these and other efforts, most participants identified as White. Barriers to minority representation in clinical trials are well documented and may include mistrust stemming from historical research abuses, time and resource constraints, cultural and language differences, discomfort with the research process, and perceptions of increased personal risk. In the case of INHANCE, the requirement to undergo radioligand imaging and commit to 35 hours of training may have further discouraged participation [124-127]. Future studies should aim to reduce participant burden and implement more equitable research practices [128].

This mechanism paper was motivated by the positive cognitive and functional benefits observed in healthy older adults randomized to speed training in the ACTIVE trial. Although we intended to recruit only healthy older adults, the relatively low MoCA cut-off of 23 [84] may have inadvertently included participants with undiagnosed MCI, thereby introducing additional variance into our trial.

Finally, only known confounders of cognitive training reported in the literature (eg, major depression) were specified in the published protocol. Potential confounders of cholinergic plasticity, such as smoking or systemic estrogen use through hormone replacement therapy, were not accounted for in this study.

### Future Directions

Future pivotal trials should evaluate whether speed training increases cholinergic binding in clinically diagnosed MCI populations and determine if FEOBV-PET–measured reductions in binding can predict progression from MCI to dementia, as well as whether this transition can be favorably modified by improving neurotransmission through speed training.

### Conclusions

Identifying and implementing effective strategies to support brain health has the potential to reduce health care costs, increase workforce participation and community engagement, and improve quality of life. As neuromodulatory centers of the brain play a causal role in cognitive performance, improving cholinergic signaling may help protect against age-related cognitive decline. This trial demonstrates that an intensive, time-limited period of speed training can enhance neuromodulatory health in community-dwelling adults aged 65 and older, specifically in regions that support selective attention, inhibitory control, and memory. These findings support the use of this promising, low-risk intervention and contribute to a mechanistic understanding of cognitive training that establishes a foundation for future trials.

## Appendix

Multimedia Appendix 1 Participant consent forms in English and French.

Multimedia Appendix 2 Positron emission tomography and magnetic resonance imaging acquisition and data analysis.

Multimedia Appendix 3 Participant-facing questionnaires in English and French.

Multimedia Appendix 4 Study flyers in English and French.

Multimedia Appendix 5 CONSORT-eHEALTH checklist (V 1.6.1).

## Abbreviations

AAN: American Academy of Neurology
ACTIVE: Advanced Cognitive Training for Independent and Vital Elderly
CONSORT: Consolidated Standards of Reporting Trials
FDA: Food and Drug Administration
FEOBV: [^18^F]fluoroethoxybenzovesamicol
GDS-SF: Geriatric Depression Scale—Short Form
INHANCE: Improving Neurological Health in Aging via Neuroplasticity-based Computerized Exercise
ITT: intent to treat
LORIS: Longitudinal Online Research and Imaging System
MCI: mild cognitive impairment
MoCA: Montreal Cognitive Assessment
MRI: magnetic resonance imaging
NASEM: National Academies of Sciences, Engineering, and Medicine
NIH EXAMINER: National Institutes of Health The Executive Abilities: Measures and Instruments for Neurobehavioral Evaluation and Research
PET: positron emission tomography
ROI: region of interest
SUVR: standard uptake value ratio
VAChT: vesicular acetylcholine transporter
WHO: World Health Organization

## Data Availability

Beginning May 31, 2026, deidentified participant data from INHANCE will be made available to verified academic and industry researchers. Investigators will be able to conduct both confirmatory and exploratory analyses using the Longitudinal Online Research and Imaging System (LORIS), a freely accessible, open-source data archive with provenance-sharing features. The dataset will be accessible online [129].
