# Supplementary material for: Effects of Computerized Cognitive Training on Vesicular Acetylcholine Transporter Levels using [18F]Fluoroethoxybenzovesamicol Positron Emission Tomography in Healthy Older Adults: Results from the Improving Neurological Health in Aging via Neuroplasticity-based Computerized Exercise (INHANCE) Randomized Clinical Trial
Source: JMIR Serious Games. 2025 Oct 13;13:e75161. doi: 10.2196/75161 (PMC12559824; doi:10.2196/75161)
Supplement: Multimedia Appendix 1 [file games_v13i1e75161_app1.pdf]

Multimedia Appendix 1: Participant consent forms in English and French

neuro
Institut-Hôpital neurologique de Montréal
Montreal Neurological Institute-Hospital

INFORMATION AND CONSENT FORM

**Research Study Title:**	Improving Neurological Health in Aging via Neuroplasticity-based Computerized Exercise (INHANCE)
**Protocol number:**	PSC-0903-19
**Researcher responsible for the research study:**	Etienne de Villers-Sidani, MD, FRCP(C)
**Co-Investigator(s)/sites:**	Jean-Paul Soucy, MD, MSc;<br>Thomas Van Vleet, Ph.D.<br>Mouna Attarha, Ph.D.
**Sponsor:**	Posit Science Corporation (PSC)<br>funded exclusively by the National Institute of Aging

INTRODUCTION

We are inviting you to take part in this research study because you are a person aged 65 years or older in generally good health, and we think you may be eligible to partake in our study to evaluate the effects of cognitive training programs (computerized programs) on the brain.

However, before you accept to take part in this study and sign this information and consent form, please take the time to read, understand and carefully examine the following information. You may also want to discuss this study with your family doctor, a family member or a close friend.

We invite you to speak to the researcher responsible for this study ("the researcher") or to other members of the research team and ask them any questions you may have about this study. Please also ask a member of the research team about any parts of this consent form you do not understand.

BACKGROUND

Americans and Canadians are living progressively longer lifespans; however, the onset of dementia does

not occur later just because people are living longer. Certain non-drug therapies have been shown to maintain brain health during older age. Most notably, forms of progressive computerized brain training (computer programs) that target specific areas and processes in the brain that affect one's focus and alertness have helped maintain brain health.

PURPOSE OF THE RESEARCH STUDY

The purpose of this study is to validate and evaluate how well two computerized cognitive training programs work on improving neurological and neuropsychological health in older adults.

We will look at whether the cognitive training has had any impact or change on the systems in your brain that deal with alertness and focus by taking PET images and MRI scans of your brain before and after your training period (explained in further detail later).

For this research study, we will recruit approximately 108 healthy participants, to ensure the successful completion of 90 participants. Participants will be men and women, aged 65 years and older.

DESCRIPTION OF THE RESEARCH PROCEDURES

This research study will take place at Montreal Neurological Institute-Hospital.

I. Duration and number of visits

Your participation in this research study will last about 6 months and will include 4-5 visits to the clinic. During your participation, you will engage in assigned training exercises for 7 sessions per week over 10 weeks with each session taking approximately 30 minutes to complete.

II. Overview of study participation

There are 6 periods to this study:

1. Screening Visit (V0)
- You will undergo a few screening assessments to determine if you qualify to participate in the study.
- You will come into the clinic for 1 visit.
- This visit will last approximately 1 hour.

2. Baseline Visit (V1):
- You will undergo additional procedures (the cognitive assessment, the PET scan and MRI scan)
- This period will last up to 4 weeks.
- You will come into the clinic for 1 visit, which may be split into 2 sessions, if needed.
- This visit will last approximately 3 hours.

3. Program Orientation and Intervention period

Before you start your training, we will invite you in for a program orientation session so that study staff may familiarize you to the training program in person.

We will also provide you with detailed, written information on how to perform the training program over the next several weeks.

- You will be randomly selected (like a flip of a coin) to participate in one of two groups for the computerized training programs and begin your daily training assessments for the next 10 weeks.
- This period lasts 10 weeks.
- You will come into the clinic for 1 visit, the program orientation visit. This visit may also be performed remotely through a phone or video call that the study staff will set up for you. The study staff will then follow-up with you during weekly check-ins to answer any questions and troubleshoot any issues. These check-ins will be conducted via email.
- This visit will last 1 hour.

4. Post-Intervention Visit (V2):
- You will have finished your 10 weeks of program training and come into the clinic for post-intervention assessments.
- You will no longer have access to the intervention applications after this visit.
- This visit will last approximately 3 hours and may be split into 2 sessions, if needed.

5. No-Contact Period
- Following the Post-Intervention Visit, you will be entered into a follow-up period lasting 3 months with no further program use. The study team will not be in contact with you other than to confirm the follow-up visit.

6. Follow-Up Visit (End of Study, V3)
- After the three months with no contact, we will invite you in for a final visit where you will complete some a final cognitive assessment.
- This visit will take place 6 months after your initial enrollment.
- This visit will last approximately 1.5 hours.

COMPUTERIZED TRAINING PROGRAMS

This study employs two computerized interventions: speed and attention training and executive function training. You will be assigned to one of the following groups:

Group 1: will do the speed and attention computer program training.
Group 2: will do the executive function computer program training.

This study is randomized, which means that you will be assigned to one of the groups. You may not choose the group to which you will be assigned; this process is done randomly, like flipping a coin. One person out of two (50%) will do the speed and attention computer program training, and one person out of two (50%) will do the executive function computer program training.

You will be required to participate in the assigned training exercises each day for 7 sessions per week over 10 weeks with each session taking approximately 30 minutes to complete.

Both can be done on any computer with an internet connection, and therefore within the comfort of your own home. If required, we can lend you a laptop computer or electronic tablet. You will be given the

opportunity to ask any questions about the computer program to ensure your proper understanding of how to use the program.

We will ask and remind you ***not*** to discuss details related to their training program with study staff, colleagues, friends, or acquaintances.

The following are some details to consider regarding the use of the program:

Location of Use: you may use the program at you place of residence or in clinic. We will offer you a loaner mobile device (tablet) from which you can use the program. You can otherwise opt to train on your personal device.

Fatigue: We expect that some participants may not be able to complete a full session which takes approximately 30 minutes to complete in one sitting. To accommodate this issue, participants may choose to break the time into shorter segments, such as 15 min in the morning and 15 min later in the day. Participants can pause the sessions to take a break at any time and continue where they left off.

You may discuss this with the study staff, who will work out a schedule that is most suitable for you. If ever you need to reduce the number of sessions per week, the study staff will work with you to generate a training schedule that works for you.

III. Study Procedures

During your participation in this research study, you will participate in the following procedures:

DESCRIPTION OF STUDY PROCEDURES	
**Procedure**	**Description**
Medical history and demographics	We will collect your complete medical history, including any medications you have used or are currently using and any other therapies or procedures you have had. Throughout the study you will be asked to report if you think that anything changes with your health and if anything has changed such as any new medications that you may have taken.<br><br>We will also ask you about age, your race and ethnic background.
Questionnaires and assessments	We will assess your cognitive status using the Montreal Cognitive Assessment (MoCA).<br><br>We will ask you some questions regarding the state of your mental health with the Geriatric Depression Scale (GDS) – Short Form and the Columbia-Suicide Severity Rating Scale (C-SSRS).
Neuropsychological assessments	A member of the study team will perform a neuropsychological assessment to measure specific age-related cognitive decline.

PET Scan and FEOBV radiotracer	PET is a nuclear medicine scanning procedure that involves the administration of very small amounts (tracer dose) of a chemical that will allow us to view specific areas of your brain (the FEOBV tracer). When administered intravenously, this chemical circulates in the blood to reach its target where it will briefly stay, before it decays. **The FEOBV radiotracer being used in this study is experimental, which means it has not been approved by Health Canada for use in regular medical practice.** However, Health Canada does not object to its use in this study.<br><br>This is a chemical tracer that is labeled with an atom which allows for it to be detected on the PET camera and provide a clearly defined image of your specific areas of your brain.<br><br>During this process, the tracer will emit a very small amount of radioactivity that can be detected by a sophisticated PET camera. With the help of high-power computing, researchers are then able to study the distribution within brain of the chemical that has been administered. **No effect of the chemical can be detected in a given individual, since it is always administered in very small amounts (tracer dose).**<br><br>All PET imaging sessions scheduled as part of your participation will be supervised by a qualified nuclear medicine technician. A physician is available, if needed. On your arrival at the MNI PET unit, you will have to fill in routine questionnaires about your general physical condition. Then, a fine needle-catheter will be inserted into an arm vein. This catheter will be used for the administration of FEOBV.<br><br>You will then have to wait for approximately 180 minutes for the chemical to be appropriately distributed in your brain. During this time, you should remain at rest, but will be able to use the washroom and walk around if necessary.<br><br>You will then be asked to lie down on a bed that will be moved into a cylindrical opening for the scanning process, which lasts approximately 40 minutes.<br><br>The device is completely passive and has no electrical (or other) output that may be harmful.<br><br>The scans will be completed at the MNI PET Unit, or at the Concordia PERFORM Centre (7200 Sherbrooke St. W., Montreal, QC, Canada, H4B 1R2), depending on the availability of the facility. The study team will let you know where your scans will occur.

	If your scan is conducted at the PERFORM Centre, following the administration of FEOBV at the MNI, you will be transferred to the PERFORM Centre. The study team can organise transport (taxi), or you may drive yourself.
MRI	This is a test that uses a magnet and radio waves to produce a detailed scan (picture) of the brain and spine. An MRI scanner looks like a large doughnut-shaped magnet that often has a tunnel in the center. You will be asked to lie down on a table that slides into the tunnel. Your head will be supported by pillows. Please let the study staff know if you get uncomfortable or frightened when in small spaces.<br><br>Please let the study staff know if you have any implanted devices.<br><br>Inside the scanner you will hear a fan and feel air moving. You may also hear tapping or snapping noises as the scans are taken. You will be given earplugs or headphones with music to reduce the noise. It is very important to hold as still as possible while the scan is being done. You may be asked to hold your breath for short periods of time.<br><br>You will be provided with a call button so that you can communicate with the MRI team at any time and you will be able to talk to the team via an intercom between scans.<br><br>Your study staff will give you detailed instructions on how to prepare for your scans. You will need to remove all metal from your body and change into scrubs.<br><br>This scan may take up to 20 minutes.
Behavioral Assessment	To assess for acetylcholinergic function (how your muscles respond), we will measure your heart rate variability and/or pupillometry (the widening of your pupils) during train-to-task assessments or as needed.<br><br>The pupillometry acquisition will be carried out using Tobii Pro Glasses 2. These are glasses that you will have to wear; in doing so, they will monitor your eye movements.<br><br>The heart rate variability will be acquired using a wearable wrist band monitor.
Email communication with study staff	This study will rely on the use of email communication between study staff and research participants as part of their participation in the clinical trial.

	Study staff are expected to email participants about their upcoming appointments, provide weekly updates on program usage, or communicate other important study information, including the instructions for completing study activities remotely.<br><br>Participants may also ask questions of study staff using email.
Secure mobile app usage	We are aware that mobile app use can pose some confidentiality hazards to users and hence put in place several important safeguards to minimize potential risks.<br><br>Security of electronic data is ensured at the level of the server, the user, and the database.<br><br>We will provide you with de-identified log-in to access the training program. The program will not capture, collect, transmit or store personally identifiable data, except for dates that training exercises are completed. The computerized program also does not include geographic location data.

PARTICIPANT'S RESPONSIBILITIES

- Attend all study visits.
- Complete one training session each day, each session will take approximately 30 minutes to complete.
  - Taking a pause or breaking up the session throughout the day is permitted.
- Check your emails and respond to the coordinator 'check-in' messages.
- Follow the instructions of the study investigator and staff.
- Please do not discuss the details of the computerized training program with anyone.

BENEFITS ASSOCIATED WITH THE RESEARCH STUDY

There is no direct benefit to you for participating in this research. However, we hope that the study results will contribute to the advancement of scientific knowledge in the study field.

RISKS ASSOCIATED WITH THE RESEARCH STUDY

A possible risk associated with this study is a breach of confidentiality or use of your personal information by a third party. To limit this risk, we will take the steps to protect your confidentiality described in the Confidentiality section, below.

<u>Discomfort During Assessments and Training.</u>
Computerized assessments and training may be fatiguing or frustrating for some individuals. To minimize this potential discomfort, breaks are encouraged and scheduled within the session. You may discontinue testing sessions at any time if you fee to be under undue strain.

Lack of Assessment Feedback.
Participation in this study does not include feedback to participants on their individual assessment results, which may be frustrating.

Risks of Email Communication.
There are risks associated with email communication, and these risks increase when emails are sent without an encryption service. Risks of sending or receiving unencrypted emails include, but are not limited to:

- Others can intercept messages.
- If messages are sent or received on an employer-owned device, the employer may have the right to save and read the messages. The internet or cell-phone provider may also have the right to save and read email messages.
- A copy of the message may be saved on a device or computer system, even if it is deleted.
- If an email address is not typed correctly, it can be sent to the wrong person.
- Emails can spread computer viruses.
- Others may be able to access messages on devices that were lost, stolen, or thrown away.
- If a user changes emails without notifying study staff, they may miss communications.

Loss of Privacy
One of the risks to the participants are those that would follow a breach of confidentiality and the disclosure of clinical information. Participation in any research study, including this one, may involve a loss of privacy. Procedures designed to maintain data confidentiality include (1) formal protocol training sessions for all study staff members emphasizing the importance of confidentiality, (2) adherence to specific procedures developed to protect participants' confidentiality, and (3) formal mechanisms limiting access to information that can link data to individual participants.

PET scan
During the PET imaging sessions, participants may feel a stinging sensation at the time of the catheter insertion into the vein. As with any other type of injection, there is a minimal chance of infection on the site of injection. To limit this risk, sterile equipment will be used on a thoroughly disinfected skin area. Moreover, the prolonged immobility on the couch may also be a source of restlessness and discomfort for some participants. PET imaging involves the injection of specific agents (FEOBV) not normally present in the human body. Like any other chemical or pharmaceutical compound, these agents have a potential to produce undesirable or allergic reactions. However, such reactions have never been observed with the doses to be used in this study.

Given that FEOBV is a radioactive compound, this means that participants will be exposed to a small dose of radiation (measured in milliSieverts, or mSv), above what one individual is usually exposed to in daily life (natural radiation in the environment, cosmic rays, etc.), or for medical reasons (diagnostic X-rays, radiation therapy, etc.). Most of the radioactivity will be gone from the body after a few hours (by 20 hours, it will be essentially undetectable). The risk which is alluded to when discussing risk associated with radiation exposures of the level seen in PET scanning is that of developing a cancer at some point in the future, which would not have developed otherwise. As a general concept, it is known that radiation increases the risk of developing cancer over certain doses. However, because of the very small doses used for PET imaging, this has never been observed. The risk is therefore low. The FEOBV radiotracer being used in this study is experimental, which means it has not been approved by Health Canada for use in regular medical practice. However, Health Canada does not object to its use in this study. **Nationally**

accepted limits of radiation doses administered for research purposes have been defined at 50 mSv per year, so you need to inform the study team of all the scans you have undergone in the last 12 months, so as not to exceed the limit.

You are also required to mention your participation in this study to any investigator asking you to take part in a study involving radiation. The dose you are expected to receive for the 2 PET scans as part of the study is estimated at 11-15.4 mSv.

Risks specific to FEOBV:
No adverse reactions to FEOBV have been reported yet. However, there are some potential risks as listed below:

Risks of FEOBV for pregnant women, unborn children or to children of breastfeeding women are not known, therefore FEOBV should be avoided in pregnant and breastfeeding women. Consequently, pregnant and breastfeeding women may not participate in this study.

Given that FEOBV is a radioactive compound, traces of the small amount participants will be exposed to can appear in urine and fecal matter and risk contaminating of the surfaces it touches. For up to 12 hours after the administration of FEOBV, a toilet should be used instead of a urinal, and the toilet must be flushed several times after use.

MRI
During this test, you will be exposed to a strong magnetic field and radio waves. However, no long-term negative side effects have been observed for this type of exam.

An MRI scan can be a rather noisy procedure, and some people may feel uncomfortable while lying in the scanner. You will be given ear plugs to block out the noise.

You may briefly experience claustrophobia (a closed-in feeling) during the MRI procedure. Some people may find the combination of the noise and the feeling of being "closed in" uncomfortable. You will be in constant communication with the MRI technician, and you will be provided with a "call button" if you do not feel well and want the procedure to stop.

Please inform the study staff if you have one of the following contraindications:
- Pacemaker
- Aneurysm clip
- Heart/vascular clip
- Prosthetic valve
- Metal prosthesis
- Pregnancy or intend to become pregnant
- Metal fragments in body
- Transdermal patches (must be removed prior to exam, please bring an additional patch to apply after the scan)

INCONVENIENCES LINKED TO STUDY PROCEDURES

These are the only foreseeable inconveniences that may result from study participation:

- Time commitment required to complete the trainings daily.

VOLUNTARY PARTICIPATION AND THE RIGHT TO WITHDRAW

Your participation in this study is voluntary. Therefore, you may refuse to participate. You may also withdraw from the *ongoing* project at any time, without giving any reason, by informing a member of the study team. Your decision not to participate in the study, or to withdraw from it, will have no impact on the quality of care and services to which you are otherwise entitled. You will be informed in a timely manner if any information becomes available that may impact your willingness to continue participating in this study.

The researcher or the Research Ethics Board may put an end to your participation without your consent. This may happen if new findings or information indicate that participation is no longer in your interest, if you do not follow study instructions, or if there are administrative reasons to terminate the project.

If you withdraw or are withdrawn from the study, you may also request that the data already collected about you be removed from the study.

You may choose to stop using the computer program but continue to partake in the study and come in for a *Post-Intervention Visit (V2)* and *Follow-up Visit (V3).*

Any new findings that could influence your decision to stay in the research project will be shared with you as soon as possible.

CONFIDENTIALITY

During your participation in this study, the researcher and his/her team will collect and record information about you. They will only collect information necessary for the study.

The following information may be collected: information from your medical chart, including your identity, concerning your past and present state of health, your lifestyle, as well as the results of the tests, exams, and procedures that you will undergo during this research project. Your research file could also contain other information, such as your name, sex, age, date of birth, ethnic origin and other dates from data collected (such as dates for completion of assessments and training exercises).

All the information collected during the research project will remain confidential to the extent provided by law. You will only be identified by a code number. The key to the code linking your name to your study participant number will be kept by the researcher.

To ensure your safety, a confirmation of participation in clinical research form will be placed in your medical chart. As a result, any person or company to whom you give access to your medical chart will have access to this information.

The researcher may forward your coded data to the sponsor. However, once coded data is transferred outside of Canada, protections equivalent to those in Canada and Quebec will be enforced.

The study data will be stored for 25 years by the researcher responsible for the study.

The data may be published or shared during scientific meetings; however, precautions will be taken to ensure that it will not be possible to identify you.

For auditing purposes, the research study files which could include documents that may identify you may be examined by a person mandated by the study sponsor, the institution, or the Research Ethics Board and the NIH. All these individuals and organizations adhere to policies on confidentiality.

Data Archive

Two years after the completion of the trial, we will store all of the coded study data, including MRI and PET data, from all the participants in the study on a platform that has been vetted and approved by the institution. To protect your confidentiality, imaging data will be defaced, which means your face in the images will be masked.

Storing all the study information in a database for this trial will allow other academic researchers who were not affiliated with the trial to conduct further analyses on the research results. This will only happen two years after the study is over so that the objectives of the main study are all analyzed before other non-affiliated researchers have access to the data.

Sharing the data on an institutionally vetted database is open; this means any researcher can access the study data either freely or after registering with the platform. However, as mentioned in the Confidentiality section, any data that may be published or shared will have the precautions in place to ensure your identity is kept confidential.

INCIDENTAL FINDINGS

Material incidental findings are unexpected findings made in the course of the study that may have significant impacts on your current or future wellbeing or that of your family members. A material incidental finding concerning you in the course of this research will be communicated to you and to a health professional of your choice.

FUNDING OF THE RESEARCH PROJECT

The researcher and the institution have received funding from the National Institute of Aging to conduct this research project.

MARKETING POSSIBILITIES

The computerized training programs being studied are developed by Posit Science Corporation. The Principal Investigators at Posit Science Corporation may benefit from commercial activities that may later derive from the results of this research. However, you will not receive any financial benefits.

CONFLICT OF INTERESTS

The Principal Investigators are employees of and hold stock in Posit Science Corporation. The Principal Investigators at Posit Science Corporation may benefit from commercial activities that may later derive from the results of this research.

COMPENSATION

You will receive compensation for costs and inconveniences incurred during this research study. If you withdraw from the study, or are withdrawn before it is completed, you will receive compensation proportional to the number of visits you have completed.

The breakdown of reimbursement is as follows:

- Baseline Visit including PET imaging (V1): $40
- You will receive $13 for every 10 training session completed (maximum of $91 for completing all 70 sessions) during the intervention period.
- Post-intervention visit including PET imaging (V2): $40
- Follow-up visit (V3): $40

Participants who complete the study in its entirety will be reimbursed $211

Participants who complete the PET imaging at the Concordia PERFORM Centre will receive an additional 50$ for transportation reimbursement at these visits.

For participants who are loaned a tablet, compensation for all study activities completed through the end of the *Intervention Period* will occur after the participant returns the loaned tablet to the study staff at the *Post-Intervention Visit* (V2). Participants who withdraw from the study will be compensated for all study visits they have completed once the loaned tablet has been returned to the study staff.

SHOULD YOU SUFFER ANY HARM

Should you suffer harm of any kind following any procedure related to the research study, you will receive the appropriate care and services required by your state of health.

By agreeing to participate in this research project, you are not waiving any of your legal rights nor discharging the researcher, the sponsor or the institution, of their civil and professional responsibilities.

CONTACT INFORMATION

If you have questions or if you have a problem you think may be related to your participation in this research study, or if you would like to withdraw, you may communicate with the researcher or with someone on the research team at the following number: 514-398-2964

For any question concerning your rights as a research participant taking part in this study, or if you have comments, or wish to file a complaint, you may communicate with the Montreal Neurological Hospital Patient Ombudsman at the following phone number: (514) 934-1934, ext 48306.

OVERVIEW OF ETHICAL ASPECTS OF THE RESEARCH

The McGill University Health Centre Research Ethics Board reviewed this research and is responsible for its ongoing ethical oversight.

Research Study Title: Improving Neurological Health in Aging via Neuroplasticity-based Computerized Exercise (INHANCE)

SIGNATURES

Signature of the participant

I have reviewed the information and consent form. Both the research study and the information and consent form were explained to me. My questions were answered, and I was given sufficient time to make a decision. After reflection, I consent to participate in this research study in accordance with the conditions stated above.

I authorize the study team to have access to my medical record for the purposes of this study.

I authorize a member of the research study to contact me in the future to ask if I am interested in participating in other research.

Yes ☐ No ☐ If yes, please provide contact information: ________________________

____

Name of participant Signature Date

Signature of the person obtaining consent

I have explained the research study and the terms of this information and consent form to the research participant, and I answered all his/her questions.

____

Name of the person obtaining consent Signature Date

Institut-Hôpital neurologique de Montréal
Montreal Neurological Institute-Hospital

FORMULAIRE D'INFORMATION ET DE CONSENTEMENT

**Titre de l'étude de recherche :**	Améliorer la santé neurologique du vieillissement grâce à un exercice informatisé basé sur la neuroplasticité
**Numéro de protocole :**	PSC-0903-19
**Médecin responsable de l'étude de recherche :**	Étienne de Villers-Sidani, M. D., FRCPC
**Co-investigateur(s)/sites :**	Jean-Paul Soucy, M. D., MSc<br>Thomas Van Vleet, D^{r}<br>Mouna Attarha, D^{re}
**Commanditaire :**	Posit Science Corporation (PSC)<br>financé en exclusivité par le National Institute on Aging

INTRODUCTION

Nous vous invitons à participer à la présente étude de recherche, car vous avez 65 ans ou plus, êtes globalement en bonne santé et pourriez être admissible à notre étude évaluant les effets des programmes d'entraînement cognitif (programmes informatisés) sur le cerveau.

Toutefois, avant d'accepter de participer à l'étude en signant le présent formulaire d'information et de consentement, veuillez lire, examiner attentivement et comprendre les renseignements suivants. Nous vous recommandons par ailleurs de discuter de la présente étude avec votre médecin de famille, un membre de votre famille ou un ami proche.

Si vous avez des questions sur la présente étude, veuillez les poser au médecin responsable de l'étude (« médecin ») ou à d'autres membres de l'équipe de recherche. Si vous ne comprenez pas une section du présent formulaire de consentement, veuillez consulter un membre de l'équipe de recherche.

CONTEXTE

L'espérance de vie des Américains et des Canadiens augmente progressivement. Or, bien que les gens vivent plus longtemps, la démence n'apparaît pas plus tard. Il a été démontré que certaines mesures thérapeutiques autres que des médicaments contribuent au maintien de la santé du cerveau des aînés. C'est notamment le cas de certaines formes d'entraînement cérébral progressif informatisé (programmes informatiques) qui visent des zones du cerveau et des processus précis touchant la concentration et la vigilance.

OBJECTIF DE LA PRÉSENTE ÉTUDE DE RECHERCHE

L'objectif de la présente étude est d'évaluer et de valider les bénéfices de deux programmes d'entraînement cognitif informatisés sur la santé neurologique et neuropsychologique des aînés.

Afin d'évaluer si l'entraînement cognitif a eu un effet sur les systèmes de votre cerveau touchant la concentration et la vigilance, nous comparerons des images de votre cerveau saisies par TEP et IRM avant et après la période d'entraînement (qui vous sera expliquée en détail ci-dessous).

Dans le cadre de la présente étude de recherche, nous recruterons environ 108 participants en bonne santé en vue que 90 d'entre eux puissent terminer l'étude avec succès. Nous recruterons des hommes et des femmes de 65 ans et plus.

DESCRIPTION DES PROCÉDURES DE RECHERCHE

Cette étude de recherche se tiendra à l'Institut-hôpital neurologique de Montréal.

I. Quantité de visites et leur durée

Votre participation à la présente étude de recherche s'étendra sur 6 mois et inclura 4 ou 5 visites à la clinique. Au cours de cette période, vous devrez effectuer les séances d'exercices d'entraînement qui vous sont attribuées, soit 7 séances quotidiennes par semaine pendant 10 semaines. Chaque séance durera environ 30 minutes.

II. Aperçu de la participation à l'étude

La présente étude se divise en 6 périodes :

1. Visite de sélection (V0)
- Vous serez sujet à certaines évaluations préalables pour déterminer si vous pouvez participer à l'étude.
- Vous effectuerez 1 visite à la clinique.
- Cette visite durera environ 1 heure.

2. Visite de référence (V1)
- Vous serez sujet à des procédures additionnelles (l'examen cognitif, la TEP et l'IRM).
- Cette période durera jusqu'à 4 semaines.
- Vous effectuerez 1 visite à la clinique. Au besoin, celle-ci pourra être divisée en 2 séances.
- Cette visite durera environ 3 heures.

3. Période d'orientation sur le programme et d'intervention

Avant de lancer votre entraînement, nous vous inviterons à une séance d'orientation lors de laquelle le personnel de l'étude vous présentera le programme d'entraînement en personne.

Nous vous fournirons également des instructions écrites détaillées qui présentent comment fonctionne le programme d'entraînement des semaines suivantes.

- Vous serez affecté au hasard (comme à pile ou face) à l'un ou l'autre des programmes d'entraînement cognitif informatisés, et la période d'évaluation de votre entraînement quotidien se mettra en branle.
- Cette période s'étendra sur 10 semaines.
- Vous effectuerez 1 visite à la clinique pour l'orientation sur le programme. Cette visite peut également prendre la forme d'un appel téléphonique ou vidéo à distance qui sera organisé par le personnel de l'étude. Par la suite, le personnel de l'étude effectuera un suivi hebdomadaire auprès de vous afin de répondre à vos questions et de régler tout problème. Ce suivi aura lieu par courriel.
- Cette visite durera 1 heure.

4. Visite après intervention (V2)
- Une fois vos 10 semaines d'entraînement achevées, vous effectuerez une visite à la clinique pour des évaluations après intervention.
- Après cette visite, vous n'aurez plus accès aux applications utilisées dans le cadre de l'intervention.
- Cette visite durera environ 3 heures. Au besoin, celle-ci pourra être divisée en 2 séances.

5. Période sans contact
- La visite après intervention sera succédée par une période de suivi de 3 mois sans utilisation additionnelle du programme. L'équipe de l'étude ne communiquera pas avec vous sauf pour confirmer la visite de suivi.

6. Visite de suivi (fin de l'étude, V3)
- Après la période de trois mois sans contact, nous vous inviterons à la clinique pour une dernière visite afin d'effectuer l'évaluation cognitive finale.
- Cette visite aura lieu 6 mois après votre inscription initiale.
- Cette visite durera environ 1,5 heure.

PROGRAMMES D'ENTRAÎNEMENT INFORMATISÉS

La présente étude inclut deux interventions informatisées : l'entraînement de la vitesse et de l'attention, et l'entraînement de la fonction exécutive. Vous serez affecté à l'un des groupes suivants :

Groupe 1 : effectuera l'entraînement informatisé sur ordinateur axé sur la vitesse et l'attention.
Groupe 2 : effectuera l'entraînement informatisé sur ordinateur axé sur la fonction exécutive.

Il s'agit d'une étude randomisée, ce qui signifie que vous serez affecté à l'un des deux groupes. Vous ne pouvez pas choisir votre groupe : la répartition est aléatoire, comme un tir à pile ou face. Une personne

sur deux (50 %) effectuera l'entraînement informatisé sur ordinateur axé sur la vitesse et l'attention, et une personne sur deux (50 %) l'entraînement informatisé sur ordinateur axé sur la fonction exécutive.

Vous devrez effectuer les séances d'exercices d'entraînement qui vous sont attribuées, soit 7 séances quotidiennes par semaine pendant 10 semaines. Chaque séance durera environ 30 minutes.

Les deux groupes pourront effectuer les exercices à partir de tout ordinateur doté d'une connexion à Internet, donc dans le confort de votre demeure. Au besoin, nous pouvons vous fournir un ordinateur portable ou une tablette électronique. Vous aurez l'occasion de nous poser vos questions sur le programme informatique afin de garantir que vous en comprenez bien le fonctionnement.

Nous vous rappelons de ne ***pas*** discuter des détails du programme d'entraînement avec vos collègues, vos amis, vos connaissances ou le personnel de l'étude.

Veuillez prendre en considération les éléments suivants de l'utilisation du programme :

Lieu de l'utilisation : vous pouvez utiliser le programme en clinique ou à votre domicile. Nous vous prêterons un appareil mobile (une tablette) avec lequel vous pourrez utiliser le programme. Vous pouvez également choisir d'effectuer les entraînements sur l'un de vos appareils.

Fatigue : Nous nous attendons à ce que certains participants ne soient pas en mesure d'achever l'intégralité d'une séance de 30 minutes d'un seul coup. Par conséquent, les participants pourront également choisir de diviser la séance en périodes plus courtes, par exemple 15 minutes le matin et 15 minutes plus tard en journée. Les participants pourront mettre la séance en pause à tout moment pour prendre un peu de repos avant de reprendre le tout là où ils l'avaient laissé.

Vous pouvez discuter de ces options avec l'équipe de l'étude, qui établira l'horaire vous convenant le mieux. Si vous ressentez le besoin de réduire le nombre de séances par semaine, l'équipe de l'étude collaborera avec vous pour établir un horaire d'entraînement qui vous conviendra.

III. Procédures de l'étude

Au cours de votre participation à la présente étude de recherche, vous suivrez les procédures ci-dessous :

**DESCRIPTION DES PROCÉDURES DE L'ÉTUDE**	
**Procédure**	**Description**
Antécédents médicaux et caractéristiques sociodémographiques	Nous recueillerons l'intégralité de vos antécédents médicaux, y compris tout médicament que vous prenez actuellement ou avez déjà consommé, et toute autre thérapie ou intervention que vous avez suivie ou subie. Au cours de l'étude, nous vous demanderons de nous indiquer tout changement perçu de votre santé ainsi que toute autre modification de votre état, par exemple la prise de nouveaux médicaments.<br><br>Nous vous demanderons également votre âge, votre race et votre ethnicité.

Questionnaires et évaluations	Nous évaluerons votre état cognitif à l'aide de l'Évaluation cognitive de Montréal (MoCA).<br><br>Nous vous demanderons de répondre à quelques questions sur l'état de votre santé mentale par l'entremise de l'Échelle de dépression gériatrique — Version courte et de l'Échelle d'évaluation de Columbia sur la gravité du risque suicidaire (C-SSRS).
Évaluations neuropsychologiques	Un membre de l'équipe de l'étude effectuera une évaluation neuropsychologique pour mesurer certains déclins cognitifs liés à l'âge précis.
TEP et radiotraceur FEOBV	La TEP est une procédure de médecine nucléaire où une très faible quantité d'un produit chimique (dose de radiotraceur FEOBV) est injectée dans votre corps pour nous aider à balayer des zones précises de votre cerveau. Lorsqu'il est injecté par intraveineuse, ce produit chimique circule dans le sang jusqu'à ce qu'il atteigne son objectif, où il reste brièvement en place avant de se désintégrer. **Le radiotraceur FEOBV utilisé dans la présente étude est expérimental et n'a pas été approuvé par Santé Canada pour une utilisation dans le cadre de pratiques médicales ordinaires.** Cependant, Santé Canada ne s'oppose pas à son utilisation dans la présente étude.<br><br>Ce radiotraceur chimique contient un élément atomique le rendant détectable par la caméra de TEP, qui produit ainsi une image nette de zones précises de votre cerveau.<br><br>Au cours de ce processus, le radiotraceur émettra une très petite quantité de radioactivité qui peut être détectée par une caméra de TEP sophistiquée. À l'aide d'ordinateurs puissants, les chercheurs peuvent alors étudier la distribution du produit chimique au sein du cerveau. **Comme une très petite quantité du produit chimique (dose de radiotraceur) est administrée, ses effets sont indétectables sur un individu donné.**

	Toutes les séances de TEP dans le cadre de votre participation seront supervisées par un technicien qualifié en médecine nucléaire. Un médecin sera disponible au besoin. Après votre arrivée à l'unité de TEP du Neuro, vous devrez remplir quelques questionnaires de routine sur votre condition physique globale. Ensuite, un cathéter-aiguille mince sera inséré dans une veine de votre bras. Le FEOBV sera administré par l'entremise de ce cathéter.<br><br>Vous devrez alors attendre environ 180 minutes, le temps que le produit chimique se distribue convenablement dans votre cerveau. Pendant ce temps, vous devez rester au repos, mais pourrez utiliser la salle de bains et marcher au besoin.<br><br>Par la suite, nous vous demanderons de vous étendre sur un lit qui s'insérera dans une ouverture cylindrique pour le processus de balayage, qui durera environ 40 minutes.<br><br>Cet appareil est entièrement passif et ne génère aucun champ électrique (ou autre) pouvant être nocif.
IRM	Dans le cadre de ce test, un balayage détaillé (une image) de votre cerveau et de votre colonne vertébrale est produit à l'aide d'un aimant et d'ondes radio. Un appareil d'IRM contient un aimant en forme de beigne incluant souvent un tunnel en son centre. Vous devrez vous étendre sur une table qui se glissera dans le tunnel. Des oreillers appuieront votre tête. Si les espaces restreints vous effraient ou vous causent de l'inconfort, veuillez le mentionner au personnel de l'étude.<br><br>Si un appareil a été implanté dans votre corps, veuillez le mentionner au personnel de l'étude.<br><br>À l'intérieur de l'appareil de balayage, vous entendrez un ventilateur et sentirez un courant d'air. Vous pourriez également entendre des tapotements ou des craquements au fil du balayage. Des bouchons d'oreille ou des écouteurs vous seront fournis pour réduire le bruit. Vous devez rester le plus immobile possible au cours du balayage. Il se peut que nous vous demandions de retenir votre souffle pendant quelques instants.<br><br>Vous aurez accès à un bouton vous permettant de communiquer à tout moment avec l'équipe chargée de l'IRM, et vous pourrez leur parler entre chaque balayage par l'entremise d'un interphone.

	Le personnel de l'étude vous indiquera précisément comment vous préparer en vue des balayages. Vous devrez enlever tous vos articles métalliques et porter une blouse d'hôpital.<br><br>Le balayage peut prendre jusqu'à 20 minutes.
Évaluation du comportement	Afin de mesurer votre fonction acétylcholinique (comment vos muscles répondent), nous mesurerons la variabilité de votre fréquence cardiaque et/ou l'élargissement de vos pupilles (pupillométrie) lors des évaluations dans le cadre de la formation préalable aux exercices, ou au besoin.<br><br>Nous effectuerons la pupillométrie à l'aide de l'appareil Tobii Pro Glasses 2. Il s'agit de lunettes qui surveillent vos mouvements oculaires alors que vous les portez.<br><br>La variabilité de votre fréquence cardiaque sera mesurée à l'aide d'un bracelet moniteur.
Communication par courriel avec le personnel de l'étude	Dans le cadre de la présente étude, les participants aux essais cliniques et le personnel de l'étude communiqueront par courriel.<br><br>Le personnel de l'étude communiquera avec les participants par courriel au sujet de rendez-vous à venir, de mises à jour hebdomadaires sur l'utilisation des programmes ou d'autres renseignements importants sur l'étude, dont les instructions expliquant comment compléter les activités requises à distance. Les participants pourront également poser des questions au personnel de l'étude par courriel.
Utilisation sécuritaire de l'application pour appareil mobile	Comme nous sommes conscients que l'utilisation d'une application pour appareil mobile peut représenter un risque en matière de confidentialité, nous avons mis en place d'importantes garanties afin d'éviter les écueils potentiels.<br><br>La sécurité des données électroniques est garantie aux niveaux du serveur, de l'utilisateur et de la base de données.<br><br>Nous vous offrirons un identifiant dépersonnalisé avec lequel accéder au programme d'entraînement. Le programme ne recueille, ne transmet et n'emmagasine aucun renseignement personnel outre les dates où les exercices d'entraînement sont complétés. Le programme informatique ne recueille également aucune donnée sur l'emplacement géographique.

RESPONSABILITÉS DES PARTICIPANTS

- Effectuez toutes les visites requises dans le cadre de l'étude.
- Effectuez une séance d'entraînement par jour. Chaque séance prend environ 30 minutes.
    - Vous êtes autorisé à prendre une pause pendant la séance ou à la fractionner à travers la journée.
- Consultez vos courriels et répondez aux messages de suivi du coordonnateur de l'étude.
- Suivez les instructions du chercheur ou du personnel de l'étude.
- Veuillez éviter de discuter des détails du programme d'entraînement informatisé avec quiconque.

AVANTAGES DÉCOULANT DE LA PRÉSENTE ÉTUDE DE RECHERCHE

Votre participation à la présente étude ne vous procurera aucun avantage direct. Nous espérons toutefois que les résultats de l'étude contribueront au progrès de la science dans le domaine étudié.

RISQUES ASSOCIÉS À LA PRÉSENTE ÉTUDE DE RECHERCHE

Un possible risque associé à la présente étude est une violation de votre confidentialité ou l'utilisation de vos renseignements personnels par une tierce partie. Les mesures décrites dans la section Confidentialité ci-dessous expliquent comment nous comptons limiter ce risque.

Inconfort lors des évaluations et de l'entraînement.
Certaines personnes pourraient ressentir de la fatigue ou de la frustration dans le cadre des évaluations et de l'entraînement informatisé. Pour réduire l'inconfort éventuel, nous vous encourageons à prendre des pauses, qui seront mises à l'horaire dans vos séances. Si vous ressentez une pression excessive, vous pouvez mettre fin aux tests à tout moment.

Absence de retour sur les évaluations.
Aucun participant ne recevra un retour sur les résultats de ses évaluations, ce qui pourrait être source de frustration.

Risques de la communication par courriel.
La communication par courriel présente certains risques qui sont amplifiés si aucun service de chiffrement n'est utilisé. Les risques de l'envoi et de la réception de courriels incluent, sans s'y limiter :
- Des tierces parties pourraient intercepter les messages.
- Si vous envoyez un message à partir d'un appareil appartenant à votre employeur ou recevez un message sur cet appareil, ledit employeur pourrait avoir le droit de lire et d'enregistrer ce message. Votre fournisseur de services Internet ou de téléphonie cellulaire pourrait aussi avoir le droit de lire et d'enregistrer vos courriels.
- Une copie enregistrée de vos courriels pourrait persister sur un appareil ou un ordinateur même si elle est effacée.
- Si vous saisissez accidentellement la mauvaise adresse courriel, le message pourrait être envoyé au mauvais destinataire.
- Des virus informatiques peuvent se répandre par courriel.
- Des tierces parties pourraient accéder aux messages stockés dans un appareil perdu, volé ou jeté.

- Si un participant à l'étude change d'adresse courriel sans en aviser le personnel de l'étude, ce participant pourrait manquer certains messages.

Violation de la vie privée
Un risque possible pour les participants est la divulgation de renseignements cliniques à la suite d'une violation de la confidentialité. La participation à toute étude de recherche, y compris la présente, peut entraîner une violation de votre vie privée. Les procédures suivantes ont été mises en place pour garantir le respect de votre vie privée : (1) tout le personnel de l'étude a suivi des séances de formation protocolaires formelles soulignant l'importance de la confidentialité, (2) l'étude respecte des procédures spécifiquement conçues pour protéger la vie privée des participants, et (3) des mécanismes formels limitent l'accès aux renseignements pouvant lier les données à des participants précis.

TEP
Lors de la séance d'imagerie par TEP, les participants pourraient ressentir un pincement lors de l'insertion du cathéter dans une veine. Comme pour toute autre injection, il existe un très faible risque d'infection du site de l'injection. Afin de réduire ce risque, l'équipement utilisé est stérilisé et la zone de la peau touchée est désinfectée soigneusement. Par ailleurs, la période d'immobilité prolongée sur le canapé pourrait être source d'agitation ou d'inconfort pour certains participants. L'imagerie par TEP requiert l'injection d'un agent précis (FEOBV) qui est normalement absent du corps humain. Comme tout autre produit chimique ou pharmaceutique, cet agent peut entraîner des réactions allergiques ou indésirées. Toutefois, de telles réactions n'ont jamais été observées pour les doses utilisées dans le cadre de la présente étude.

Comme le FEOBV est un produit radioactif, les participants seront par conséquent exposés à une faible dose d'irradiation (mesurée en millisieverts, ou mSv) qui dépasse la dose à laquelle un individu est habituellement exposé dans la vie quotidienne (irradiation naturelle provenant de l'environnement, de rayons cosmiques, etc.) ou dans le cadre d'interventions médicales (radiographies diagnostiques, radiothérapie, etc.). Le corps des participants n'émettra plus aucune trace de radioactivité après quelques heures (après 20 heures, toute radioactivité sera essentiellement indétectable). Dans le cadre de la discussion des risques découlant de l'exposition à la faible quantité d'irradiation nécessaire pour la TEP, il peut être question d'un éventuel cancer qui n'aurait autrement pas apparu. De manière générale, il est connu qu'à partir de certaines doses, l'irradiation augmente les risques de cancer. Or, comme seules de très faibles doses sont requises pour l'imagerie par TEP, aucun lien n'a été observé entre celle-ci et l'apparition de cancers. Par conséquent, le risque est faible. Le radiotraceur FEOBV utilisé dans la présente étude est expérimental et n'a pas été approuvé par Santé Canada pour une utilisation dans le cadre de pratiques médicales ordinaires. Cependant, Santé Canada ne s'oppose pas à son utilisation dans la présente étude. **La limite nationale d'exposition à l'irradiation dans le cadre d'études de recherche s'établit à 50 mSv par année. Question de ne pas dépasser cette limite, vous devrez informer l'équipe de l'étude de tout balayage que vous avez subi dans les 12 derniers mois.**

Vous devez également déclarer votre participation à la présente étude à tout chercheur vous demandant de participer à une étude mettant en cause la radioactivité. Les deux TEP dans le cadre de l'étude vous exposeront à une dose d'environ 11 à 15,4 mSv.

Risques propres au FEOBV :
Aucune réaction indésirable au FEOBV n'a été signalée à ce jour. Toutefois, certains risques potentiels sont listés ci-dessous :

Puisque les risques du FEOBV sur les femmes enceintes, les enfants à naître ou les enfants de femmes qui allaitent sont inconnus, l'utilisation du FEOBV et à éviter auprès des femmes enceintes ou qui allaitent. Par conséquent, les femmes enceintes ou qui allaitent ne peuvent pas participer à la présente étude.

Comme le FEOBV est un produit radioactif, la petite quantité de ce produit nécessaire pour l'étude peut laisser des traces dans l'urine et les matières fécales des participants, qui pourraient contaminer les surfaces avec lesquelles elles entrent en contact. Pendant 12 heures après l'injection du FEOBV, les participants devront utiliser une toilette plutôt qu'un urinoir et devront actionner la chasse d'eau à plusieurs reprises.

IRM

Pendant ce test, vous serez exposé à un puissant champ magnétique et à des ondes radio. Aucun effet secondaire nocif à long terme n'a été observé pour un examen de ce genre.

Un IRM peut être plutôt bruyant, et certaines personnes peuvent ressentir de l'inconfort alors qu'elles reposent dans l'appareil. Nous vous fournirons des bouchons d'oreille pour réduire le bruit.

Vous pourriez brièvement ressentir une certaine claustrophobie (un sentiment d'enfermement) lors de l'IRM. Le mélange de bruit et de sentiment d'enfermement peut entraîner un inconfort chez certains. Vous serez en communication constante avec le technicien chargé de l'IRM, et vous pourrez appuyer sur un « bouton d'appel » si vous ne vous sentez pas bien et souhaitez mettre un terme à l'intervention.

Si l'une des contre-indications suivantes s'applique à vous, veuillez en informer le personnel de l'étude :

- Stimulateur cardiaque
- Clip pour anévrysme
- Clip cardiaque/vasculaire
- Valve prothétique
- Prothèse métallique
- Vous êtes enceinte ou comptez le devenir
- Fragments métalliques dans le corps
- Timbres transdermiques (doivent être retirés avant l'examen; veuillez apporter un timbre additionnel que vous pourrez appliquer après le balayage)

DÉSAGRÉMENTS LIÉS AUX PROCÉDURES DE L'ÉTUDE

Voici les seuls désagréments envisageables pouvant découler d'une participation à l'étude :

- Le temps nécessaire pour compléter les entraînements quotidiens.

PARTICIPATION VOLONTAIRE ET DROIT DE RETRAIT

Votre participation à ce projet de recherche est volontaire. Vous êtes donc libre de refuser d'y participer. Vous pouvez également vous retirer du projet *en cours* à n'importe quel moment, sans avoir à donner de raisons, en informant l'équipe de recherche. Votre décision de ne pas participer à ce projet de recherche ou de vous en retirer n'aura aucune conséquence sur la qualité des soins et des services auxquels vous

avez droit ou sur votre relation avec les équipes qui les dispensent. Si des renseignements nouvellement acquis pourraient influencer votre consentement à participer au reste de l'étude, vous en serez informé dans un délai raisonnable.

Le médecin responsable de ce projet de recherche ou le comité d'éthique de la recherche peuvent mettre fin à votre participation, sans votre consentement. Cela peut se produire si de nouvelles découvertes ou informations indiquent que votre participation au projet n'est plus dans votre intérêt, si vous ne respectez pas les consignes du projet de recherche ou encore s'il existe des raisons administratives d'abandonner le projet.

Si vous vous retirez du projet ou êtes retiré du projet, vous pouvez également demander que les données à votre sujet ayant déjà été recueillies soient retirées de l'étude.

Vous pouvez choisir de cesser d'utiliser le programme informatisé, mais de continuer de participer à l'étude en effectuant une *visite après intervention (V2)* et une *visite de suivi (V3)*.

Toute nouvelle connaissance acquise durant le déroulement du projet qui pourrait affecter votre décision de continuer à participer à ce projet vous sera communiquée rapidement.

CONFIDENTIALITÉ

Durant votre participation à ce projet de recherche, le médecin responsable de ce projet ainsi que les membres de son personnel de recherche recueilleront, dans un dossier de recherche, les renseignements vous concernant et nécessaires pour répondre aux objectifs scientifiques de ce projet de recherche.

Ces renseignements peuvent comprendre les informations contenues dans votre dossier médical, dont votre identité, concernant votre état de santé passé et présent, vos habitudes de vie ainsi que les résultats de tous les tests, examens et procédures qui seront réalisés. Le dossier de recherche à votre nom pourrait également contenir d'autres renseignements, dont votre nom, votre sexe, votre âge, votre date de naissance, votre origine ethnique et d'autres dates tirées des données recueillies (comme les dates d'achèvement des exercices d'entraînement qui vous sont assignés).

Tous les renseignements recueillis demeureront confidentiels dans les limites prévues par la loi. Vous ne serez identifié que par un numéro de code. La clé du code reliant votre nom à votre dossier de recherche sera conservée par le médecin responsable de ce projet de recherche.

Pour assurer votre sécurité, un formulaire confirmant votre participation à une étude clinique sera ajouté à votre dossier médical. Par conséquent, toute personne ou compagnie à qui vous donnerez accès à votre dossier médical aura accès à ces informations.

Le médecin responsable de ce projet de recherche fera parvenir, au commanditaire ou à ses représentants, les données codées vous concernant. Toutefois, une fois les données codées transférées hors du Canada, des protections équivalentes à celles en vigueur au Canada et au Québec seront mises en œuvre.

Les données de l'étude seront emmagasinées pendant 25 ans par le médecin responsable de l'étude.

Les données de recherche pourront être publiées ou faire l'objet de discussions scientifiques, mais il ne sera pas possible de vous identifier.

Pour des raisons de contrôle, il est possible qu'une personne mandatée par le commanditaire de l'étude, l'établissement, le Comité d'éthique de la recherche ou le NIH examine des fichiers de l'étude de recherche incluant des documents pouvant vous identifier. Toutes ces personnes et organisations respectent des politiques de confidentialité.

DÉPÔT DE DONNÉES

Deux ans après la fin des essais, nous emmagasinerons toutes les données chiffrées sur tous les participants de l'étude, y compris les données de TEP et d'IRM, sur une plateforme examinée et approuvée par l'établissement. En vue de protéger votre vie privée, les images seront dépersonnalisées, c'est-à-dire que votre visage sera masqué.

En emmagasinant toutes les données tirées de la présente étude dans une base de données, nous permettrons à des chercheurs universitaires non affiliés aux présents essais d'effectuer davantage d'analyses sur les résultats obtenus. Ce dépôt de données aura lieu deux ans après la fin de l'étude de manière à ce que les objectifs de l'étude principale puissent tous être analysés avant que des chercheurs non affiliés aient accès aux données.

Le partage des données sur une base de données examinée par l'établissement est ouvert. Cela signifie que tout chercheur peut accéder aux données de l'étude ou bien librement, ou bien après s'être inscrit auprès de la plateforme. Toutefois, comme l'indique la section Confidentialité, des mesures de précaution s'appliquent à tout renseignement pouvant être publié ou partagé afin que votre identité demeure confidentielle.

DÉCOUVERTES FORTUITES

Les découvertes fortuites significatives incluent toute découverte inattendue faite au cours de l'étude et pouvant avoir un impact important sur votre bien-être actuel ou futur, ou sur celui des membres de votre famille. En cas de découverte fortuite significative vous touchant dans le cadre de la présente étude, cette découverte sera communiquée avec vous et un professionnel de la santé de votre choix.

FINANCEMENT DU PROJET DE RECHERCHE

Le chercheur et l'établissement ont reçu du financement du National Institute on Aging afin de mener à bien le présent projet de recherche.

POSSIBILITÉS DE COMMERCIALISATION

Les programmes d'entraînement informatisés sous étude sont développés par Posit Science Corporation. Les chercheurs principaux de Posit Science Corporation pourraient profiter d'éventuelles activités commerciales découlant des résultats de la présente étude. Toutefois, vous ne recevrez aucun avantage financier.

CONFLIT D'INTÉRÊTS

Les chercheurs principaux sont employés et actionnaires de Posit Science Corporation. Les chercheurs principaux de Posit Science Corporation pourraient profiter d'éventuelles activités commerciales découlant des résultats de la présente étude.

RÉMUNÉRATION

Vous serez rémunéré pour compenser les coûts encourus et les inconvénients subis au cours la présente étude de recherche. Si vous vous retirez de l'étude ou en êtes retiré avant que cette dernière soit achevée, votre rémunération sera proportionnelle au nombre de visites effectuées.

La rémunération se répartit ainsi :

- Visite de référence, y compris l'imagerie par TEP (V1) : 40 $
- Vous recevrez 13 $ pour chaque tranche de 10 séances d'entraînement achevées (au plus 91 $ si vous complétez les 70 séances) au cours de la période d'intervention.
- Visite après intervention, y compris l'imagerie par TEP (V2) : 40 $
- Visite de suivi (V3) : 40 $

Les participants qui achèvent l'intégralité de l'étude recevront 211 $.

Les participants auxquels une tablette électronique a été prêtée recevront la rémunération qui leur est due pour toutes les activités relatives à l'étude au cours de la *période d'intervention* une fois la tablette rendue au personnel de l'étude lors de la *visite après intervention* (V2). Les participants s'étant retirés de l'étude seront rémunérés pour chaque visite complétée une fois que la tablette leur ayant été prêtée aura été rendue au personnel de l'étude.

EN CAS DE PREJUDICE

Si vous deviez subir quelque préjudice que ce soit par suite de toute procédure reliée à ce projet de recherche, vous recevrez tous les soins et services requis par votre état de santé.

En acceptant de participer à ce projet de recherche, vous ne renoncez à aucun de vos droits et vous ne libérez pas le médecin responsable de ce projet de recherche, le commanditaire et l'établissement de leur responsabilité civile et professionnelle.

IDENTIFICATION DES PERSONNES-RESSOURCES

Si vous avez des questions ou éprouvez des problèmes en lien avec le projet de recherche, ou si vous souhaitez vous en retirer, vous pouvez communiquer avec le médecin responsable ou avec une personne de l'équipe de recherche au numéro suivant:514-398-2733

Pour toute question concernant vos droits en tant que participant à ce projet de recherche ou si vous avez des plaintes ou des commentaires à formuler, vous pouvez communiquer avec :la commissaire aux patients de l'Institut-hôpital neurologique de Montréal au numéro de téléphone suivant : (514) 934-1934, poste 48306.

APERÇU DES ASPECTS ÉTHIQUES DE L'ÉTUDE

Le Comité d'éthique de la recherche du Centre universitaire de santé McGill a évalué la présente étude et est responsable de sa surveillance du point de vue éthique.

Titre de l'étude de recherche : Améliorer la santé neurologique du vieillissement grâce à un exercice informatisé basé sur la neuroplasticité

SIGNATURES

Signature du participant

J'ai pris connaissance du formulaire d'information et de consentement. On m'a expliqué le projet de recherche et le présent formulaire d'information et de consentement. On a répondu à mes questions et on m'a laissé le temps voulu pour prendre une décision. Après réflexion, je consens à participer à ce projet de recherche aux conditions qui y sont énoncées.

J'autorise l'équipe de recherche à avoir accès à mon dossier médical aux fins de la présente étude.

J'autorise un membre de l'étude de recherche à communiquer avec moi à l'avenir pour me demander si d'autres études pourraient m'intéresser.

Oui ☐ Non ☐ Si oui, veuillez indiquer vos coordonnées : ___________________________

____

Nom du participant Signature Date

Signature de la personne qui obtient le consentement

J'ai expliqué au participant le projet de recherche et le présent formulaire d'information et de consentement et j'ai répondu aux questions qu'il m'a posées.

____

Nom de la personne qui obtient le consentement Signature Date
