# Supplementary material for: Effects of Computerized Cognitive Training on Vesicular Acetylcholine Transporter Levels using [18F]Fluoroethoxybenzovesamicol Positron Emission Tomography in Healthy Older Adults: Results from the Improving Neurological Health in Aging via Neuroplasticity-based Computerized Exercise (INHANCE) Randomized Clinical Trial
Source: JMIR Serious Games. 2025 Oct 13;13:e75161. doi: 10.2196/75161 (PMC12559824; doi:10.2196/75161)
Supplement: Multimedia Appendix 2 [file games_v13i1e75161_app2.pdf]

Multimedia Appendix 2: PET and MRI acquisition and data analysis and Supporting Figure 1

PET and MRI acquisition and data analysis

A structural MRI was acquired during the imaging portion of the baseline visit to co-register the PET data.[1] All participants underwent a structural T1-weighted MRI scan (3T Siemens Prisma) using the 3D magnetization-prepared rapid gradient echo (MPRAGE) sequence, followed by a [18F]FEOBV-PET scan using the Siemens High-Resolution Research Tomograph (HRRT) at the McConnell Brain Imaging Centre of the Montreal Neurological Institute-Hospital.

Concurrent T1-weighted MPRAGE images were acquired with the related acquisition parameters being echo time/repetition time = 2.98 ms/2300 ms, inversion time = 900 s, flip angle 9°, 1 mm isotropic resolution, and image dimensions 192x256x256.

Participants were positioned lying on their back for the PET imaging session and received a slow bolus intravenous injection of [18F]FEOBV with radioactivity doses ranging from 350-400 MBq, corresponding to 8.05mSv to 9.2mSV, via a fine needle-catheter inserted into an arm vein. PET data acquisition started 180 minutes after injection, for a duration of 30 minutes, divided into 6 frames of 5 minutes each.

A transmission scan of 5 minutes was conducted with a rotating point source of [137Cs] for the HRRT PET images in order to perform attenuation correction. PET images were reconstructed using an ordinary Poisson-ordered subset expectation maximization (OP-OSEM) algorithm (10 iterations, 16 subsets) with resolution recovery, correcting for scatter, randoms, attenuation, decay, and dead time. Motion correction was applied, and time-averaged PET data (6 × 5-min frames) were used to create a static image. The final reconstruction was performed on a 256 × 256 × 207 matrix (voxel size: 1.22 mm^3, spatial resolution: 2.3 mm full-width at half-maximum (FWHM)) with no post-reconstruction smoothing or zoom.

PET preprocessing was performed using SPM12 in MATLAB. MRIs were segmented (gray matter, white matter, cerebrospinal fluid), bias-corrected, and spatially normalized to the MNI 152 asymmetrical template (MNI152 ONLine 2009 cAsym template) using DARTEL, with identical transformations applied to PET images. PET images were aligned to each subject's MRI, and Müller-Gärtner partial volume correction was applied using the PETPVE toolbox. A 6 mm FWHM Gaussian smoothing kernel was used to reduce noise.

Standardized Uptake Value Ratios (SUVR) were used to quantify FEOBV binding. SUVRs were computed for spatially normalized PET images using a white matter mask as the reference region.[2,3] The Hammers atlas (MNI space)[4] was used to extract the SUVR in the target region (anterior cingulate cortex). A binary white matter mask was created and then eroded to reduce contamination from neighboring regions. The final eroded white matter mask was applied to the PET images to calculate mean tracer uptake for SUVR calculations.

Supporting Figure 1

SUVR images at pre-test (first row) and post-test (second row) for the intervention (top) and active control (bottom).

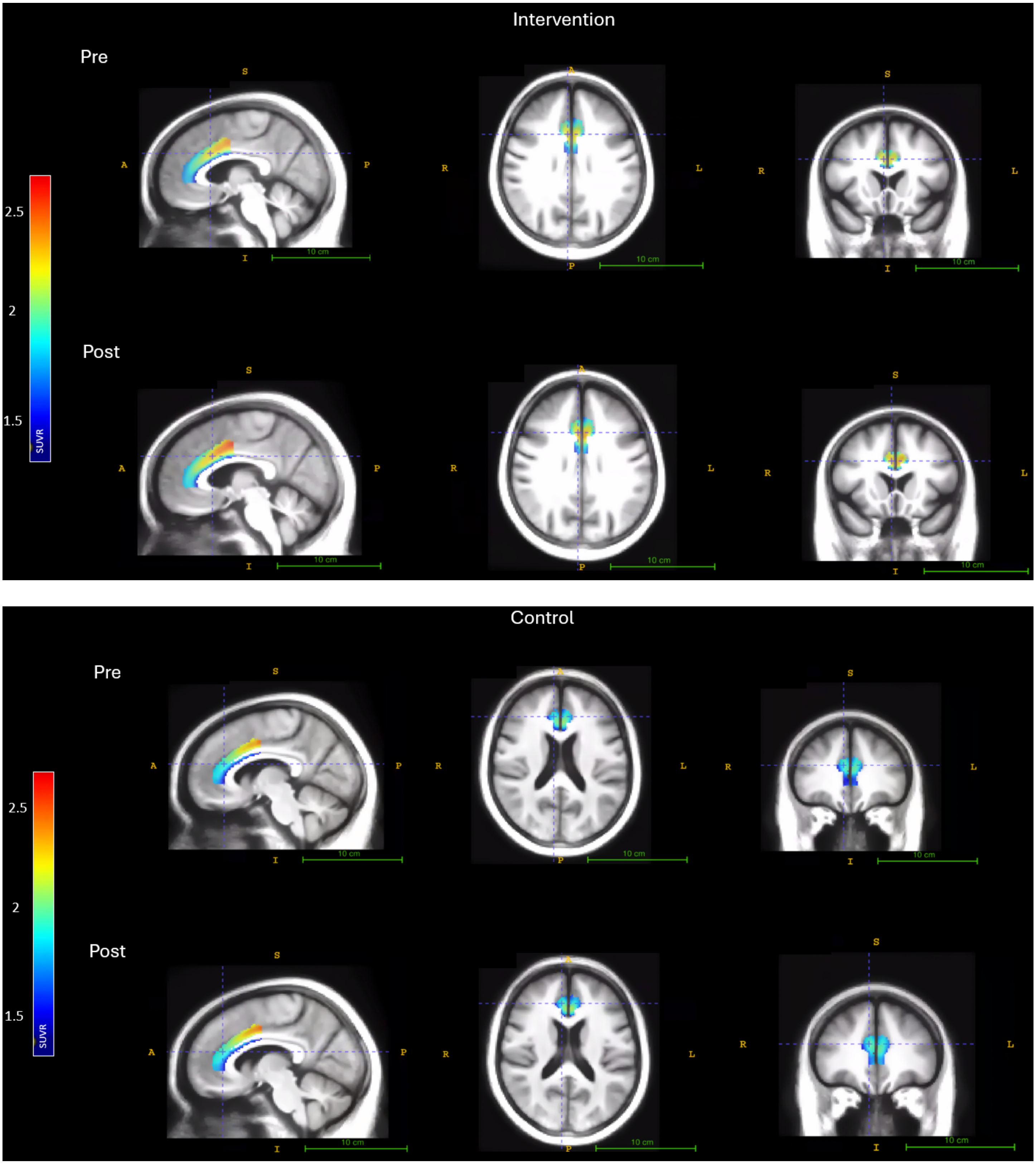

References

1. Attarha M, Pelegrino AC de F, Toussaint PJ, Grant SJ, Vleet TV, Villers-Sidani E de. Improving Neurological Health in Aging Via Neuroplasticity-Based Computerized Exercise: Protocol for a Randomized Controlled Trial. *JMIR Research Protocols*. 2024;13(1):e59705. doi:10.2196/59705

2. Nejad-Davarani S, Koeppe RA, Albin RL, Frey KA, Müller MLTM, Bohnen NI. Quantification of brain cholinergic denervation in dementia with Lewy bodies using PET imaging with [18F]-FEOBV. *Mol Psychiatry*. 2019;24(3):322-327. doi:10.1038/s41380-018-0130-5

3. Aghourian M, Legault-Denis C, Soucy JP, et al. Quantification of brain cholinergic denervation in Alzheimer's disease using PET imaging with [18F]-FEOBV. *Mol Psychiatry*. 2017;22(11):1531-1538. doi:10.1038/mp.2017.183

4. Hammers A, Allom R, Koepp MJ, et al. Three-dimensional maximum probability atlas of the human brain, with particular reference to the temporal lobe. *Hum Brain Mapp*. 2003;19(4):224-247. doi:10.1002/hbm.10123
