# Supplementary material for: Effects of Computerized Cognitive Training on Vesicular Acetylcholine Transporter Levels using [18F]Fluoroethoxybenzovesamicol Positron Emission Tomography in Healthy Older Adults: Results from the Improving Neurological Health in Aging via Neuroplasticity-based Computerized Exercise (INHANCE) Randomized Clinical Trial
Source: JMIR Serious Games. 2025 Oct 13;13:e75161. doi: 10.2196/75161 (PMC12559824; doi:10.2196/75161)
Supplement: Multimedia Appendix 3 [file games_v13i1e75161_app3.pdf]

Multimedia Appendix 3: Participant-facing questionnaires in English and French

MONTREAL COGNITIVE ASSESSMENT (MoCA®)

Version 8.3 English

Name:
Education:
Sex:
Date of birth :
DATE :

VISUOSPATIAL / EXECUTIVE			POINTS
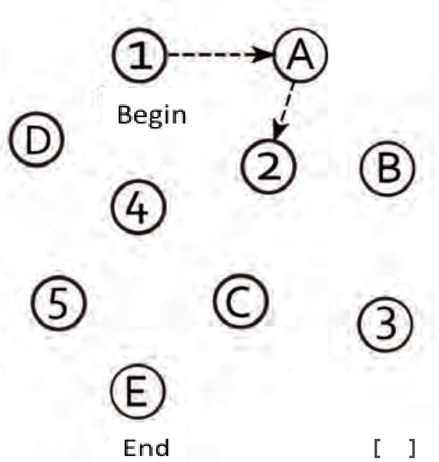 [ ]	Copy bed 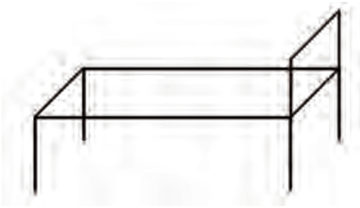 [ ]	Draw CLOCK (Five past ten) (3 points) [ ] Contour [ ] Numbers [ ] Hands	__/5

NAMING

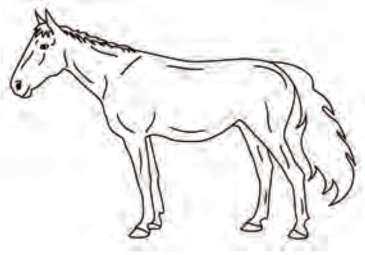 [ ]

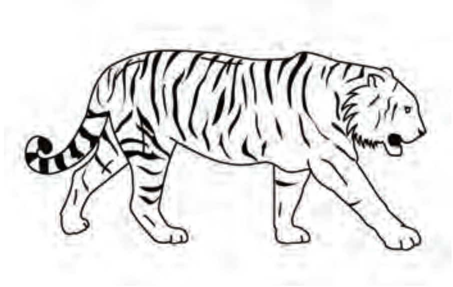 [ ]

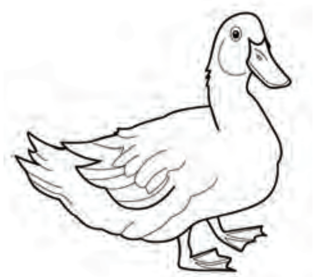 [ ]

__/3

MEMORY

Read list of words, subject must repeat them. Do 2 trials, even if 1st trial is successful. Do a recall after 5 minutes.

	LEG	COTTON	SCHOOL	TOMATO	WHITE	NO POINTS
1st TRIAL						
2nd TRIAL						

ATTENTION

Read list of digits (1 digit / sec.). Subject has to repeat them in the forward order. [ ] 2 4 8 1 5
Subject has to repeat them in the backward order. [ ] 4 2 7 __/2

Read list of letters. The subject must tap with his hand at each letter A. No points if ≥ 2 errors.
[ ] F B A C M N A A J K L B A F A K D E A A A J A M O F A A B __/1

Serial 7 subtraction starting at 60. [ ] 53 [ ] 46 [ ] 39 [ ] 32 [ ] 25
4 or 5 correct subtractions: **3 pts**, 2 or 3 correct: **2 pts**, 1 correct: **1 pt**, 0 correct: **0 pt** __/3

LANGUAGE

Repeat: The child walked his dog in the park after midnight. [ ]
The artist finished his painting at the right moment for the exhibition. [ ] __/2

Language Fluency. Name maximum number of words in one minute that begin with the letter B. [ ] ______ (N ≥ 11 words) __/1

ABSTRACTION

Similarity between e.g. banana - orange = fruit [ ] hammer - screwdriver [ ] matches - lamp __/2

DELAYED RECALL	(MIS)	Has to recall words WITH NO CUE	LEG [ ]	COTTON [ ]	SCHOOL [ ]	TOMATO [ ]	WHITE [ ]	Points for UNCUED recall only	__/5
Memory Index Score (MIS)	X3								
	X2	Category cue						MIS = ____/15	
	X1	Multiple choice cue							

ORIENTATION [ ] Date [ ] Month [ ] Year [ ] Day [ ] Place [ ] City __/6

© Z. Nasreddine MD www.mocatest.org

MIS: /15
(Normal ≥ 26/30)
Add 1 point if ≤ 12 yr education

TOTAL __/30

Administered by: ______________________________

Training and Certification are required to ensure accuracy.

MONTREAL COGNITIVE ASSESSMENT (MoCA®)
Version 8.3 Français

NOM:
Scolarité: Date de naissance:
Sexe: DATE:

VISUOSPATIAL/EXÉCUTIF

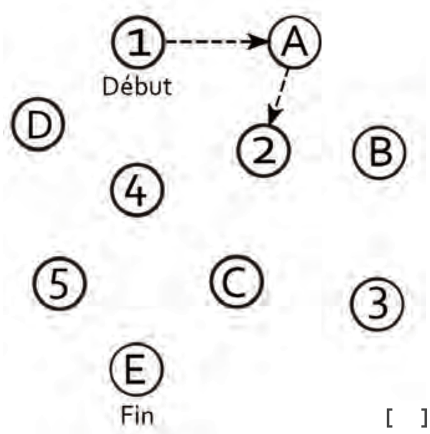

[ ]

Copier le lit

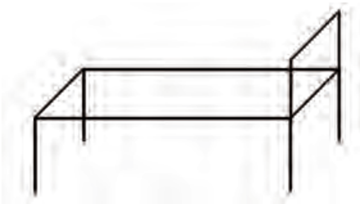

[ ]

Dessiner une HORLOGE (Dix heures et cinq minutes) (3 points)

[ ] Contour [ ] Chiffres [ ] Aiguilles

POINTS __/5

DÉNOMINATION

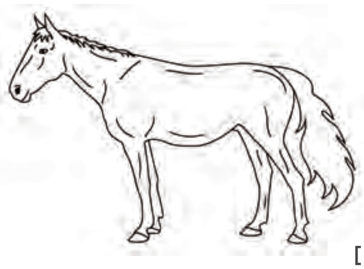

[ ]

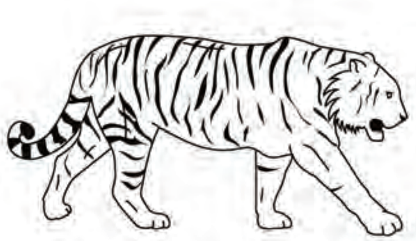

[ ]

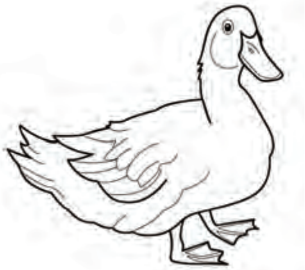

[ ]

__/3

MÉMOIRE

Lire la liste de mots, le sujet doit la répéter. Faire 2 essais même si le 1er essai est réussi. Faire un rappel après 5 minutes.

	JAMBE	COTON	ÉCOLE	TOMATE	BLANC
1er essai					
2e essai					

PAS DE POINT

ATTENTION

Lire la série de chiffres (1 chiffre/sec.). Le sujet doit la répéter dans le même ordre. [ ] 2 4 8 1 5
Le sujet doit la répéter à l'envers. [ ] 4 2 7
__/2

Lire la série de lettres. Le patient doit taper de la main à chaque lettre A. Pas de points si ≥ 2 erreurs.
[ ] F B A C M N A A J K L B A F A K D E A A A J A M O F A A B
__/1

Soustraire série de 7 à partir de 60. [ ] 53 [ ] 46 [ ] 39 [ ] 32 [ ] 25
4 ou 5 soustractions correctes: **3 pts**, 2 ou 3 correctes: **2 pts**, 1 correcte: **1 pt**, 0 correcte: **0 pt**
__/3

LANGAGE

Répéter: L'enfant a promené son chien dans le parc après minuit. [ ]
L'artiste a terminé sa toile au bon moment pour l'exposition. [ ]
__/2

Fluidité du langage. Nommer un maximum de mots commençant par la lettre «T» en 1 min. [ ] (N 11 mots) __/1

ABSTRACTION

Similitude entre ex: banane - orange = fruit [ ] marteau - tournevis [ ] allumette - lampe __/2

RAPPEL

Memory Index Score (MIS)

(MIS)	Doit se souvenir des mots SANS INDICE	JAMBE	COTON	ÉCOLE	TOMATE	BLANC	Points pour rappel SANS INDICE seulement
X3		[ ]	[ ]	[ ]	[ ]	[ ]	
X2	Indice de catégorie						
X1	Indice choix multiples						MIS = ___ /15

__/5

ORIENTATION

[ ] Date [ ] Mois [ ] Année [ ] Jour [ ] Endroit [ ] Ville __/6

© Z. Nasreddine MD www.mocatest.org (Normal ≥ 26/30)

Administré par: ____________________

Ajouter 1 point si scolarité ≤ 12 ans

TOTAL __/30

MIS = /15

Entraînement et certification requis pour assurer la précision.

INHANCE Trial

GDS - ENGLISH

Subject ID: ______________________

Administrator: ______________________

Date: ______________________

Timepoint: V0

Instructions:
Choose the best answer for how you felt **over the past week**.

No.	Questions	Answers
1	Are you basically satisfied with your life?	Yes No
2	Have you dropped many of your activities and interests?	Yes No
3	Do you feel that your life is empty?	Yes No
4	Do you often get bored?	Yes No
5	Are you in good spirits most of the time?	Yes No
6	Are you afraid that something bad is going to happen to you?	Yes No
7	Do you feel happy most of the time?	Yes No
8	Do you often feel helpless?	Yes No
9	Do you prefer to stay at home, rather than going out and doing new things?	Yes No
10	Do you feel you have more problems with memory than most?	Yes No
11	Do you think it is wonderful to be alive now?	Yes No
12	Do you feel pretty worthless the way you are now?	Yes No
13	Do you feel full of energy?	Yes No
14	Do you feel that your situation is hopeless?	Yes No
15	Do you think that most people are better off than you are?	Yes No

Essai INHANCE

EDG - FRANÇAIS

Sujet ID: ____________________

Administrateur: ____________________

Date: ____________________

Période de référence: V0

Instructions:
Choisissez la meilleure réponse pour décrire comment vous vous sentiez
au cours de la dernière semaine.

No.	Questions	Réponses
1	En général, êtes-vous satisfait de votre vie?	Oui Non
2	Avez-vous abandonné plusieurs de vos activités et intérêts?	Oui Non
3	Avez-vous l'impression que votre vie est vide?	Oui Non
4	Est-ce que vous vous ennuyez souvent?	Oui Non
5	Êtes-vous de bonne humeur la plupart du temps?	Oui Non
6	Avez-vous peur qu'un malheur vous arrive?	Oui Non
7	Vous sentez-vous heureux la plupart du temps?	Oui Non
8	Avez-vous souvent le sentiment d'être impuissant?	Oui Non
9	Préférez-vous rester à la maison plutôt que de sortir et faire de nouvelles expériences?	Oui Non
10	Avez-vous l'impression d'avoir plus de problèmes de mémoire que la majorité des gens?	Oui Non
11	Présentement, pensez-vous que la vie est merveilleuse?	Oui Non
12	Actuellement, avez-vous l'impression que vous ne valez rien?	Oui Non
13	Vous sentez-vous plein d'énergie?	Oui Non
14	Avez-vous le sentiment que votre situation est sans espoir?	Oui Non
15	Pensez-vous que la majorité des gens sont mieux que vous?	Oui Non

COLUMBIA-SUICIDE SEVERITY RATING SCALE (C-SSRS)

Since Last Visit

Version 1/14/09

Posner, K.; Brent, D.; Lucas, C.; Gould, M.; Stanley, B.; Brown, G.; Fisher, P.; Zelazny, J.; Burke, A.; Oquendo, M.; Mann, J.

Disclaimer:

This scale is intended to be used by individuals who have received training in its administration. The questions contained in the Columbia-Suicide Severity Rating Scale are suggested probes. Ultimately, the determination of the presence of suicidal ideation or behavior depends on the judgment of the individual administering the scale.

Definitions of behavioral suicidal events in this scale are based on those used in **The Columbia Suicide History Form**, *developed by John Mann, MD and Maria Oquendo, MD, Conte Center for the Neuroscience of Mental Disorders (CCNMD), New York State Psychiatric Institute, 1051 Riverside Drive, New York, NY, 10032. (Oquendo M. A., Halberstam B. & Mann J. J., Risk factors for suicidal behavior: utility and limitations of research instruments. In M.B. First [Ed.]* Standardized Evaluation in Clinical Practice, *pp. 103 -130, 2003.)*

For reprints of the C-SSRS contact Kelly Posner, Ph.D., New York State Psychiatric Institute, 1051 Riverside Drive, New York, New York, 10032; inquiries and training requirements contact posnerk@nyspi.columbia.edu

© 2008 The Research Foundation for Mental Hygiene, Inc.

**SUICIDAL IDEATION**	
Ask questions 1 and 2. If both are negative, proceed to "Suicidal Behavior" section. If the answer to question 2 is "yes", ask questions 3, 4 and 5. If the answer to question 1 and/or 2 is "yes", complete "Intensity of Ideation" section below.	**Since Last Visit**
**1. Wish to be Dead**<br>Subject endorses thoughts about a wish to be dead or not alive anymore, or wish to fall asleep and not wake up.<br>***Have you wished you were dead or wished you could go to sleep and not wake up?***<br><br>If yes, describe:	**Yes** ☐ **No** ☐
**2. Non-Specific Active Suicidal Thoughts**<br>General non-specific thoughts of wanting to end one's life/commit suicide (e.g., *"I've thought about killing myself"*) without thoughts of ways to kill oneself/associated methods, intent, or plan during the assessment period.<br>***Have you actually had any thoughts of killing yourself?***<br><br>If yes, describe:	**Yes** ☐ **No** ☐
**3. Active Suicidal Ideation with Any Methods (Not Plan) without Intent to Act**<br>Subject endorses thoughts of suicide and has thought of at least one method during the assessment period. This is different than a specific plan with time, place or method details worked out (e.g., thought of method to kill self but not a specific plan). Includes person who would say, *"I thought about taking an overdose but I never made a specific plan as to when, where or how I would actually do it…..and I would never go through with it"*.<br>***Have you been thinking about how you might do this?***<br><br>If yes, describe:	**Yes** ☐ **No** ☐
**4. Active Suicidal Ideation with Some Intent to Act, without Specific Plan**<br>Active suicidal thoughts of killing oneself and subject reports having some intent to act on such thoughts, as opposed to *"I have the thoughts but I definitely will not do anything about them"*.<br>***Have you had these thoughts and had some intention of acting on them?***<br><br>If yes, describe:	**Yes** ☐ **No** ☐
**5. Active Suicidal Ideation with Specific Plan and Intent**<br>Thoughts of killing oneself with details of plan fully or partially worked out and subject has some intent to carry it out.<br>***Have you started to work out or worked out the details of how to kill yourself? Do you intend to carry out this plan?***<br><br>If yes, describe:	**Yes** ☐ **No** ☐

**INTENSITY OF IDEATION**	
The following features should be rated with respect to the most severe type of ideation (i.e.,1-5 from above, with 1 being the least severe and 5 being the most severe).<br>***Most Severe Ideation:*** ________ ________________________________<br>***Type # (1-5)*** ***Description of Ideation***	Most Severe
**Frequency**<br>***How many times have you had these thoughts?***<br>(1) Less than once a week (2) Once a week (3) 2-5 times in week (4) Daily or almost daily (5) Many times each day	____
**Duration**<br>***When you have the thoughts how long do they last?***<br>(1) Fleeting - few seconds or minutes<br>(2) Less than 1 hour/some of the time<br>(3) 1-4 hours/a lot of time<br>(4) 4-8 hours/most of day<br>(5) More than 8 hours/persistent or continuous	____
**Controllability**<br>***Could/can you stop thinking about killing yourself or wanting to die if you want to?***<br>(1) Easily able to control thoughts<br>(2) Can control thoughts with little difficulty<br>(3) Can control thoughts with some difficulty<br>(4) Can control thoughts with a lot of difficulty<br>(5) Unable to control thoughts<br>(0) Does not attempt to control thoughts	____
**Deterrents**<br>***Are there things - anyone or anything (e.g., family, religion, pain of death) - that stopped you from wanting to die or acting on thoughts of committing suicide?***<br>(1) Deterrents definitely stopped you from attempting suicide<br>(2) Deterrents probably stopped you<br>(3) Uncertain that deterrents stopped you<br>(4) Deterrents most likely did not stop you<br>(5) Deterrents definitely did not stop you<br>(0) Does not apply	____
**Reasons for Ideation**<br>***What sort of reasons did you have for thinking about wanting to die or killing yourself? Was it to end the pain or stop the way you were feeling (in other words you couldn't go on living with this pain or how you were feeling) or was it to get attention, revenge or a reaction from others? Or both?***<br>(1) Completely to get attention, revenge or a reaction from others<br>(2) Mostly to get attention, revenge or a reaction from others<br>(3) Equally to get attention, revenge or a reaction from others and to end/stop the pain<br>(4) Mostly to end or stop the pain (you couldn't go on living with the pain or how you were feeling)<br>(5) Completely to end or stop the pain (you couldn't go on living with the pain or how you were feeling)<br>(0) Does not apply	____

SUICIDAL BEHAVIOR<br>*(Check all that apply, so long as these are separate events; must ask about all types)*	**Since Last Visit**
**Actual Attempt:**<br>A potentially self-injurious act committed with at least some wish to die, *as a result of act*. Behavior was in part thought of as method to kill oneself. Intent does not have to be 100%. If there is ***any*** intent/desire to die associated with the act, then it can be considered an actual suicide attempt. ***There does not have to be any injury or harm***, just the potential for injury or harm. If person pulls trigger while gun is in mouth but gun is broken so no injury results, this is considered an attempt.<br>Inferring Intent: Even if an individual denies intent/wish to die, it may be inferred clinically from the behavior or circumstances. For example, a highly lethal act that is clearly not an accident so no other intent but suicide can be inferred (e.g., gunshot to head, jumping from window of a high floor/story). Also, if someone denies intent to die, but they thought that what they did could be lethal, intent may be inferred.<br>***Have you made a suicide attempt?***<br>***Have you done anything to harm yourself?***<br>***Have you done anything dangerous where you could have died?***<br>***What did you do?***<br>***Did you______as a way to end your life?***<br>***Did you want to die (even a little) when you_____?***<br>***Were you trying to end your life when you_____?***<br>***Or Did you think it was possible you could have died from_____?***<br>***Or did you do it purely for other reasons / without ANY intention of killing yourself (like to relieve stress, feel better, get sympathy, or get something else to happen)?*** (Self-Injurious Behavior without suicidal intent)<br>If yes, describe:	**Yes** ☐ **No** ☐<br><br>Total # of Attempts<br>______
**Has subject engaged in Non-Suicidal Self-Injurious Behavior?**	**Yes** ☐ **No** ☐
**Interrupted Attempt:**<br>When the person is interrupted (by an outside circumstance) from starting the potentially self-injurious act *(if not for that, actual attempt would have occurred)*.<br>Overdose: Person has pills in hand but is stopped from ingesting. Once they ingest any pills, this becomes an attempt rather than an interrupted attempt. Shooting: Person has gun pointed toward self, gun is taken away by someone else, or is somehow prevented from pulling trigger. Once they pull the trigger, even if the gun fails to fire, it is an attempt. Jumping: Person is poised to jump, is grabbed and taken down from ledge. Hanging: Person has noose around neck but has not yet started to hang - is stopped from doing so.<br>***Has there been a time when you started to do something to end your life but someone or something stopped you before you actually did anything?***<br>If yes, describe:	**Yes** ☐ **No** ☐<br><br>Total # of interrupted<br>______
**Aborted Attempt:**<br>When person begins to take steps toward making a suicide attempt, but stops themselves before they actually have engaged in any self-destructive behavior. Examples are similar to interrupted attempts, except that the individual stops him/herself, instead of being stopped by something else.<br>***Has there been a time when you started to do something to try to end your life but you stopped yourself before you actually did anything?***<br>If yes, describe:	**Yes** ☐ **No** ☐<br>Total # of aborted<br>______
**Preparatory Acts or Behavior:**<br>Acts or preparation towards imminently making a suicide attempt. This can include anything beyond a verbalization or thought, such as assembling a specific method (e.g., buying pills, purchasing a gun) or preparing for one's death by suicide (e.g., giving things away, writing a suicide note).<br>***Have you taken any steps towards making a suicide attempt or preparing to kill yourself (such as collecting pills, getting a gun, giving valuables away or writing a suicide note)?***<br>If yes, describe:	**Yes** ☐ **No** ☐
**Suicidal Behavior:**<br>Suicidal behavior was present during the assessment period?	**Yes** ☐ **No** ☐
**Suicide:**	**Yes** ☐ **No** ☐
Answer for Actual Attempts Only	Most Lethal Attempt Date:
**Actual Lethality/Medical Damage:**<br>0. No physical damage or very minor physical damage (e.g., surface scratches).<br>1. Minor physical damage (e.g., lethargic speech; first-degree burns; mild bleeding; sprains).<br>2. Moderate physical damage; medical attention needed (e.g., conscious but sleepy, somewhat responsive; second-degree burns; bleeding of major vessel).<br>3. Moderately severe physical damage; *medical* hospitalization and likely intensive care required (e.g., comatose with reflexes intact; third-degree burns less than 20% of body; extensive blood loss but can recover; major fractures).<br>4. Severe physical damage; *medical* hospitalization with intensive care required (e.g., comatose without reflexes; third-degree burns over 20% of body; extensive blood loss with unstable vital signs; major damage to a vital area).<br>5. Death	*Enter Code*<br><br>______
**Potential Lethality: Only Answer if Actual Lethality=0**<br>Likely lethality of actual attempt if no medical damage (the following examples, while having no actual medical damage, had potential for very serious lethality: put gun in mouth and pulled the trigger but gun fails to fire so no medical damage; laying on train tracks with oncoming train but pulled away before run over).<br>0 = Behavior not likely to result in injury<br>1 = Behavior likely to result in injury but not likely to cause death<br>2 = Behavior likely to result in death despite available medical care	*Enter Code*<br><br>______

ÉCHELLE D'ÉVALUATION DE COLUMBIA SUR LE RISQUE SUICIDAIRE (C-SSRS)

Évaluation initiale et dépistage

Version du 14 janvier 2009

Posner, K.; Brent, D.; Lucas, C.; Gould, M.; Stanley, B.; Brown, G.; Fisher, P.; Zelazny, J.; Burke, A.; Oquendo, M.; Mann, J.

Avis d'exonération de responsabilité :

Cette échelle a été mise au point pour être utilisée par des personnes ayant été formées à son utilisation. Les questions contenues dans l'Échelle d'évaluation de Columbia sur le risque suicidaire (C-SSRS) sont des suggestions visant à obtenir des renseignements. En dernière analyse, la détermination de la présence d'idéations ou de comportements suicidaires dépend du jugement de la personne faisant passer le questionnaire.

Les définitions des comportements suicidaires dans cette échelle sont inspirées de celles utilisées dans **The Columbia Suicide History Form***, élaboré par John Mann, MD, et Maria Oquendo, MD, Conte Center for the Neuroscience of Mental Disorders (CCNMD), New York State Psychiatric Institute, 1051 Riverside Drive, New York, NY, 10032. (Oquendo M. A., Halberstam B. & Mann J. J., Risk factors for suicidal behavior: utility and limitations of research instruments. Dans M.B. First [Ed.]* Standardized Evaluation in Clinical Practice*, p. 103 -130, 2003.)*

Pour obtenir des exemplaires supplémentaires de l'échelle C-SSRS, veuillez communiquer avec Kelly Posner, Ph.D., New York State Psychiatric Institute, 1051 Riverside Drive, New York, New York, 10032; pour toute question et pour tout besoin en matière de formation, veuillez écrire à posnerk@nyspi.columbia.edu

© 2008 The Research Foundation for Mental Hygiene, Inc.

IDÉATION SUICIDAIRE		
Posez les questions 1 et 2. Si la réponse aux deux questions est négative, passez à la section « Comportement suicidaire ». Si la réponse à la question 2 est affirmative, posez les questions 3, 4 et 5. Si la réponse à la question 1 et/ou 2 est affirmative, remplissez la section « Intensité de l'idéation » ci-dessous.	**Depuis la naissance : moment où il/elle s'est senti(e) le plus suicidaire**	**Au cours des 2_ derniers mois**
**1. Désir d'être mort(e)**<br>Le sujet souscrit à des pensées concernant le désir d'être mort ou de ne plus être vivant, ou le désir de s'endormir et de ne pas se réveiller.<br>***Avez-vous déjà souhaité être mort(e) ou vous endormir et ne jamais vous réveiller?***<br><br>Dans l'affirmative, veuillez décrire :	**Oui** ▭ **Non** ▭	**Oui** ▭ **Non** ▭
**2. Pensées suicidaires actives non spécifiques**<br>Pensées générales non spécifiques au cours de la période d'évaluation concernant le désir de s'enlever la vie/se suicider (p. ex. : *« J'ai pensé à me suicider »*) sans penser à des moyens de se suicider ou à des méthodes, intentions ou plans connexes.<br>***Avez-vous réellement déjà pensé à vous suicider?***<br><br>Dans l'affirmative, veuillez décrire :	**Oui** ▭ **Non** ▭	**Oui** ▭ **Non** ▭
**3. Idéation suicidaire active avec définition de méthodes (pas de plan) et sans intention de passer à l'acte**<br>Le sujet confirme penser au suicide et a pensé à au moins une méthode au cours de la période d'évaluation. Ceci diffère d'un plan spécifique comprenant le moment, l'endroit ou une méthode détaillée (p. ex. : penser à une façon de se suicider, mais sans plan spécifique). Comprend les personnes qui diraient : *« J'ai pensé prendre une surdose de médicaments, mais je n'ai pas fait de plan spécifique concernant le moment ou la manière dont je m'y prendrais… et je ne pourrais jamais y arriver. »*<br>***Avez-vous pensé à comment vous vous y prendriez?***<br><br>Dans l'affirmative, veuillez décrire :	**Oui** ▭ **Non** ▭	**Oui** ▭ **Non** ▭
**4. Idéation suicidaire active avec une certaine intention de passer à l'acte, sans plan spécifique**<br>Le sujet a des pensées suicidaires actives et déclare avoir une certaine intention de passer à l'acte, comparativement à : *« J'y pense, mais je ne mettrai certainement pas ces pensées à exécution. »*<br>***Avez-vous eu ces pensées accompagnées d'une certaine intention de passer à l'acte?***<br><br>Dans l'affirmative, veuillez décrire :	**Oui** ▭ **Non** ▭	**Oui** ▭ **Non** ▭
**5. Idéation suicidaire active avec plan spécifique et intention de passer à l'acte**<br>Le sujet a des pensées suicidaires et un plan détaillé entièrement ou partiellement mis au point accompagné d'une certaine intention de mettre son plan à exécution.<br>***Avez-vous commencé à mettre au point ou avez-vous mis au point les détails de votre suicide? Avez-vous l'intention de mettre ce plan à exécution?***<br><br>Dans l'affirmative, veuillez décrire :	**Oui** ▭ **Non** ▭	**Oui** ▭ **Non** ▭

INTENSITÉ DE L'IDÉATION		
Les points suivants doivent être évalués en fonction du type d'idéation le plus grave (c.-à-d., à partir des idéations 1 à 5 ci-dessus, 1 étant la moins grave et 5 la plus grave). Demandez à quel moment il/elle s'est senti(e) le plus suicidaire.<br><br>Depuis la naissance - ***Idéation la plus grave :*** ________ ________________<br>***N° du type (1 à 5)*** ***Description de l'idéation***<br>Au cours des X derniers mois - ***Idéation la plus grave :*** ________ ________________<br>***N° du type (1 à 5)*** ***Description de l'idéation***	Idéation la plus grave	Idéation la plus grave
**Fréquence**<br>***Combien de fois avez-vous eu ces pensées?***<br>(1) Moins d'une fois par semaine (2) Une fois par semaine (3) De 2 à 5 fois par semaine (4) Tous les jours ou presque tous les jours (5) Plusieurs fois par jour	____	____
**Durée**<br>***Lorsque vous avez ces pensées, combien de temps durent-elles?***<br>(1) Brièvement – quelques secondes ou minutes<br>(2) Moins d'une heure / de temps à autre<br>(3) De 1 à 4 heures / souvent<br>(4) De 4 à 8 heures / presque toute la journée<br>(5) Plus de 8 heures / continuellement	____	____
**Contrôlabilité**<br>***Pourriez-vous ou pouvez-vous arrêter de penser au suicide ou à vouloir mourir si vous le souhaitez?***<br>(1) Facilement capable de maîtriser vos pensées.<br>(2) Vous pouvez maîtriser vos pensées avec peu de difficulté.<br>(3) Vous pouvez maîtriser vos pensées avec une certaine difficulté.<br>(4) Vous pouvez maîtriser vos pensées avec beaucoup de difficulté.<br>(5) Incapable de maîtriser vos pensées.<br>(0) Vous n'essayez pas de maîtriser vos pensées.	____	____
**Éléments dissuasifs**<br>***Y a-t-il des choses – des personnes ou quoi que ce soit d'autre (p. ex. : famille, religion, douleur liée à la mort) – qui vous ont empêché(e) de vouloir mourir ou de mettre vos pensées suicidaires à exécution?***<br>(1) Des éléments dissuasifs vous ont assurément empêché(e) de commettre une tentative de suicide.<br>(2) Des éléments dissuasifs vous ont probablement empêché(e) de commettre une tentative de suicide.<br>(3) Il n'est pas certain que des éléments dissuasifs vous aient empêché(e) de commettre une tentative de suicide.<br>(4) Il est probable que des éléments dissuasifs ne vous ont pas empêché(e) de commettre une tentative de suicide.<br>(5) Il est certain que des éléments dissuasifs ne vous ont pas empêché(e) de commettre une tentative de suicide.<br>(0) Sans objet.	____	____
**Motifs de l'idéation**<br>***Quelles sont les raisons pour avoir pensé à vouloir mourir ou à vous suicider? Était-ce pour faire cesser la douleur ou pour ne plus ressentir ce que vous ressentiez (autrement dit, vous ne pouviez plus continuer à vivre avec cette douleur ou dans cet état de mal-être), ou bien souhaitiez-vous attirer l'attention, vous venger ou susciter une réaction chez les autres? Ou tout ça en même temps?***<br>(1) Entièrement pour attirer l'attention, vous venger ou susciter une réaction chez les autres.<br>(2) Surtout pour attirer l'attention, vous venger ou susciter une réaction chez les autres.<br>(3) Autant pour attirer l'attention, vous venger ou susciter une réaction chez les autres que pour mettre fin à la douleur.<br>(4) Surtout pour mettre fin à la douleur (vous ne pouviez plus vivre avec la douleur ou dans cet état de mal-être).<br>(5) Entièrement pour mettre fin à la douleur (vous ne pouviez plus vivre avec la douleur ou dans cet état de mal-être).<br>(0) Sans objet.	____	____

© 2008 Research Foundation for Mental Hygiene, Inc.

**COMPORTEMENT SUICIDAIRE**<br>*(Cochez toutes les cases appropriées, pourvu qu'il s'agisse d'événements distincts; posez des questions sur tous les types de comportement.)*	**Depuis la naissance**	**Au cours des 2 dernières mois**
**Tentative réelle :**<br>Acte autoagressif potentiellement dommageable commis avec le désir plus ou moins fort de mourir, *à la suite de cet acte*. Ce comportement a été en partie envisagé comme moyen de se suicider. L'intention suicidaire ne doit pas être nécessairement de 100 %. Si une intention/volonté ***quelconque*** de mourir est associée à l'acte, celui-ci pourra alors être qualifié de tentative de suicide réelle. ***La présence de blessures ou de lésions n'est pas obligatoire***, le potentiel de blessure ou lésion suffit. Si la personne appuie sur la détente d'une arme à feu placée dans sa bouche, mais que l'arme est brisée et qu'aucune blessure n'est engendrée, cet acte est considéré comme étant une tentative.<br>Intention présumée : même si le sujet nie qu'il a l'intention ou le désir de mourir, son intention ou son désir peut être présumé cliniquement selon le comportement ou les circonstances. Par exemple, un acte pouvant entraîner une mort certaine et ne relevant clairement pas d'un accident, de sorte qu'il ne peut être assimilé qu'à une tentative de suicide (p. ex. : se tirer une balle dans la tête, sauter par la fenêtre d'un étage élevé). De plus, si la personne nie son intention de mourir, mais qu'elle croyait que ce qu'elle a fait pouvait être mortel, l'intention peut être présumée.<br>***Avez-vous fait une tentative de suicide?***<br>***Avez-vous fait quoi que ce soit pour vous blesser?***<br>***Avez-vous fait quelque chose de dangereux qui aurait pu causer votre mort?***<br>***Qu'avez-vous fait?***<br>***Avez-vous ______ dans le but de mettre fin à vos jours?***<br>***Vouliez-vous mourir (même un peu) lorsque vous ______?***<br>***Tentiez-vous de mettre fin à vos jours lorsque vous ______? ou***<br>***Pensiez-vous qu'il était possible que vous mouriez en ______?***<br>***Ou l'avez-vous fait purement pour d'autres raisons / sans AUCUNE intention de vous suicider (p. ex. : pour soulager le stress, vous sentir mieux, obtenir de la sympathie, ou pour que quelque chose d'autre se produise)?*** (Comportement autoagressif sans intention de suicide)<br>Dans l'affirmative, veuillez décrire :<br><br>**Le sujet a-t-il adopté un comportement autoagressif non suicidaire?**	**Oui** ☐ **Non** ☐<br><br>Nombre total de tentatives<br>____<br><br>**Oui** ☐ **Non** ☐	**Oui** ☐ **Non** ☐<br><br>Nombre total de tentatives<br>____<br><br>**Oui** ☐ **Non** ☐
**Tentative interrompue :**<br>Lorsque la personne est interrompue (par des circonstances externes) au moment où elle tente de poser le geste pouvant lui causer des blessures *(sans cette interruption, la tentative réelle aurait eu lieu)*.<br>Surdose : la personne a les pilules dans la main, mais on l'empêche de les avaler. Lorsque les pilules sont avalées, il s'agit d'une tentative plutôt que d'une tentative interrompue. Arme à feu : la personne a une arme à feu pointée sur elle-même, quelqu'un lui enlève son arme ou on l'empêche de quelque façon que ce soit d'appuyer sur la détente. Si la personne appuie sur la détente, même si le coup ne part pas, il s'agit d'une tentative. Saut : la personne s'apprête à sauter, mais quelqu'un l'attrape par derrière et l'éloigne du bord. Pendaison : la personne a la corde autour du cou, mais ne s'est pas encore pendue – quelqu'un l'en empêche.<br>***Y a-t-il eu un moment où vous aviez commencé à faire quelque chose pour mettre fin à vos jours, mais quelqu'un ou quelque chose vous a retenu avant que vous n'ayez fait quoi que ce soit?***<br>Dans l'affirmative, veuillez décrire :	**Oui** ☐ **Non** ☐<br><br>Nombre total de tentatives interrompues<br>____	**Oui** ☐ **Non** ☐<br><br>Nombre total de tentatives interrompues<br>____
**Tentative avortée :**<br>Lorsqu'une personne commence à prendre des mesures en vue de commettre une tentative de suicide, mais s'arrête d'elle-même avant d'adopter le comportement autodestructeur. Les exemples sont les mêmes que ceux pour les tentatives interrompues, sauf que le sujet s'arrête de lui-même au lieu d'être arrêté par quelque chose d'autre.<br>***Y a-t-il eu un moment où vous aviez commencé à faire quelque chose pour essayer de mettre fin à vos jours, mais vous vous êtes arrêté(e) de vous-même avant d'avoir fait quoi que ce soit?***<br>Dans l'affirmative, veuillez décrire :	**Oui** ☐ **Non** ☐<br><br>Nombre total de tentatives avortées<br>____	**Oui** ☐ **Non** ☐<br><br>Nombre total de tentatives avortées<br>____
**Actes ou comportements préparatoires :**<br>Actes ou préparatifs en vue de commettre une tentative imminente de suicide. Il peut s'agir de toute chose qui dépasse le stade de la verbalisation ou de la pensée, telle que réunir les éléments d'une méthode particulière (p. ex. : acheter des pilules, se procurer une arme à feu) ou se préparer à mourir par suicide (p. ex., donner ses biens, écrire une lettre de suicide).<br>***Avez-vous déjà pris des mesures en vue de faire une tentative de suicide ou vous préparer à vous suicider (p. ex. : vous procurer des pilules ou une arme à feu, donner vos biens ou écrire une lettre de suicide)?***<br>Dans l'affirmative, veuillez décrire :	**Oui** ☐ **Non** ☐	**Oui** ☐ **Non** ☐
**Comportement suicidaire :**<br>Comportement suicidaire présent au cours de la période d'évaluation?	**Oui** ☐ **Non** ☐	**Oui** ☐ **Non** ☐

Répondre en tenant compte des tentatives réelles seulement	Tentative la plus récente<br>Date :	Tentative la plus mortelle<br>Date :	Première tentative<br>Date :
**Degré des atteintes physiques observées :**<br>0. Aucune atteinte physique ou lésions très mineures (p. ex. : égratignures).<br>1. Atteinte physique mineure (p. ex. : élocution lente, brûlures au premier degré, léger saignement, entorse).<br>2. Atteinte physique modérée; soins médicaux nécessaires (p. ex. : état conscient mais somnolent, attention diffuse, brûlures au deuxième degré, saignement d'un vaisseau sanguin important).<br>3. Atteinte physique grave; hospitalisation nécessaire, probablement aux soins intensifs (p. ex. : état comateux avec réflexes intacts, brûlures au troisième degré sur moins de 20 % du corps, perte de sang abondante mais rétablissement probable, fractures importantes).<br>4. Atteinte physique très grave; hospitalisation aux soins intensifs nécessaire (p. ex. : état comateux sans réflexes, brûlures au troisième degré sur plus de 20 % du corps, perte de sang abondante avec signes vitaux instables, lésions importantes aux organes vitaux).<br>5. Décès	*Inscrire le code*<br>____	*Inscrire le code*<br>____	*Inscrire le code*<br>____
**Létalité potentielle : ne répondre que si la létalité réelle = 0**<br>Létalité probable de la tentative réelle en l'absence d'atteinte physique (exemples de tentatives n'ayant entraîné aucune atteinte physique, mais pouvant potentiellement engendrer un degré très élevé de létalité : placer le canon d'une arme à feu dans sa bouche et appuyer sur la détente, mais le coup ne part pas; s'étendre sur la voie ferrée à l'approche d'un train, mais en être retiré avant d'être écrasé).<br>0 = Peu de probabilité que le comportement aboutisse à des blessures.<br>1 = Probabilité que le comportement aboutisse à des blessures, mais peu de probabilité qu'il aboutisse à la mort.<br>2 = Probabilité que le comportement aboutisse à la mort malgré les soins médicaux disponibles.	*Inscrire le code*<br>____	*Inscrire le code*<br>____	*Inscrire le code*<br>____

© 2008 Research Foundation for Mental Hygiene, Inc.
