# Supplementary material for: Effects of Computerized Cognitive Training on Vesicular Acetylcholine Transporter Levels using [18F]Fluoroethoxybenzovesamicol Positron Emission Tomography in Healthy Older Adults: Results from the Improving Neurological Health in Aging via Neuroplasticity-based Computerized Exercise (INHANCE) Randomized Clinical Trial
Source: JMIR Serious Games. 2025 Oct 13;13:e75161. doi: 10.2196/75161 (PMC12559824; doi:10.2196/75161)
Supplement: Multimedia Appendix 4 [file games_v13i1e75161_app4.pdf]

Multimedia Appendix 4: Study flyers in English and French

Want to Stay Mentally Sharp?

Participants Needed for Study on Brain Health.

Our best hope to treat health conditions is through research. Did you know there is an urgent need for adults to participate? You CAN make a difference.

INHANCE

Who do we need?

1. Participants who are 65 or older without dementia
2. Speak fluent French or English
3. Can access the internet
4. Can receive MRI and PET scans
5. Can use a brain training program on a study-provided tablet

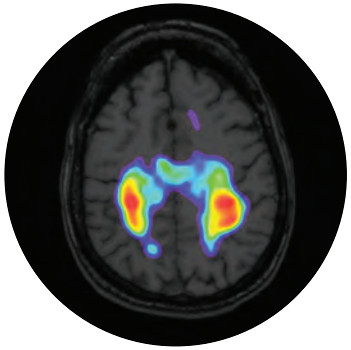

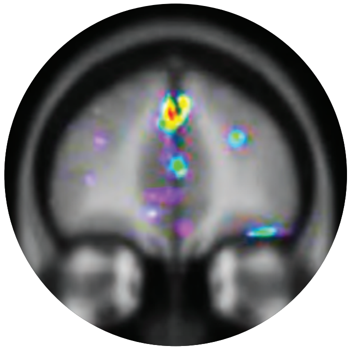

Researchers from McGill University will ask you to complete a computerized brain training program at home for 30 mins a day for 10 weeks. You will also be asked to visit our office in Montreal, Quebec to receive a complete overview of the study, cognitive assessments and brain imaging (PET, MRI) before and after your training program. You can be reimbursed up to $211 CAD for your participation.

NIH National Institute on Aging | McGill UNIVERSITY

Contact us if you would like to participate or learn more about the INHANCE brain study:

Martin Chevrier
514-398-2538

All information you provide will be confidential.

Voulez-vous rester mentalement vigilent?

À la recherche de participants pour une étude sur la santé du cerveau.

Notre meilleur espoir pour tratiter des problèmes de santé est avec la recherche. Savez-vous qu'il y a un besoin urgent pour la participation d'adultes? Vous POUVEZ faire une différence.

INHANCE

On a besoin de qui?

1. Participants de 65 ans et plus sans démence
2. Parle couramment le francais ou l'anglais
3. À accès internet
4. Peut subir des images IRM et PET
5. Peut utiliser un programme d'entraînement cognitif informatisé sur une tablette électronique d'étude

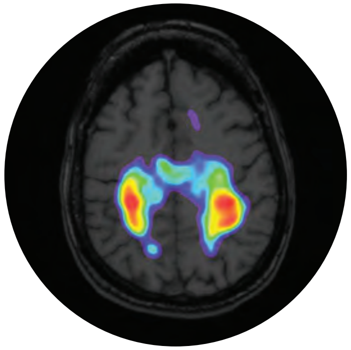

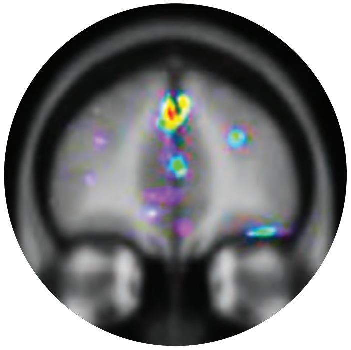

Des chercheurs de l'Université McGill vous demandront de compléter un programme d'entraînment cognitif informatisé à la maison qui durera 30 minutes par jour pour 10 semaines. Vous serez aussi demandés de nous visiter à nos bureaux à Montréal, Québec, pour recevoir une description complète de l'étude, des évaluations cognitives, et des images médicales (PET, IRM) avant et après le programme d'entraînement. Vous pouvez reçevoir un remboursement de 211$ pour votre participation

NIH National Institute on Aging

McGill UNIVERSITY

Contactez-nous si vous voulez participer ou en apprendre plus sur l'étude sur le cerveau INHANCE:

Luciano Buonamici
514-398-2801
luciano.buonamici@mcgill.ca

Tous les renseignements recueillis demeureront confidentiels.
