# Supplementary material for: Effects of Computerized Cognitive Training on Vesicular Acetylcholine Transporter Levels using [18F]Fluoroethoxybenzovesamicol Positron Emission Tomography in Healthy Older Adults: Results from the Improving Neurological Health in Aging via Neuroplasticity-based Computerized Exercise (INHANCE) Randomized Clinical Trial
Source: JMIR Serious Games. 2025 Oct 13;13:e75161. doi: 10.2196/75161 (PMC12559824; doi:10.2196/75161)
Supplement: Multimedia Appendix 5 [file games_v13i1e75161_app5.pdf]

CONSORT-EHEALTH (V 1.6.1) - Submission/Publication Form

The CONSORT-EHEALTH checklist is intended for authors of randomized trials evaluating web-based and Internet-based applications/interventions, including mobile interventions, electronic games (incl multiplayer games), social media, certain telehealth applications, and other interactive and/or networked electronic applications. Some of the items (e.g. all subitems under item 5 - description of the intervention) may also be applicable for other study designs.

The goal of the CONSORT EHEALTH checklist and guideline is to be
a) a guide for reporting for authors of RCTs,
b) to form a basis for appraisal of an ehealth trial (in terms of validity)

CONSORT-EHEALTH items/subitems are MANDATORY reporting items for studies published in the Journal of Medical Internet Research and other journals / scientific societies endorsing the checklist.

Items numbered 1., 2., 3., 4a., 4b etc are original CONSORT or CONSORT-NPT (non-pharmacologic treatment) items.
Items with Roman numerals (i., ii, iii, iv etc.) are CONSORT-EHEALTH extensions/clarifications.

As the CONSORT-EHEALTH checklist is still considered in a formative stage, we would ask that you also RATE ON A SCALE OF 1-5 how important/useful you feel each item is FOR THE PURPOSE OF THE CHECKLIST and reporting guideline (optional).

Mandatory reporting items are marked with a red *.
In the textboxes, either copy & paste the relevant sections from your manuscript into this form - please include any quotes from your manuscript in QUOTATION MARKS,
or answer directly by providing additional information not in the manuscript, or elaborating on why the item was not relevant for this study.

YOUR ANSWERS WILL BE PUBLISHED AS A SUPPLEMENTARY FILE TO YOUR PUBLICATION IN JMIR AND ARE CONSIDERED PART OF YOUR PUBLICATION (IF ACCEPTED).
Please fill in these questions diligently. Information will not be copyedited, so please use proper spelling and grammar, use correct capitalization, and avoid abbreviations.

DO NOT FORGET TO SAVE AS PDF _AND_ CLICK THE SUBMIT BUTTON SO YOUR ANSWERS ARE IN OUR DATABASE !!!

Citation Suggestion (if you append the pdf as Appendix we suggest to cite this paper in the caption):
Eysenbach G, CONSORT-EHEALTH Group

CONSORT-EHEALTH: Improving and Standardizing Evaluation Reports of Web-based and Mobile Health Interventions
J Med Internet Res 2011;13(4):e126
URL: http://www.jmir.org/2011/4/e126/
doi: 10.2196/jmir.1923
PMID: 22209829

mouna.attarha@positscience.com Switch account

Not shared

Draft saved

* Indicates required question

Your name *

First Last

Mouna Attarha

Primary Affiliation (short), City, Country *

University of Toronto, Toronto, Canada

Posit Science Corporation

Your e-mail address *

abc@gmail.com

Mouna.attarha@positscience.com

Title of your manuscript *

Provide the (draft) title of your manuscript.

Effects of Computerized Cognitive Training on Vesicular Acetylcholine Transporter Levels using [18F]Fluoroethoxybenzovesamicol Positron Emission Tomography in Healthy Older Adults: Results from the Improving Neurological Health in Aging via Neuroplasticity-based Computerized Exercise (INHANCE) Randomized Clinical Trial

Name of your App/Software/Intervention *

If there is a short and a long/alternate name, write the short name first and add the long name in brackets.

BrainHQ

Evaluated Version (if any)

e.g. "V1", "Release 2017-03-01", "Version 2.0.27913"

Your answer

Language(s) *

What language is the intervention/app in? If multiple languages are available, separate by comma (e.g. "English, French")

English, French

URL of your Intervention Website or App

e.g. a direct link to the mobile app on app in appstore (itunes, Google Play), or URL of the website. If the intervention is a DVD or hardware, you can also link to an Amazon page.

Your answer

URL of an image/screenshot (optional)

Your answer

Accessibility *

Can an enduser access the intervention presently?

- ○ access is free and open
- ○ access only for special usergroups, not open
- ◉ access is open to everyone, but requires payment/subscription/in-app purchases
- ○ app/intervention no longer accessible
- ○ Other:

Primary Medical Indication/Disease/Condition *

e.g. "Stress", "Diabetes", or define the target group in brackets after the condition, e.g. "Autism (Parents of children with)", "Alzheimers (Informal Caregivers of)"

Healthy adults

Primary Outcomes measured in trial *

comma-separated list of primary outcomes reported in the trial

pre/post FEOBV-PET SUVR in the anterior cing

Secondary/other outcomes

Are there any other outcomes the intervention is expected to affect?

Your answer

Recommended "Dose" *

What do the instructions for users say on how often the app should be used?

- (x) Approximately Daily
- ( ) Approximately Weekly
- ( ) Approximately Monthly
- ( ) Approximately Yearly
- ( ) "as needed"
- ( ) Other:

Approx. Percentage of Users (starters) still using the app as recommended after 3 months *

- (x) unknown / not evaluated
- ( ) 0-10%
- ( ) 11-20%
- ( ) 21-30%
- ( ) 31-40%
- ( ) 41-50%
- ( ) 51-60%
- ( ) 61-70%
- ( ) 71%-80%
- ( ) 81-90%
- ( ) 91-100%
- ( ) Other:

Overall, was the app/intervention effective? *

- (x) yes: all primary outcomes were significantly better in intervention group vs control
- ( ) partly: SOME primary outcomes were significantly better in intervention group vs control
- ( ) no statistically significant difference between control and intervention
- ( ) potentially harmful: control was significantly better than intervention in one or more outcomes
- ( ) inconclusive: more research is needed
- ( ) Other:

Article Preparation Status/Stage *

At which stage in your article preparation are you currently (at the time you fill in this form)

- ( ) not submitted yet - in early draft status
- ( ) not submitted yet - in late draft status, just before submission
- ( ) submitted to a journal but not reviewed yet
- ( ) submitted to a journal and after receiving initial reviewer comments
- (•) submitted to a journal and accepted, but not published yet
- ( ) published
- ( ) Other:

Journal *

If you already know where you will submit this paper (or if it is already submitted), please provide the journal name (if it is not JMIR, provide the journal name under "other")

- ( ) not submitted yet / unclear where I will submit this
- ( ) Journal of Medical Internet Research (JMIR)
- ( ) JMIR mHealth and UHealth
- (•) JMIR Serious Games
- ( ) JMIR Mental Health
- ( ) JMIR Public Health
- ( ) JMIR Formative Research
- ( ) Other JMIR sister journal
- ( ) Other:

Is this a full powered effectiveness trial or a pilot/feasibility trial? *

- ( ) Pilot/feasibility
- (•) Fully powered

Manuscript tracking number *

If this is a JMIR submission, please provide the manuscript tracking number under "other" (The ms tracking number can be found in the submission acknowledgement email, or when you login as author in JMIR. If the paper is already published in JMIR, then the ms tracking number is the four-digit number at the end of the DOI, to be found at the bottom of each published article in JMIR)

- ( ) no ms number (yet) / not (yet) submitted to / published in JMIR
- (•) Other: 75161

TITLE AND ABSTRACT

1a) TITLE: Identification as a randomized trial in the title

1a) Does your paper address CONSORT item 1a? *

I.e does the title contain the phrase "Randomized Controlled Trial"? (if not, explain the reason under "other")

- (•) yes
- ( ) Other:

1a-i) Identify the mode of delivery in the title

Identify the mode of delivery. Preferably use “web-based” and/or “mobile” and/or “electronic game” in the title. Avoid ambiguous terms like “online”, “virtual”, “interactive”. Use “Internet-based” only if Intervention includes non-web-based Internet components (e.g. email), use “computer-based” or “electronic” only if offline products are used. Use “virtual” only in the context of “virtual reality” (3-D worlds). Use “online” only in the context of “online support groups”. Complement or substitute product names with broader terms for the class of products (such as “mobile” or “smart phone” instead of “iphone”), especially if the application runs on different platforms.

	1	2	3	4	5	
subitem not at all important	○	○	○	○	○	essential

Does your paper address subitem 1a-i? *

Copy and paste relevant sections from manuscript title (include quotes in quotation marks "like this" to indicate direct quotes from your manuscript), or elaborate on this item by providing additional information not in the ms, or briefly explain why the item is not applicable/relevant for your study

"Both training programs required 35-hours of computerized training delivered over a 10-week period (approximately 30 minutes per session, 7 sessions per week, for a total of 70 sessions). Training was completed remotely at the participant’s home using either a loaned Android tablet or personal internet-connected device. Participants accessed their assigned program on an identical website (or app)"

1a-ii) Non-web-based components or important co-interventions in title

Mention non-web-based components or important co-interventions in title, if any (e.g., “with telephone support”).

	1	2	3	4	5	
subitem not at all important	○	○	○	○	○	essential

Does your paper address subitem 1a-ii?

Copy and paste relevant sections from manuscript title (include quotes in quotation marks "like this" to indicate direct quotes from your manuscript), or elaborate on this item by providing additional information not in the ms, or briefly explain why the item is not applicable/relevant for your study

Your answer

1a-iii) Primary condition or target group in the title

Mention primary condition or target group in the title, if any (e.g., "for children with Type I Diabetes") Example: A Web-based and Mobile Intervention with Telephone Support for Children with Type I Diabetes: Randomized Controlled Trial

	1	2	3	4	5	
subitem not at all important	○	○	○	○	○	essential

Does your paper address subitem 1a-iii? *

Copy and paste relevant sections from manuscript title (include quotes in quotation marks "like this" to indicate direct quotes from your manuscript), or elaborate on this item by providing additional information not in the ms, or briefly explain why the item is not applicable/relevant for your study

"community-dwelling healthy older adults aged 65 and above "

1b) ABSTRACT: Structured summary of trial design, methods, results, and conclusions

NPT extension: Description of experimental treatment, comparator, care providers, centers, and blinding status.

1b-i) Key features/functionalities/components of the intervention and comparator in the METHODS section of the ABSTRACT

Mention key features/functionalities/components of the intervention and comparator in the abstract. If possible, also mention theories and principles used for designing the site. Keep in mind the needs of systematic reviewers and indexers by including important synonyms. (Note: Only report in the abstract what the main paper is reporting. If this information is missing from the main body of text, consider adding it)

	1	2	3	4	5	
subitem not at all important	○	○	○	○	○	essential

Does your paper address subitem 1b-i? *

Copy and paste relevant sections from the manuscript abstract (include quotes in quotation marks "like this" to indicate direct quotes from your manuscript), or elaborate on this item by providing additional information not in the ms, or briefly explain why the item is not applicable/relevant for your study

"Participants were randomized at McGill University to an intervention of speed-based cognitive training exercises designed to improve the speed and accuracy of information processing or to an active control of non-speeded games designed for entertainment (e.g., similar in design to Solitaire). Participants completed 35 hours of training on their assigned program at home over a 10-week period using a loaned or personal internet-connected device. "

1b-ii) Level of human involvement in the METHODS section of the ABSTRACT

Clarify the level of human involvement in the abstract, e.g., use phrases like "fully automated" vs. "therapist/nurse/care provider/physician-assisted" (mention number and expertise of providers involved, if any). (Note: Only report in the abstract what the main paper is reporting. If this information is missing from the main body of text, consider adding it)

	1	2	3	4	5	
subitem not at all important	○	○	○	○	○	essential

Does your paper address subitem 1b-ii?

Copy and paste relevant sections from the manuscript abstract (include quotes in quotation marks "like this" to indicate direct quotes from your manuscript), or elaborate on this item by providing additional information not in the ms, or briefly explain why the item is not applicable/relevant for your study

Your answer

1b-iii) Open vs. closed, web-based (self-assessment) vs. face-to-face assessments in the METHODS section of the ABSTRACT

Mention how participants were recruited (online vs. offline), e.g., from an open access website or from a clinic or a closed online user group (closed usergroup trial), and clarify if this was a purely web-based trial, or there were face-to-face components (as part of the intervention or for assessment). Clearly say if outcomes were self-assessed through questionnaires (as common in web-based trials). Note: In traditional offline trials, an open trial (open-label trial) is a type of clinical trial in which both the researchers and participants know which treatment is being administered. To avoid confusion, use “blinded” or “unblinded” to indicated the level of blinding instead of “open”, as “open” in web-based trials usually refers to “open access” (i.e. participants can self-enrol). (Note: Only report in the abstract what the main paper is reporting. If this information is missing from the main body of text, consider adding it)

	1	2	3	4	5	
subitem not at all important	○	○	○	○	○	essential

Does your paper address subitem 1b-iii?

Copy and paste relevant sections from the manuscript abstract (include quotes in quotation marks "like this" to indicate direct quotes from your manuscript), or elaborate on this item by providing additional information not in the ms, or briefly explain why the item is not applicable/relevant for your study

Your answer

1b-iv) RESULTS section in abstract must contain use data

Report number of participants enrolled/assessed in each group, the use/uptake of the intervention (e.g., attrition/adherence metrics, use over time, number of logins etc.), in addition to primary/secondary outcomes. (Note: Only report in the abstract what the main paper is reporting. If this information is missing from the main body of text, consider adding it)

	1	2	3	4	5	
subitem not at all important	○	○	○	○	○	essential

Does your paper address subitem 1b-iv?

Copy and paste relevant sections from the manuscript abstract (include quotes in quotation marks "like this" to indicate direct quotes from your manuscript), or elaborate on this item by providing additional information not in the ms, or briefly explain why the item is not applicable/relevant for your study

Your answer

1b-v) CONCLUSIONS/DISCUSSION in abstract for negative trials

Conclusions/Discussions in abstract for negative trials: Discuss the primary outcome - if the trial is negative (primary outcome not changed), and the intervention was not used, discuss whether negative results are attributable to lack of uptake and discuss reasons. (Note: Only report in the abstract what the main paper is reporting. If this information is missing from the main body of text, consider adding it)

	1	2	3	4	5	
subitem not at all important	○	○	○	○	○	essential

Does your paper address subitem 1b-v?

Copy and paste relevant sections from the manuscript abstract (include quotes in quotation marks "like this" to indicate direct quotes from your manuscript), or elaborate on this item by providing additional information not in the ms, or briefly explain why the item is not applicable/relevant for your study

Your answer

INTRODUCTION

2a) In INTRODUCTION: Scientific background and explanation of rationale

2a-i) Problem and the type of system/solution

Describe the problem and the type of system/solution that is object of the study: intended as stand-alone intervention vs. incorporated in broader health care program? Intended for a particular patient population? Goals of the intervention, e.g., being more cost-effective to other interventions, replace or complement other solutions? (Note: Details about the intervention are provided in “Methods” under 5)

	1	2	3	4	5	
subitem not at all important	○	○	○	○	○	essential

Does your paper address subitem 2a-i? *

Copy and paste relevant sections from the manuscript (include quotes in quotation marks "like this" to indicate direct quotes from your manuscript), or elaborate on this item by providing additional information not in the ms, or briefly explain why the item is not applicable/relevant for your study

"low-risk intervention and contributes to the development of a mechanistic understanding of cognitive training that lays the groundwork for future trials"

2a-ii) Scientific background, rationale: What is known about the (type of) system

Scientific background, rationale: What is known about the (type of) system that is the object of the study (be sure to discuss the use of similar systems for other conditions/diagnoses, if appropiate), motivation for the study, i.e. what are the reasons for and what is the context for this specific study, from which stakeholder viewpoint is the study performed, potential impact of findings [2]. Briefly justify the choice of the comparator.

	1	2	3	4	5	
subitem not at all important	○	○	○	○	○	essential

Does your paper address subitem 2a-ii? *

Copy and paste relevant sections from the manuscript (include quotes in quotation marks "like this" to indicate direct quotes from your manuscript), or elaborate on this item by providing additional information not in the ms, or briefly explain why the item is not applicable/relevant for your study

"At the core of these network-based changes is synaptic plasticity, a fundamental process regulated by multiple neuromodulatory centers in the brain, including the basal forebrain which sends cholinergic projections across the forebrain.[4,5] These neuromodulatory systems in general, and cholinergic systems in particular, have long represented a potential driver of the benefits of computerized training programs and a novel mechanism of action by which these programs may show benefits to brain health and cognition. With the recent development of the FEOBV-PET method to quantitatively assess cholinergic system integrity in humans, and recent research that analogous forms of cognitive training for rodents altered cholinergic function,[75] we hypothesized that cognitive training exercises for humans (Double Decision and Freeze Frame) would affect cholinergic systems. Such a result would inform the neural mechanism of speed-based cognitive training programs, and open new avenues of research in the development of effective cognitive training programs."

2b) In INTRODUCTION: Specific objectives or hypotheses

Does your paper address CONSORT subitem 2b? *

Copy and paste relevant sections from the manuscript (include quotes in quotation marks "like this" to indicate direct quotes from your manuscript), or elaborate on this item by providing additional information not in the ms, or briefly explain why the item is not applicable/relevant for your study

"we hypothesized that cognitive training exercises for humans (Double Decision and Freeze Frame) would affect cholinergic systems. Such a result would inform the neural mechanism of speed-based cognitive training programs, and open new avenues of research in the development of effective cognitive training programs."

METHODS

3a) Description of trial design (such as parallel, factorial) including allocation ratio

Does your paper address CONSORT subitem 3a? *

Copy and paste relevant sections from the manuscript (include quotes in quotation marks "like this" to indicate direct quotes from your manuscript), or elaborate on this item by providing additional information not in the ms, or briefly explain why the item is not applicable/relevant for your study

"INHANCE is a prospective, double-blind, parallel-arm, active-controlled, randomized clinical trial in healthy adults aged 65 and above. " and "1:1 allocation to either the intervention or active control group"

3b) Important changes to methods after trial commencement (such as eligibility criteria), with reasons

Does your paper address CONSORT subitem 3b? *

Copy and paste relevant sections from the manuscript (include quotes in quotation marks "like this" to indicate direct quotes from your manuscript), or elaborate on this item by providing additional information not in the ms, or briefly explain why the item is not applicable/relevant for your study

"Enrollment spanned from July 2021 to December 2023. In October 2022, the protocol and recruitment flyer were modified to include “Potential participant must be able to communicate in either English or French” (originally “Potential participant must be a fluent English or French speaker from the age of 12”) as it unintentionally excluded minority groups from participation. This change to the inclusion criteria was approved by the study’s Data Safety Monitory Board, Western Institutional Review Board, and Research Ethics Board of McGill University Health Centre in Jan 2023."

3b-i) Bug fixes, Downtimes, Content Changes

Bug fixes, Downtimes, Content Changes: ehealth systems are often dynamic systems. A description of changes to methods therefore also includes important changes made on the intervention or comparator during the trial (e.g., major bug fixes or changes in the functionality or content) (5-iii) and other “unexpected events” that may have influenced study design such as staff changes, system failures/downtimes, etc. [2].

	1	2	3	4	5	
subitem not at all important	○	○	○	○	○	essential

Does your paper address subitem 3b-i?

Copy and paste relevant sections from the manuscript (include quotes in quotation marks "like this" to indicate direct quotes from your manuscript), or elaborate on this item by providing additional information not in the ms, or briefly explain why the item is not applicable/relevant for your study

Your answer

4a) Eligibility criteria for participants

Does your paper address CONSORT subitem 4a? *

Copy and paste relevant sections from the manuscript (include quotes in quotation marks "like this" to indicate direct quotes from your manuscript), or elaborate on this item by providing additional information not in the ms, or briefly explain why the item is not applicable/relevant for your study

"Training was completed remotely at the participant’s home using either a loaned Android tablet or personal internet-connected device." and "Once group assignment was determined, an automated email containing the participant’s group allocation was sent from LORIS to an unblinded team member at the site, who then conducted the program orientation visit with the participant."

4a-i) Computer / Internet literacy

Computer / Internet literacy is often an implicit “de facto” eligibility criterion - this should be explicitly clarified.

	1	2	3	4	5	
subitem not at all important	○	○	○	○	○	essential

Does your paper address subitem 4a-i?

Copy and paste relevant sections from the manuscript (include quotes in quotation marks "like this" to indicate direct quotes from your manuscript), or elaborate on this item by providing additional information not in the ms, or briefly explain why the item is not applicable/relevant for your study

Your answer

4a-ii) Open vs. closed, web-based vs. face-to-face assessments:

Open vs. closed, web-based vs. face-to-face assessments: Mention how participants were recruited (online vs. offline), e.g., from an open access website or from a clinic, and clarify if this was a purely web-based trial, or there were face-to-face components (as part of the intervention or for assessment), i.e., to what degree got the study team to know the participant. In online-only trials, clarify if participants were quasi-anonymous and whether having multiple identities was possible or whether technical or logistical measures (e.g., cookies, email confirmation, phone calls) were used to detect/prevent these.

	1	2	3	4	5	
subitem not at all important	○	○	○	○	○	essential

Does your paper address subitem 4a-ii? *

Copy and paste relevant sections from the manuscript (include quotes in quotation marks "like this" to indicate direct quotes from your manuscript), or elaborate on this item by providing additional information not in the ms, or briefly explain why the item is not applicable/relevant for your study

"All participants were recruited near McGill University, Canada, where the FEOBV radiotracer was synthesized and administered. Recruitment methods included public presentations (TV, radio stations, conferences), word of mouth, and flyers. Flyers were posted in churches, community centers, Facebook groups, local libraries, and at the neurology clinic at the Montreal Neurological Hospital (MNH). Flyers described the study and included information regarding inclusion/exclusion criteria as well as the study team's phone number and email address. Study flyers received ethics approval prior to distribution (see Multimedia Appendix 3: Study flyers in English and French)."

4a-iii) Information giving during recruitment

Information given during recruitment. Specify how participants were briefed for recruitment and in the informed consent procedures (e.g., publish the informed consent documentation as appendix, see also item X26), as this information may have an effect on user self-selection, user expectation and may also bias results.

	1	2	3	4	5	
subitem not at all important	○	○	○	○	○	essential

Does your paper address subitem 4a-iii?

Copy and paste relevant sections from the manuscript (include quotes in quotation marks "like this" to indicate direct quotes from your manuscript), or elaborate on this item by providing additional information not in the ms, or briefly explain why the item is not applicable/relevant for your study

Your answer

4b) Settings and locations where the data were collected

Does your paper address CONSORT subitem 4b? *

Copy and paste relevant sections from the manuscript (include quotes in quotation marks "like this" to indicate direct quotes from your manuscript), or elaborate on this item by providing additional information not in the ms, or briefly explain why the item is not applicable/relevant for your study

"All outcomes were measured in person by blinded assessors."

4b-i) Report if outcomes were (self-)assessed through online questionnaires

Clearly report if outcomes were (self-)assessed through online questionnaires (as common in web-based trials) or otherwise.

	1	2	3	4	5	
subitem not at all important	○	○	○	○	○	essential

Does your paper address subitem 4b-i? *

Copy and paste relevant sections from the manuscript (include quotes in quotation marks "like this" to indicate direct quotes from your manuscript), or elaborate on this item by providing additional information not in the ms, or briefly explain why the item is not applicable/relevant for your study

No outcomes were self-assessed through online questionnaires.

4b-ii) Report how institutional affiliations are displayed

Report how institutional affiliations are displayed to potential participants [on ehealth media], as affiliations with prestigious hospitals or universities may affect volunteer rates, use, and reactions with regards to an intervention.(Not a required item – describe only if this may bias results)

	1	2	3	4	5	
subitem not at all important	○	○	○	○	○	essential

Does your paper address subitem 4b-ii?

Copy and paste relevant sections from the manuscript (include quotes in quotation marks "like this" to indicate direct quotes from your manuscript), or elaborate on this item by providing additional information not in the ms, or briefly explain why the item is not applicable/relevant for your study

Your answer

5) The interventions for each group with sufficient details to allow replication, including how and when they were actually administered

5-i) Mention names, credential, affiliations of the developers, sponsors, and owners

Mention names, credential, affiliations of the developers, sponsors, and owners [6] (if authors/evaluators are owners or developer of the software, this needs to be declared in a “Conflict of interest” section or mentioned elsewhere in the manuscript).

	1	2	3	4	5	
subitem not at all important	○	○	○	○	○	essential

Does your paper address subitem 5-i?

Copy and paste relevant sections from the manuscript (include quotes in quotation marks "like this" to indicate direct quotes from your manuscript), or elaborate on this item by providing additional information not in the ms, or briefly explain why the item is not applicable/relevant for your study

Your answer

5-ii) Describe the history/development process

Describe the history/development process of the application and previous formative evaluations (e.g., focus groups, usability testing), as these will have an impact on adoption/use rates and help with interpreting results.

	1	2	3	4	5	
subitem not at all important	○	○	○	○	○	essential

Does your paper address subitem 5-ii?

Copy and paste relevant sections from the manuscript (include quotes in quotation marks "like this" to indicate direct quotes from your manuscript), or elaborate on this item by providing additional information not in the ms, or briefly explain why the item is not applicable/relevant for your study

Your answer

5-iii) Revisions and updating

Revisions and updating. Clearly mention the date and/or version number of the application/intervention (and comparator, if applicable) evaluated, or describe whether the intervention underwent major changes during the evaluation process, or whether the development and/or content was “frozen” during the trial. Describe dynamic components such as news feeds or changing content which may have an impact on the replicability of the intervention (for unexpected events see item 3b).

	1	2	3	4	5	
subitem not at all important	○	○	○	○	○	essential

Does your paper address subitem 5-iii?

Copy and paste relevant sections from the manuscript (include quotes in quotation marks "like this" to indicate direct quotes from your manuscript), or elaborate on this item by providing additional information not in the ms, or briefly explain why the item is not applicable/relevant for your study

Your answer

5-iv) Quality assurance methods

Provide information on quality assurance methods to ensure accuracy and quality of information provided [1], if applicable.

	1	2	3	4	5	
subitem not at all important	○	○	○	○	○	essential

Does your paper address subitem 5-iv?

Copy and paste relevant sections from the manuscript (include quotes in quotation marks "like this" to indicate direct quotes from your manuscript), or elaborate on this item by providing additional information not in the ms, or briefly explain why the item is not applicable/relevant for your study

Your answer

5-v) Ensure replicability by publishing the source code, and/or providing screenshots/screen-capture video, and/or providing flowcharts of the algorithms used

Ensure replicability by publishing the source code, and/or providing screenshots/screen-capture video, and/or providing flowcharts of the algorithms used. Replicability (i.e., other researchers should in principle be able to replicate the study) is a hallmark of scientific reporting.

	1	2	3	4	5	
subitem not at all important	○	○	○	○	○	essential

Does your paper address subitem 5-v?

Copy and paste relevant sections from the manuscript (include quotes in quotation marks "like this" to indicate direct quotes from your manuscript), or elaborate on this item by providing additional information not in the ms, or briefly explain why the item is not applicable/relevant for your study

Your answer

5-vi) Digital preservation

Digital preservation: Provide the URL of the application, but as the intervention is likely to change or disappear over the course of the years; also make sure the intervention is archived (Internet Archive, webcitation.org, and/or publishing the source code or screenshots/videos alongside the article). As pages behind login screens cannot be archived, consider creating demo pages which are accessible without login.

	1	2	3	4	5	
subitem not at all important	○	○	○	○	○	essential

Does your paper address subitem 5-vi?

Copy and paste relevant sections from the manuscript (include quotes in quotation marks "like this" to indicate direct quotes from your manuscript), or elaborate on this item by providing additional information not in the ms, or briefly explain why the item is not applicable/relevant for your study

Your answer

5-vii) Access

Access: Describe how participants accessed the application, in what setting/context, if they had to pay (or were paid) or not, whether they had to be a member of specific group. If known, describe how participants obtained "access to the platform and Internet" [1]. To ensure access for editors/reviewers/readers, consider to provide a "backdoor" login account or demo mode for reviewers/readers to explore the application (also important for archiving purposes, see vi).

	1	2	3	4	5	
subitem not at all important	○	○	○	○	○	essential

Does your paper address subitem 5-vii? *

Copy and paste relevant sections from the manuscript (include quotes in quotation marks "like this" to indicate direct quotes from your manuscript), or elaborate on this item by providing additional information not in the ms, or briefly explain why the item is not applicable/relevant for your study

Participants accessed the program on loaned or personal devices using the web or mobile app. Participants were paid: Participants received $30 USD (CAD $40) for completing the baseline visit which included PET and MRI imaging (V1), USD $10 (CAD $13) for every 10 training sessions completed during the intervention period (maximum of USD $70 [CAD $91] for completing all 70 sessions), USD $30 (CAD $40) for completing the post-test visit which included PET and MRI imaging (V2), and USD $30 (CAD $40) for the end-of-study 3-month follow-up visit (V3). Participants who completed all study activities were reimbursed USD $160 (CAD $211). Payment followed the completion of each visit.

5-viii) Mode of delivery, features/functionalities/components of the intervention and comparator, and the theoretical framework

Describe mode of delivery, features/functionalities/components of the intervention and comparator, and the theoretical framework [6] used to design them (instructional strategy [1], behaviour change techniques, persuasive features, etc., see e.g., [7, 8] for terminology). This includes an in-depth description of the content (including where it is coming from and who developed it) [1]," whether [and how] it is tailored to individual circumstances and allows users to track their progress and receive feedback" [6]. This also includes a description of communication delivery channels and – if computer-mediated communication is a component – whether communication was synchronous or asynchronous [6]. It also includes information on presentation strategies [1], including page design principles, average amount of text on pages, presence of hyperlinks to other resources, etc. [1].

	1	2	3	4	5	
subitem not at all important	○	○	○	○	○	essential

Does your paper address subitem 5-viii? *

Copy and paste relevant sections from the manuscript (include quotes in quotation marks "like this" to indicate direct quotes from your manuscript), or elaborate on this item by providing additional information not in the ms, or briefly explain why the item is not applicable/relevant for your study

Mode of delivery was via the app. Program components: "Participants received either visual speed training (the Double Decision and Freeze Frame exercises) or an active control of visual non-speeded computer games designed for entertainment (Double Klondike Solitaire, Bricks Breaking Hex). Both training programs required 35-hours of computerized training delivered over a 10-week period (approximately 30 minutes per session, 7 sessions per week, for a total of 70 sessions). Training was completed remotely at the participant's home using either a loaned Android tablet or personal internet-connected device. Participants accessed their assigned program on an identical website (or app) and were blinded to group assignment. See Figure 1.

The active control was designed to control for non-specific treatment effects, including placebo and practice effects, face-validity, interactions with research personnel, experience with computers, and was matched to the intervention in overall program use intensity, visual modality, platform delivery, delivery of rewards, and renumeration of time.[87–89] The control program was strategy-based and did not contain the active elements – intensive, adaptive training in the speed and accuracy of information processing accompanied by strong engagement of neuromodulatory systems – hypothesized to drive positive neuroplastic change in the intervention. Control games were developed by an independent gaming company and rated E (for everyone) by the Entertainment Software Rating Board".

Theoretical framework: "The design principles of these speeded cognitive training exercises are grounded in a theoretical framework discussed previously.[11,65,66] The neurobiological mechanisms underlying the generalized benefits seen with these forms of cognitive training however are largely unknown. Several pilot studies have offered initial insights, suggesting that biological mechanisms may include improved functional connectivity within the default mode and central executive networks, [67–69] improved brain synchronization,[70] improved functional magnetic resonance imaging (fMRI) and electroencephalography (EEG) measures of brain activation,[69–72] increased hippocampal activation,[73] and improved diffusion tensor imaging measure of white-matter insulation between brain regions dedicated to visual and attentional processing.[74]

At the core of these network-based changes is synaptic plasticity, a fundamental process regulated by multiple neuromodulatory centers in the brain, including the basal forebrain which sends cholinergic projections across the forebrain.[4,5] These neuromodulatory systems in general, and cholinergic systems in particular, have long represented a potential driver of the benefits of computerized training programs and a novel mechanism of action by which these programs may show benefits to brain health and cognition. With the recent development of the FEOBV-PET method to quantitatively assess cholinergic system integrity in humans, and recent research that analogous forms of cognitive training for rodents altered cholinergic function,[75] we hypothesized that cognitive training exercises for humans (Double Decision and Freeze Frame) would affect cholinergic systems. Such a result would inform the neural mechanism of speed-based cognitive training programs, and open new avenues of research in the development of effective cognitive training programs.

"

5-ix) Describe use parameters

Describe use parameters (e.g., intended “doses” and optimal timing for use). Clarify what instructions or recommendations were given to the user, e.g., regarding timing, frequency, heaviness of use, if any, or was the intervention used ad libitum.

	1	2	3	4	5	
subitem not at all important	○	○	○	○	○	essential

Does your paper address subitem 5-ix?

Copy and paste relevant sections from the manuscript (include quotes in quotation marks "like this" to indicate direct quotes from your manuscript), or elaborate on this item by providing additional information not in the ms, or briefly explain why the item is not applicable/relevant for your study

Your answer

5-x) Clarify the level of human involvement

Clarify the level of human involvement (care providers or health professionals, also technical assistance) in the e-intervention or as co-intervention (detail number and expertise of professionals involved, if any, as well as “type of assistance offered, the timing and frequency of the support, how it is initiated, and the medium by which the assistance is delivered”. It may be necessary to distinguish between the level of human involvement required for the trial, and the level of human involvement required for a routine application outside of a RCT setting (discuss under item 21 – generalizability).

	1	2	3	4	5	
subitem not at all important	○	○	○	○	○	essential

Does your paper address subitem 5-x?

Copy and paste relevant sections from the manuscript (include quotes in quotation marks "like this" to indicate direct quotes from your manuscript), or elaborate on this item by providing additional information not in the ms, or briefly explain why the item is not applicable/relevant for your study

Your answer

5-xi) Report any prompts/reminders used

Report any prompts/reminders used: Clarify if there were prompts (letters, emails, phone calls, SMS) to use the application, what triggered them, frequency etc. It may be necessary to distinguish between the level of prompts/reminders required for the trial, and the level of prompts/reminders for a routine application outside of a RCT setting (discuss under item 21 – generalizability).

	1	2	3	4	5	
subitem not at all important	○	○	○	○	○	essential

Does your paper address subitem 5-xi? *

Copy and paste relevant sections from the manuscript (include quotes in quotation marks "like this" to indicate direct quotes from your manuscript), or elaborate on this item by providing additional information not in the ms, or briefly explain why the item is not applicable/relevant for your study

Study staff conducted weekly check-ins over the phone or email to provide training reminders, answer participant questions, and troubleshoot issues.

5-xii) Describe any co-interventions (incl. training/support)

Describe any co-interventions (incl. training/support): Clearly state any interventions that are provided in addition to the targeted eHealth intervention, as ehealth intervention may not be designed as stand-alone intervention. This includes training sessions and support [1]. It may be necessary to distinguish between the level of training required for the trial, and the level of training for a routine application outside of a RCT setting (discuss under item 21 – generalizability.

	1	2	3	4	5	
subitem not at all important	○	○	○	○	○	essential

Does your paper address subitem 5-xii? *

Copy and paste relevant sections from the manuscript (include quotes in quotation marks "like this" to indicate direct quotes from your manuscript), or elaborate on this item by providing additional information not in the ms, or briefly explain why the item is not applicable/relevant for your study

Study staff conducted weekly check-ins over the phone or email in both the intervention and control group. There were no other interventional components aside from speed training and nonspeeded training.

6a) Completely defined pre-specified primary and secondary outcome measures, including how and when they were assessed

Does your paper address CONSORT subitem 6a? *

Copy and paste relevant sections from the manuscript (include quotes in quotation marks "like this" to indicate direct quotes from your manuscript), or elaborate on this item by providing additional information not in the ms, or briefly explain why the item is not applicable/relevant for your study

"The primary outcome was FEOBV binding using mean standard uptake value ratios (SUVR) in the primary ROI, the anterior cingulate cortex, between baseline and post-test for the ITT population.
FEOBV binding was also assessed in pre-specified exploratory ROIs, including global cortex (frontal lobe, parietal lobe, occipital lobe, temporal lobe), hippocampus, parahippocampal gyrus, striatum (putamen and caudate), posterior cingulate cortex, primary auditory cortex, primary sensorimotor cortex and in the Nucleus Basalis of Meynert (nbM) which provides the major source of cholinergic innervation to the cerebral cortex. In the nbM, FEOBV SUVR likely represents a combination of tracer binding to cholinergic cell bodies and cholinergic brainstem afferents terminating there.
A structural MRI (3T Siemens Magnetom Prisma, 20 minutes) was acquired during the first scanning session to co-register the PET (Siemens High-Resolution Research Tomograph). (See Multimedia Appendix 4: PET and MRI acquisition and data analysis). Cognitive control was measured by performance on three computerized sub-tests (Flanker, Set-Shifting, and Anti-Saccades) completed on a computer from a validated neuropsychological battery (z-score of the executive composite from NIH EXAMINER[90], 20 minutes). Participants also completed two train-to-task cognitive assessments (Double Decision assessment and Freeze Frame assessment, 15 minutes) modeled directly upon the two training exercises in the intervention (z-scores) to evaluate target engagement, and behavioral measures sensitive to acetylcholine function (heart-rate variability using a Shimmer 4-lead Consensys ECG Development Kit, and pupillometry using Tobii Pro Glasses 2). These cognitive and behavioral assessments were completed at baseline, post-test, and at a 3-month no-contact follow-up. See the protocol for additional details.[79]"

6a-i) Online questionnaires: describe if they were validated for online use and apply CHERRIES items to describe how the questionnaires were designed/deployed

If outcomes were obtained through online questionnaires, describe if they were validated for online use and apply CHERRIES items to describe how the questionnaires were designed/deployed [9].

	1	2	3	4	5	
subitem not at all important	○	○	○	○	○	essential

Does your paper address subitem 6a-i?

Copy and paste relevant sections from manuscript text

Your answer

6a-ii) Describe whether and how “use” (including intensity of use/dosage) was defined/measured/monitored

Describe whether and how “use” (including intensity of use/dosage) was defined/measured/monitored (logins, logfile analysis, etc.). Use/adoption metrics are important process outcomes that should be reported in any ehealth trial.

	1	2	3	4	5	
subitem not at all important	○	○	○	○	○	essential

Does your paper address subitem 6a-ii?

Copy and paste relevant sections from manuscript text

Your answer

6a-iii) Describe whether, how, and when qualitative feedback from participants was obtained

Describe whether, how, and when qualitative feedback from participants was obtained (e.g., through emails, feedback forms, interviews, focus groups).

	1	2	3	4	5	
subitem not at all important	○	○	○	○	○	essential

Does your paper address subitem 6a-iii?

Copy and paste relevant sections from manuscript text

Your answer

6b) Any changes to trial outcomes after the trial commenced, with reasons

Does your paper address CONSORT subitem 6b? *

Copy and paste relevant sections from the manuscript (include quotes in quotation marks "like this" to indicate direct quotes from your manuscript), or elaborate on this item by providing additional information not in the ms, or briefly explain why the item is not applicable/relevant for your study

There were no changes to trial outcomes after the trial commenced.

7a) How sample size was determined

NPT: When applicable, details of whether and how the clustering by care provides or centers was addressed

7a-i) Describe whether and how expected attrition was taken into account when calculating the sample size

Describe whether and how expected attrition was taken into account when calculating the sample size.

	1	2	3	4	5	
subitem not at all important	○	○	○	○	○	essential

Does your paper address subitem 7a-i?

Copy and paste relevant sections from manuscript title (include quotes in quotation marks "like this" to indicate direct quotes from your manuscript), or elaborate on this item by providing additional information not in the ms, or briefly explain why the item is not applicable/relevant for your study

Your answer

7b) When applicable, explanation of any interim analyses and stopping guidelines

Does your paper address CONSORT subitem 7b? *

Copy and paste relevant sections from the manuscript (include quotes in quotation marks "like this" to indicate direct quotes from your manuscript), or elaborate on this item by providing additional information not in the ms, or briefly explain why the item is not applicable/relevant for your study

"No interim analyses or stopping guidelines were defined."

8a) Method used to generate the random allocation sequence

NPT: When applicable, how care providers were allocated to each trial group

Does your paper address CONSORT subitem 8a? *

Copy and paste relevant sections from the manuscript (include quotes in quotation marks "like this" to indicate direct quotes from your manuscript), or elaborate on this item by providing additional information not in the ms, or briefly explain why the item is not applicable/relevant for your study

"minimization method of adaptive stratified randomization"

8b) Type of randomisation; details of any restriction (such as blocking and block size)

Does your paper address CONSORT subitem 8b? *

Copy and paste relevant sections from the manuscript (include quotes in quotation marks "like this" to indicate direct quotes from your manuscript), or elaborate on this item by providing additional information not in the ms, or briefly explain why the item is not applicable/relevant for your study

"We employed a minimization method of adaptive stratified randomization with a 1:1 allocation to either the intervention or active control group. Groups were stratified based on baseline FEOBV and the baseline NIH EXAMINER composite score."

9) Mechanism used to implement the random allocation sequence (such as sequentially numbered containers), describing any steps taken to conceal the sequence until interventions were assigned

Does your paper address CONSORT subitem 9? *

Copy and paste relevant sections from the manuscript (include quotes in quotation marks "like this" to indicate direct quotes from your manuscript), or elaborate on this item by providing additional information not in the ms, or briefly explain why the item is not applicable/relevant for your study

The minimization method addresses the general problem of ensuring stratification across multiple factors in small- or moderate-sized trials. The various imbalances are added together to give the overall imbalance in the study. Group assignment is then made at random with a heavy probability weighting (0.8) in favor of the group that would minimize imbalance. Out of 2, 1 participant (50%) will be assigned to the intervention group, and 1 participant (50%) will be assigned to the active control group. Participant allocation was performed after the baseline visit (V1) and before the planned first day of program use. Baseline data were fully monitored for each participant, with all queries resolved, because allocation depended on obtaining data for the 2 prognostic variables.

10) Who generated the random allocation sequence, who enrolled participants, and who assigned participants to interventions

Does your paper address CONSORT subitem 10? *

Copy and paste relevant sections from the manuscript (include quotes in quotation marks "like this" to indicate direct quotes from your manuscript), or elaborate on this item by providing additional information not in the ms, or briefly explain why the item is not applicable/relevant for your study

"Following the completion of baseline assessments, a blinded team member from the study site initiated a randomization request by entering both scores and setting the randomization status to “Awaiting Review” in LORIS, a data collection and management system with strict data access controls.[86] This request was then directed to an unblinded team member at the coordinating center who reviewed and confirmed the accuracy of the participant’s scores and set the randomization status to “Reviewed” which executed an automated script. The script cumulatively assessed the overall imbalance in the study. The final group assignment was made at random, with a strong probability weighting (0.8) favoring the group that would minimize the imbalance. Once group assignment was determined, an automated email containing the participant’s group allocation was sent from LORIS to an unblinded team member at the site, who then conducted the program orientation visit with the participant. All randomizations occurred before the participant’s first day of program use."

11a) If done, who was blinded after assignment to interventions (for example, participants, care providers, those assessing outcomes) and how

NPT: Whether or not administering co-interventions were blinded to group assignment

11a-i) Specify who was blinded, and who wasn’t

Specify who was blinded, and who wasn’t. Usually, in web-based trials it is not possible to blind the participants [1, 3] (this should be clearly acknowledged), but it may be possible to blind outcome assessors, those doing data analysis or those administering co-interventions (if any).

	1	2	3	4	5	
subitem not at all important	○	○	○	○	○	essential

Does your paper address subitem 11a-i? *

Copy and paste relevant sections from the manuscript (include quotes in quotation marks "like this" to indicate direct quotes from your manuscript), or elaborate on this item by providing additional information not in the ms, or briefly explain why the item is not applicable/relevant for your study

Unblinded study staff will provide support for participants using their assigned programs. Only blinded study staff are authorized to participate in assessment administration, scoring, evaluation, follow-up of study participants after randomization, and baseline data analyses. The principal investigators and site investigator are required to complete a Delegation of Authority Form before the start of the study indicating which activities individual site research team members will be authorized to complete. To prevent unblinding, the following controls occurred at the site level: (1) the cognitive training condition and the active control condition will be identified as “Treatment A” and “Treatment B”; (2) participants will be instructed and reminded not to discuss details related to their training program with site staff, colleagues, friends, or acquaintances; (3) principal investigators will be instructed to not discuss details of either treatment arm with site personnel; (4) the site will be required to minimize the possibility of accidental unblinding of the site staff (eg, unintended viewing of treatment sessions); and (5) signage will be posted in appropriate areas throughout the facility reminding participants to not discuss study details."

11a-ii) Discuss e.g., whether participants knew which intervention was the “intervention of interest” and which one was the “comparator”

Informed consent procedures (4a-ii) can create biases and certain expectations - discuss e.g., whether participants knew which intervention was the “intervention of interest” and which one was the “comparator”.

	1	2	3	4	5	
subitem not at all important	○	○	○	○	○	essential

Does your paper address subitem 11a-ii?

Copy and paste relevant sections from the manuscript (include quotes in quotation marks "like this" to indicate direct quotes from your manuscript), or elaborate on this item by providing additional information not in the ms, or briefly explain why the item is not applicable/relevant for your study

Your answer

11b) If relevant, description of the similarity of interventions

(this item is usually not relevant for ehealth trials as it refers to similarity of a placebo or sham intervention to a active medication/intervention)

Does your paper address CONSORT subitem 11b? *

Copy and paste relevant sections from the manuscript (include quotes in quotation marks "like this" to indicate direct quotes from your manuscript), or elaborate on this item by providing additional information not in the ms, or briefly explain why the item is not applicable/relevant for your study

"The active control was designed to control for non-specific treatment effects, including placebo and practice effects, face-validity, interactions with research personnel, experience with computers, and was matched to the intervention in overall program use intensity, visual modality, platform delivery, delivery of rewards, and renumeration of time.[87–89] The control program was strategy-based and did not contain the active elements – intensive, adaptive training in the speed and accuracy of information processing accompanied by strong engagement of neuromodulatory systems – hypothesized to drive positive neuroplastic change in the intervention."

12a) Statistical methods used to compare groups for primary and secondary outcomes

NPT: When applicable, details of whether and how the clustering by care providers or centers was addressed

Does your paper address CONSORT subitem 12a? *

Copy and paste relevant sections from the manuscript (include quotes in quotation marks "like this" to indicate direct quotes from your manuscript), or elaborate on this item by providing additional information not in the ms, or briefly explain why the item is not applicable/relevant for your study

"The primary statistical analysis methodology was a linear mixed model approach. The intervention and active control groups in the ITT population were compared at baseline to identify potential covariates.[91] The data for each outcome measure was examined by using a linear mixed effects model with treatment group and time as fixed factors, and covariates as necessary from the baseline analysis. Within-group effects for each time point (post-training, follow-up) were calculated using data from each group. Between-groups effects for each time point were calculated in the same way, adding an interaction term (Training group x Time) to estimate the effect of cognitive training on outcome measure change. The analyses performed in this study were consistent with those outlined in the published protocol"

12a-i) Imputation techniques to deal with attrition / missing values

Imputation techniques to deal with attrition / missing values: Not all participants will use the intervention/comparator as intended and attrition is typically high in ehealth trials. Specify how participants who did not use the application or dropped out from the trial were treated in the statistical analysis (a complete case analysis is strongly discouraged, and simple imputation techniques such as LOCF may also be problematic [4]).

	1	2	3	4	5	
subitem not at all important	○	○	○	○	○	essential

Does your paper address subitem 12a-i? *

Copy and paste relevant sections from the manuscript (include quotes in quotation marks "like this" to indicate direct quotes from your manuscript), or elaborate on this item by providing additional information not in the ms, or briefly explain why the item is not applicable/relevant for your study

"The ITT was defined a priori as participants who were randomized and completed at least one session of training post-randomization (~30 minutes)." and "The statistical model used iterative full-information maximum likelihood estimation to account for missing data."

12b) Methods for additional analyses, such as subgroup analyses and adjusted analyses

Does your paper address CONSORT subitem 12b? *

Copy and paste relevant sections from the manuscript (include quotes in quotation marks "like this" to indicate direct quotes from your manuscript), or elaborate on this item by providing additional information not in the ms, or briefly explain why the item is not applicable/relevant for your study

"The analyses performed in this study were consistent with those outlined in the published protocol, except for additional analyses suggested by anonymous reviewers of related papers on the INHANCE trial, including the baseline cognition subgroup analysis." and "We explored the effect of baseline cognition using the MoCA with “low cognition” defined as a MoCA total score of 23–26[80] and “high cognition” defined as a MoCA total score of 27–30." Subgroup analyses evaluated high/low cognition on the primary and exploratory outcomes using the same linear mixed model approach used for the primary outcome."

X26) REB/IRB Approval and Ethical Considerations [recommended as subheading under "Methods"] (not a CONSORT item)

X26-i) Comment on ethics committee approval

	1	2	3	4	5	
subitem not at all important	○	○	○	○	○	essential

Does your paper address subitem X26-i?

Copy and paste relevant sections from the manuscript (include quotes in quotation marks "like this" to indicate direct quotes from your manuscript), or elaborate on this item by providing additional information not in the ms, or briefly explain why the item is not applicable/relevant for your study

Your answer

x26-ii) Outline informed consent procedures

Outline informed consent procedures e.g., if consent was obtained offline or online (how? Checkbox, etc.?), and what information was provided (see 4a-ii). See [6] for some items to be included in informed consent documents.

	1	2	3	4	5	
subitem not at all important	○	○	○	○	○	essential

Does your paper address subitem X26-ii?

Copy and paste relevant sections from the manuscript (include quotes in quotation marks "like this" to indicate direct quotes from your manuscript), or elaborate on this item by providing additional information not in the ms, or briefly explain why the item is not applicable/relevant for your study

Your answer

X26-iii) Safety and security procedures

Safety and security procedures, incl. privacy considerations, and any steps taken to reduce the likelihood or detection of harm (e.g., education and training, availability of a hotline)

	1	2	3	4	5	
subitem not at all important	○	○	○	○	○	essential

Does your paper address subitem X26-iii?

Copy and paste relevant sections from the manuscript (include quotes in quotation marks "like this" to indicate direct quotes from your manuscript), or elaborate on this item by providing additional information not in the ms, or briefly explain why the item is not applicable/relevant for your study

Your answer

RESULTS

13a) For each group, the numbers of participants who were randomly assigned, received intended treatment, and were analysed for the primary outcome

NPT: The number of care providers or centers performing the intervention in each group and the number of patients treated by each care provider in each center

Does your paper address CONSORT subitem 13a? *

Copy and paste relevant sections from the manuscript (include quotes in quotation marks "like this" to indicate direct quotes from your manuscript), or elaborate on this item by providing additional information not in the ms, or briefly explain why the item is not applicable/relevant for your study

"The data from the ITT population (N=92) included 46 participants each in the intervention and active control arms and were analyzed."

13b) For each group, losses and exclusions after randomisation, together with reasons

Does your paper address CONSORT subitem 13b? (NOTE: Preferably, this is shown in a CONSORT flow diagram) *

Copy and paste relevant sections from the manuscript (include quotes in quotation marks "like this" to indicate direct quotes from your manuscript), or elaborate on this item by providing additional information not in the ms, or briefly explain why the item is not applicable/relevant for your study

"A total of 113 individuals were screened and provided written informed consent; of these, 20 did not qualify or wish to participate further." Please see the attrition diagram for reasons.

13b-i) Attrition diagram

Strongly recommended: An attrition diagram (e.g., proportion of participants still logging in or using the intervention/comparator in each group plotted over time, similar to a survival curve) or other figures or tables demonstrating usage/dose/engagement.

	1	2	3	4	5	
subitem not at all important	○	○	○	○	○	essential

Does your paper address subitem 13b-i?

Copy and paste relevant sections from the manuscript or cite the figure number if applicable (include quotes in quotation marks "like this" to indicate direct quotes from your manuscript), or elaborate on this item by providing additional information not in the ms, or briefly explain why the item is not applicable/relevant for your study

Your answer

14a) Dates defining the periods of recruitment and follow-up

Does your paper address CONSORT subitem 14a? *

Copy and paste relevant sections from the manuscript (include quotes in quotation marks "like this" to indicate direct quotes from your manuscript), or elaborate on this item by providing additional information not in the ms, or briefly explain why the item is not applicable/relevant for your study

"enrolled July 2021 to December 2023; final follow-up June 2024"

14a-i) Indicate if critical “secular events” fell into the study period

Indicate if critical “secular events” fell into the study period, e.g., significant changes in Internet resources available or “changes in computer hardware or Internet delivery resources”

	1	2	3	4	5	
subitem not at all important	○	○	○	○	○	essential

Does your paper address subitem 14a-i?

Copy and paste relevant sections from the manuscript (include quotes in quotation marks "like this" to indicate direct quotes from your manuscript), or elaborate on this item by providing additional information not in the ms, or briefly explain why the item is not applicable/relevant for your study

Your answer

14b) Why the trial ended or was stopped (early)

Does your paper address CONSORT subitem 14b? *

Copy and paste relevant sections from the manuscript (include quotes in quotation marks "like this" to indicate direct quotes from your manuscript), or elaborate on this item by providing additional information not in the ms, or briefly explain why the item is not applicable/relevant for your study

The N defined by the power analysis was met.

15) A table showing baseline demographic and clinical characteristics for each group

NPT: When applicable, a description of care providers (case volume, qualification, expertise, etc.) and centers (volume) in each group

Does your paper address CONSORT subitem 15? *

Copy and paste relevant sections from the manuscript (include quotes in quotation marks "like this" to indicate direct quotes from your manuscript), or elaborate on this item by providing additional information not in the ms, or briefly explain why the item is not applicable/relevant for your study

Please see the baseline characteristics table. And "Participant characteristics for the ITT are presented in Table 1. Baseline characteristics were well-balanced between the intervention and active control groups and differed statistically only for the baseline Double Decision assessment which was used as a covariate in the model, t(90) = -2.073, P = .04. In general, participants were cognitively normal without depression, college-educated, and identified predominantly as female and white. Additional baseline characteristics of the sample were reported previously.[92]
Participants randomized to the intervention (N=46) demonstrated a mean NIH EXAMINER executive composite score of 0.38 (SD = 0.64). The mean raw Double Decision train-to-task assessment score was 1632.46 milliseconds (SD = 883.24) with lower scores indicating better performance. The mean raw Freeze Frame train-to-task assessment score was 4.22 (SD = 1.99), with higher scores indicating better performance.
Participants randomized to the active control (N=46) had a NIH EXAMINER executive composite score of 0.49 (SD = 0.50), a Double Decision train-to-task assessment score of 1320.93 milliseconds (SD = 712.12) and a Freeze Frame train-to-task assessment score of 5.11 (SD = 2.09)."

15-i) Report demographics associated with digital divide issues

In ehealth trials it is particularly important to report demographics associated with digital divide issues, such as age, education, gender, social-economic status, computer/Internet/ehealth literacy of the participants, if known.

	1	2	3	4	5	
subitem not at all important	○	○	○	○	○	essential

Does your paper address subitem 15-i? *

Copy and paste relevant sections from the manuscript (include quotes in quotation marks "like this" to indicate direct quotes from your manuscript), or elaborate on this item by providing additional information not in the ms, or briefly explain why the item is not applicable/relevant for your study

Participants without computers or mobile devices received a loaned device to participate in the trial. All participants received an orientation to the loaned device. All participants received weekly check-ins to troubleshoot technical issues and have questions answered.

16) For each group, number of participants (denominator) included in each analysis and whether the analysis was by original assigned groups

16-i) Report multiple “denominators” and provide definitions

Report multiple “denominators” and provide definitions: Report N’s (and effect sizes) “across a range of study participation [and use] thresholds” [1], e.g., N exposed, N consented, N used more than x times, N used more than y weeks, N participants “used” the intervention/comparator at specific pre-defined time points of interest (in absolute and relative numbers per group). Always clearly define “use” of the intervention.

	1	2	3	4	5	
subitem not at all important	○	○	○	○	○	essential

Does your paper address subitem 16-i? *

Copy and paste relevant sections from the manuscript (include quotes in quotation marks "like this" to indicate direct quotes from your manuscript), or elaborate on this item by providing additional information not in the ms, or briefly explain why the item is not applicable/relevant for your study

The analysis was based on original assigned groups and there were 41 participants in both groups who composed the intent-to-treat population (ITT). Data from the ITT were analyzed.

16-ii) Primary analysis should be intent-to-treat

Primary analysis should be intent-to-treat, secondary analyses could include comparing only “users”, with the appropriate caveats that this is no longer a randomized sample (see 18-i).

	1	2	3	4	5	
subitem not at all important	○	○	○	○	○	essential

Does your paper address subitem 16-ii?

Copy and paste relevant sections from the manuscript (include quotes in quotation marks "like this" to indicate direct quotes from your manuscript), or elaborate on this item by providing additional information not in the ms, or briefly explain why the item is not applicable/relevant for your study

Your answer

17a) For each primary and secondary outcome, results for each group, and the estimated effect size and its precision (such as 95% confidence interval)

Does your paper address CONSORT subitem 17a? *

Copy and paste relevant sections from the manuscript (include quotes in quotation marks "like this" to indicate direct quotes from your manuscript), or elaborate on this item by providing additional information not in the ms, or briefly explain why the item is not applicable/relevant for your study

"Table 2 presents FEOBV-PET SUVRs in the primary ROI, anterior cingulate cortex, for the intervention and active control at baseline and post-test. There was a significant within-group effect of the speeded intervention on SUVR before and after training (change mean: +0.044, 95% confidence interval (CI): 0.006 to 0.082, P = 0.028, medium effect size (ω^2) = 0.09). There was no within-group effect for the non-speeded active control (change mean: +0.014, 95% CI: -0.026 to 0.054, P = 0.498, effect size (ω^2) < 0.01)."

17a-i) Presentation of process outcomes such as metrics of use and intensity of use

In addition to primary/secondary (clinical) outcomes, the presentation of process outcomes such as metrics of use and intensity of use (dose, exposure) and their operational definitions is critical. This does not only refer to metrics of attrition (13-b) (often a binary variable), but also to more continuous exposure metrics such as “average session length”. These must be accompanied by a technical description how a metric like a “session” is defined (e.g., timeout after idle time) [1] (report under item 6a).

	1	2	3	4	5	
subitem not at all important	○	○	○	○	○	essential

Does your paper address subitem 17a-i?

Copy and paste relevant sections from the manuscript (include quotes in quotation marks "like this" to indicate direct quotes from your manuscript), or elaborate on this item by providing additional information not in the ms, or briefly explain why the item is not applicable/relevant for your study

Your answer

17b) For binary outcomes, presentation of both absolute and relative effect sizes is recommended

Does your paper address CONSORT subitem 17b? *

Copy and paste relevant sections from the manuscript (include quotes in quotation marks "like this" to indicate direct quotes from your manuscript), or elaborate on this item by providing additional information not in the ms, or briefly explain why the item is not applicable/relevant for your study

The trial did not have binary outcomes. There was one primary outcome and the remaining outcomes were exploratory.

18) Results of any other analyses performed, including subgroup analyses and adjusted analyses, distinguishing pre-specified from exploratory

Does your paper address CONSORT subitem 18? *

Copy and paste relevant sections from the manuscript (include quotes in quotation marks "like this" to indicate direct quotes from your manuscript), or elaborate on this item by providing additional information not in the ms, or briefly explain why the item is not applicable/relevant for your study

"Table 2 presents FEOBV-PET SUVR in exploratory ROIs for the intervention and active control at baseline, baseline vs post-test, and baseline vs follow-up. These analyses showed significant within-group effects for the speeded intervention in brain regions associated with memory including the hippocampus (change mean: +0.089, 95% CI: 0.015 to 0.163, P = 0.024, medium effect size (ω²) = 0.10) and the parahippocampal gyrus (change mean: +0.059, 95% CI: 0.005 to 0.114, P = 0.039, medium effect size (ω²) = 0.08). There was a non-significant medium effect size for the temporal lobe (change mean: +0.043, 95% CI: -0.002 to 0.089, P = 0.069, medium effect size (ω²) = 0.06). There were non-significant small effect sizes for global cortex (change mean: +0.026, 95% CI: -0.007 to 0.059, P = 0.135, small effect size (ω²) = 0.03), frontal lobe (change mean: +0.033, 95% CI: -0.013 to 0.080, P = 0.166, small effect size (ω²) = 0.02), occipital lobe (change mean: +0.033, 95% CI: -0.005 to 0.072, P = 0.099, small effect size (ω²) = 0.04), (change mean: +0.086, 95% CI: -0.026 to 0.197, P = 0.137, small effect size (ω²) = 0.03).

There were no within-group effects of FEOBV SUVR for the active control across the exploratory ROIs. There were no within-group effects for the speeded intervention for the parietal lobe, striatum, putamen, caudate, posterior cingulate, primary auditory cortex, or primary sensorimotor cortex. There were no between-group effects across the exploratory ROIs.

The trial administered three exploratory cognitive measures at baseline, post-test, and follow-up (Figure 5). Performance on the cognitive control subtests of the NIH EXAMINER battery (Flanker, Set-Shifting, and Anti-Saccades) showed no within-group or between-group effects at post-test or follow-up. In contrast, the Double Decision and Freeze Frame assessments, which evaluated target engagement, showed significant effects. Speeded training led to a significant between-group effect on the Double Decision train-to-task assessment (speeded vs non-speeded z-score change difference: +1.53, 95% CI: 1.073 to 1.989, P < 0.001, large effect size (ω²) = 0.32) and on the Freeze Frame train-to-task assessment (speeded vs non-speeded z-score change difference: +0.903, 95% CI: 0.477 to 1.327, P < 0.001, large effect size (ω²) = 0.16) at post-test. These effects were sustained at the 3-month no-contact follow-up for both Double Decision (speeded vs non-speeded z-score change difference: +1.177, 95% CI: 0.787 to 1.570, P < 0.001, large effect size (ω²) = 0.28) and Freeze Frame (speeded vs non-speeded z-score change difference: +0.893, 95% CI: 0.508 to 1.277, P < 0.001, large effect size (ω²) = 0.19). These findings confirm that the training successfully engaged the target and that improvements from a brief, intensive training period are maintained after training concludes.

We explored the effect of baseline cognition using the MoCA with "low cognition" defined as a MoCA total score of 23–26[80] (N=51, 55% of the ITT) and "high cognition" defined as a MoCA total score of 27–30 (N=41, 45% of the ITT). The low cognition subgroup had a mean executive composite score of 0.09 (SD = .62) on NIH EXAMINER at baseline which was notably lower than the mean composite of 0.65 (SD =.53) in the high cognition subgroup. There was a within-group effect of speed training on NIH EXAMINER for the low cognition group (composite z-score change mean: +0.194, 95% CI: 0.042 to 0.351, P = 0.021, large effect size (ω²) = 0.21) but not the high cognition group (composite z-score change mean: -0.038, 95% CI: -0.230 to 0.151, P = 0.688, effect size (ω²) < 0.01), with no within-group effect for the active control for either the low (composite z-score change mean: +0.064, 95% CI: -0.092 to 0.223, P = 0.425, effect size (ω²) < 0.01) or high subgroups (composite z-score change mean: +0.036, 95% CI: -0.187 to 0.259, P = 0.748, effect size (ω²) < 0.01). See Figure 6A.

The effect of speed training was maintained at the 3-month no-contact follow-up showing [illegible]

sustained benefits to cognition after training (composite z-score change mean: +0.211, 95% CI: 0.064 to 0.365, P = 0.011, large effect size (ω^2) = 0.26). The active control also showed cognitive improvement at the 3-month no-contact follow-up relative to baseline (composite z-score change mean: +0.157, 95% CI: 0.009 to 0.308, P = 0.046, medium effect size (ω^2) = 0.11). Given that no significant cognitive improvement was observed immediately post-training, this does not reflect a maintenance effect and may be interpreted as either a practice effect on the NIH EXAMINER due to repeated administrations over time, or as a delayed cognitive benefit emerging from engagement with the control game training.
In the subgroup with high baseline cognition, there was a significant between-groups effect favoring the speeded intervention in the primary ROI (speeded vs non-speeded SUVR change difference: +0.081, 95% CI: 0.036 to 0.125, P = 0.001, large effect size (ω^2) = 0.24). See Figure 6B. On a within-group basis, the intervention showed a significant increase in binding between baseline and post-test (change mean: +0.050, 95% CI: 0.016 to 0.082, P = 0.007, small effect size (ω^2) = 0.03) while the active control showed a significant decline in binding between baseline and post-test (change mean: -0.031, 95% CI: 0.060 to -0.002, P = 0.047, large effect size (ω^2) = 0.18). In the subgroup with low baseline cognition, there was no between-group (speeded vs non-speeded SUVR change difference: -0.006, 95% CI: -0.100 to 0.088, P=0.902, effect size (ω^2) < 0.01) or within-group effects for the intervention (change mean: +0.040, 95% CI: -0.032 to 0.112, P = 0.282, small effect size (ω^2) = 0.01) or control (change mean: +0.046, 95% CI: -0.019 to 0.109, P = 0.168, small effect size (ω^2) = 0.04)."

18-i) Subgroup analysis of comparing only users

A subgroup analysis of comparing only users is not uncommon in ehealth trials, but if done, it must be stressed that this is a self-selected sample and no longer an unbiased sample from a randomized trial (see 16-iii).

	1	2	3	4	5	
subitem not at all important	○	○	○	○	○	essential

Does your paper address subitem 18-i?

Copy and paste relevant sections from the manuscript (include quotes in quotation marks "like this" to indicate direct quotes from your manuscript), or elaborate on this item by providing additional information not in the ms, or briefly explain why the item is not applicable/relevant for your study

Your answer

19) All important harms or unintended effects in each group
(for specific guidance see CONSORT for harms)

Does your paper address CONSORT subitem 19? *

Copy and paste relevant sections from the manuscript (include quotes in quotation marks "like this" to indicate direct quotes from your manuscript), or elaborate on this item by providing additional information not in the ms, or briefly explain why the item is not applicable/relevant for your study

"Participants reported a total of 54 adverse events of which 4 (7.41%) were related to the administration of the radioligand. Of those 4 reports, one participant from the active control reported 2 events (mild dry mouth, mild strange sensations in the mouth) and a second participant who was not randomized reported 2 events (moderate nausea, moderate vomiting). Both recovered without treatment. No adverse events were attributed to the training programs and no serious adverse events or deaths were reported."

19-i) Include privacy breaches, technical problems

Include privacy breaches, technical problems. This does not only include physical “harm” to participants, but also incidents such as perceived or real privacy breaches [1], technical problems, and other unexpected/unintended incidents. “Unintended effects” also includes unintended positive effects [2].

	1	2	3	4	5	
subitem not at all important	○	○	○	○	○	essential

Does your paper address subitem 19-i?

Copy and paste relevant sections from the manuscript (include quotes in quotation marks "like this" to indicate direct quotes from your manuscript), or elaborate on this item by providing additional information not in the ms, or briefly explain why the item is not applicable/relevant for your study

Your answer

19-ii) Include qualitative feedback from participants or observations from staff/researchers

Include qualitative feedback from participants or observations from staff/researchers, if available, on strengths and shortcomings of the application, especially if they point to unintended/unexpected effects or uses. This includes (if available) reasons for why people did or did not use the application as intended by the developers.

	1	2	3	4	5	
subitem not at all important	○	○	○	○	○	essential

Does your paper address subitem 19-ii?

Copy and paste relevant sections from the manuscript (include quotes in quotation marks "like this" to indicate direct quotes from your manuscript), or elaborate on this item by providing additional information not in the ms, or briefly explain why the item is not applicable/relevant for your study

Your answer

DISCUSSION

22) Interpretation consistent with results, balancing benefits and harms, and considering other relevant evidence

NPT: In addition, take into account the choice of the comparator, lack of or partial blinding, and unequal expertise of care providers or centers in each group

22-i) Restate study questions and summarize the answers suggested by the data, starting with primary outcomes and process outcomes (use)

Restate study questions and summarize the answers suggested by the data, starting with primary outcomes and process outcomes (use).

	1	2	3	4	5	
subitem not at all important	○	○	○	○	○	essential

Does your paper address subitem 22-i? *

Copy and paste relevant sections from the manuscript (include quotes in quotation marks "like this" to indicate direct quotes from your manuscript), or elaborate on this item by providing additional information not in the ms, or briefly explain why the item is not applicable/relevant for your study

"Age-related declines in neuromodulatory functioning are associated with declines in cognition. In this double-blind randomized controlled trial, community-dwelling healthy older adults aged 65 and above were randomized to one of two computerized cognitive training programs: speeded training or non-speeded training. FEOBV-PET was used to investigate whether cholinergic function can be modified by these training programs. In the largest trial using FEOBV-PET to date, we demonstrate for the first time in humans that speed training significantly improves cholinergic function in the anterior cingulate cortex, the primary region of interest. The 2.3% increase in FEOBV SUVR binding achieved over the 10-week intervention period within the anterior cingulate is comparable to the estimated decline of 2.5% in FEOBV SUVR binding[29] observed over each decade of aging from the age of 20. The cholinergic system has major projections from the basal forebrain to the cortex and hippocampus. Exploratory analyses showed SUVR increases in the hippocampus and parahippocampal gyrus - regions known to be particularly vulnerable to age-related declines in cholinergic terminal integrity.[95] This finding suggests potential enhancements in memory-related processes, which may help explain the benefits of speed training on memory reported in previous studies.[96] The well-known ACTIVE trial[97] for example, showed that healthy older adults who completed 10-18 hours of training on Double Decision experienced a 29-48% reduction in dementia risk a decade later.[43] This risk reduction in dementia incidence could reflect the long-term benefit of improving cholinergic health through speed training."

22-ii) Highlight unanswered new questions, suggest future research

Highlight unanswered new questions, suggest future research.

	1	2	3	4	5	
subitem not at all important	○	○	○	○	○	essential

Does your paper address subitem 22-ii?

Copy and paste relevant sections from the manuscript (include quotes in quotation marks "like this" to indicate direct quotes from your manuscript), or elaborate on this item by providing additional information not in the ms, or briefly explain why the item is not applicable/relevant for your study

Your answer

20) Trial limitations, addressing sources of potential bias, imprecision, and, if relevant, multiplicity of analyses

20-i) Typical limitations in ehealth trials

Typical limitations in ehealth trials: Participants in ehealth trials are rarely blinded. Ehealth trials often look at a multiplicity of outcomes, increasing risk for a Type I error. Discuss biases due to non-use of the intervention/usability issues, biases through informed consent procedures, unexpected events.

	1	2	3	4	5	
subitem not at all important	○	○	○	○	○	essential

Does your paper address subitem 20-i? *

Copy and paste relevant sections from the manuscript (include quotes in quotation marks "like this" to indicate direct quotes from your manuscript), or elaborate on this item by providing additional information not in the ms, or briefly explain why the item is not applicable/relevant for your study

"The limited racial and ethnic diversity among trial participants restricts the generalizability of the findings to non-white populations. To increase minority representation over the course of the trial, extensive outreach efforts were made. We contacted 20 community centers throughout Montreal with greater minority representation, 200+ churches, 10 local libraries, and completed several media interviews about INHANCE. Despite these and other efforts, the majority of the sample identified as white. Barriers to robust minority representation in clinical trials may include mistrust and skepticism due to historical trial abuses in medical research, time and resource constraints, cultural and language barriers, discomfort with the research process, and the perception of greater personal risk. With respect to INHANCE, the use of a radioligand and the commitment to 35 hours of training may have further deterred minority participation.[124–127] Future studies should reduce the burden of study-related activities and implement equitable research practices.[128]
This mechanism paper was motivated by the positive cognitive and functional benefits observed in healthy older adults who were randomized to speed training in the ACTIVE trial. Although we aimed to enroll healthy older adults, the lower MoCA cutoff of 23[84] may have inadvertently included participants with undiagnosed mild cognitive impairment, introducing a source of variance into the current trial.
Finally, only known confounders of cognitive training reported in the literature (e.g., major depression) were specified in the published protocol. Potential confounders influencing cholinergic plasticity such as smoking or the use of systemic estrogens via hormone-replacement therapy were not considered in the current study."

21) Generalisability (external validity, applicability) of the trial findings

NPT: External validity of the trial findings according to the intervention, comparators, patients, and care providers or centers involved in the trial

21-i) Generalizability to other populations

Generalizability to other populations: In particular, discuss generalizability to a general Internet population, outside of a RCT setting, and general patient population, including applicability of the study results for other organizations

	1	2	3	4	5	
subitem not at all important	○	○	○	○	○	essential

Does your paper address subitem 21-i?

Copy and paste relevant sections from the manuscript (include quotes in quotation marks "like this" to indicate direct quotes from your manuscript), or elaborate on this item by providing additional information not in the ms, or briefly explain why the item is not applicable/relevant for your study

Your answer

21-ii) Discuss if there were elements in the RCT that would be different in a routine application setting

Discuss if there were elements in the RCT that would be different in a routine application setting (e.g., prompts/reminders, more human involvement, training sessions or other co-interventions) and what impact the omission of these elements could have on use, adoption, or outcomes if the intervention is applied outside of a RCT setting.

	1	2	3	4	5	
subitem not at all important	○	○	○	○	○	essential

Does your paper address subitem 21-ii?

Copy and paste relevant sections from the manuscript (include quotes in quotation marks "like this" to indicate direct quotes from your manuscript), or elaborate on this item by providing additional information not in the ms, or briefly explain why the item is not applicable/relevant for your study

Your answer

OTHER INFORMATION

23) Registration number and name of trial registry

Does your paper address CONSORT subitem 23? *

Copy and paste relevant sections from the manuscript (include quotes in quotation marks "like this" to indicate direct quotes from your manuscript), or elaborate on this item by providing additional information not in the ms, or briefly explain why the item is not applicable/relevant for your study

NCT04149457 and “Improving Neurological Health in Aging Via Neuroplasticity-based Computerized Exercise (INHANCE)”

24) Where the full trial protocol can be accessed, if available

Does your paper address CONSORT subitem 24? *

Cite a Multimedia Appendix, other reference, or copy and paste relevant sections from the manuscript (include quotes in quotation marks "like this" to indicate direct quotes from your manuscript), or elaborate on this item by providing additional information not in the ms, or briefly explain why the item is not applicable/relevant for your study

Attarha M, Pelegrino AC de F, Toussaint PJ, Grant SJ, Vleet TV, Villers-Sidani E de. Improving Neurological Health in Aging Via Neuroplasticity-Based Computerized Exercise: Protocol for a Randomized Controlled Trial. JMIR Research Protocols. 2024;13(1):e59705. doi:10.2196/59705

25) Sources of funding and other support (such as supply of drugs), role of funders

Does your paper address CONSORT subitem 25? *

Copy and paste relevant sections from the manuscript (include quotes in quotation marks "like this" to indicate direct quotes from your manuscript), or elaborate on this item by providing additional information not in the ms, or briefly explain why the item is not applicable/relevant for your study

"Research reported in this publication was supported by the National Institute on Aging of the National Institutes of Health under Award Numbers R44AG039965 and 3R44AG039965-06S1 awarded to MA and TVV."

X27) Conflicts of Interest (not a CONSORT item)

X27-i) State the relation of the study team towards the system being evaluated

In addition to the usual declaration of interests (financial or otherwise), also state the relation of the study team towards the system being evaluated, i.e., state if the authors/evaluators are distinct from or identical with the developers/sponsors of the intervention.

	1	2	3	4	5	
subitem not at all important	○	○	○	○	○	essential

Does your paper address subitem X27-i?

Copy and paste relevant sections from the manuscript (include quotes in quotation marks "like this" to indicate direct quotes from your manuscript), or elaborate on this item by providing additional information not in the ms, or briefly explain why the item is not applicable/relevant for your study

Your answer

About the CONSORT EHEALTH checklist

As a result of using this checklist, did you make changes in your manuscript? *

- ○ yes, major changes
- ○ yes, minor changes
- ◉ no

What were the most important changes you made as a result of using this checklist?

Your answer

How much time did you spend on going through the checklist INCLUDING making changes in your manuscript *

I probably spent around 1.5 hours

As a result of using this checklist, do you think your manuscript has improved? *

- ○ yes
- ◉ no
- ○ Other:

Would you like to become involved in the CONSORT EHEALTH group?

This would involve for example becoming involved in participating in a workshop and writing an "Explanation and Elaboration" document

- ○ yes
- ○ no
- ○ Other:

Any other comments or questions on CONSORT EHEALTH

Your answer

STOP - Save this form as PDF before you click submit

To generate a record that you filled in this form, we recommend to generate a PDF of this page (on a Mac, simply select "print" and then select "print as PDF") before you submit it.

When you submit your (revised) paper to JMIR, please upload the PDF as supplementary file.

Don't worry if some text in the textboxes is cut off, as we still have the complete information in our database. Thank you!

Final step: Click submit !

Click submit so we have your answers in our database!

Submit Clear form

Never submit passwords through Google Forms.

This form was created outside of your domain. - Contact form owner - Terms of Service - Privacy Policy

Does this form look suspicious? Report

Google Forms
